# Ups and downs of a transcriptional landscape shape iron deficiency associated chlorosis of the maize inbreds B73 and Mo17

**DOI:** 10.1186/1471-2229-13-213

**Published:** 2013-12-13

**Authors:** Claude Urbany, Andreas Benke, Johanna Marsian, Bruno Huettel, Richard Reinhardt, Benjamin Stich

**Affiliations:** 1 Max Planck Institute for Plant Breeding Research, Quantitative Crop Genetics, 50829 Cologne, Germany; 2 Department of Biological Chemistry, John Innes Centre, Norwich NR4 7UH, UK; 3 Max Planck Genome Centre Cologne, 50829 Cologne, Germany

**Keywords:** Chlorosis, Iron deficiency, IBM population, Natural variation, QTL, RNA-Seq, *Zea mays*

## Abstract

**Background:**

Improving nutrient homeostasis is a major challenge of a sustainable maize cultivation, and cornerstone to ensure food supply for a growing world population. Although, iron constitutes an important nutrient, iron availability is limited. In this respect, iron deficiency associated chlorosis causes severe yield losses every year. Natural variation of the latter trait has yet not been addressed in maize and was therefore studied in the present analysis.

**Results:**

In this study, we i) report about the contrasting chlorosis phenotypes of the inbreds B73 and Mo17 at 10 and 300 μM iron regime, ii) identified over 400 significantly regulated transcripts (FDR < 0.05) within both inbreds at these growth conditions by deep RNA-Sequencing, iii) linked the gained knowledge with QTL information about iron deficiency related traits within the maize intermated B73 by Mo17 (IBM) population, and iv) highlighted contributing molecular pathways. In this respect, several genes within methionine salvage pathway and phytosiderophore synthesis were found to present constitutively high expression in Mo17, even under sufficient iron supply. Moreover, the same expression pattern could be observed for two putative bHLH transcription factors. In addition, a number of differentially expressed genes showed a co-localisation with QTL confidence intervals for iron deficiency related traits within the IBM population.

**Conclusions:**

Our study highlights differential iron deficiency associated chlorosis between B73 and Mo17 and represents a valuable resource for differentially expressed genes upon iron limitation and chlorosis response. Besides identifying two putative bHLH transcription factors, we propose that methionine salvage pathway and sterol metabolism amongst others; underlie the contrasting iron deficiency related chlorosis phenotype of both inbreds. Altogether, this study emphasizes a contribution of selected genes and pathways on natural trait variation within the IBM population.

## Background

Maize is one of the most widely grown crop plants worldwide and has become Africa's most important staple food crop [1]. In regard of a growing world population and the increasing demand for food supply, a sustainable agriculture is of first priority. One topic concerning a sustainable maize cultivation is the improvement of its ability to cope with limiting nutrient supply.

As iron-sensitive crop maize asks for i) investigating the iron deficiency associated chlorosis that different maize genotypes display, ii) identifying the underlying genes and molecular processes, and iii) using this information to improve iron deficiency chlorosis of maize through breeding.

The processes that plants employ to efficiently access iron as well as genotypic variation of iron homeostasis itself are hitherto not completely understood. Knowledge on natural variation of iron deficiency and the chlorosis response is crucial to improve growth of crops in marginal soils, where iron deficiency frequently limits growth. Iron deficiencies are found mainly on calcareous soils [2] but also develop in acid soils [3,4]. As iron is involved in the production of chlorophyll, deficiency is easily recognized by the occurrence of chlorosis symptoms, notably yellowish interveinal tissue on the younger upper leaves [3,5]. Iron constitutes an indispensable plant nutrient and severe deficiencies cause leaves to turn completely yellow or almost white, which in turn strongly impairs plant growth and leads to high yield losses [3,5]. This furthermore, influences nutritional crop quality and impacts on economic aspects like the need of fertilizers and the accessibility of growth areas.

Upon iron limitation, plants induce a coordinated set of responses that allow maximizing iron mobilization and uptake from the soil. Moreover, internal iron stores are utilized to allocate iron where crucial cellular processes are proceeding. The majority of plants except the grasses use the strategy I response to solubilize and absorb iron into roots when iron is limiting [6]. As result, Fe(III) is converted into Fe(II) and subsequently transported over the plasma membrane into root cells [6,7]. Maize and the other grasses have adopted another strategy to assimilate iron that relies on the secretion of iron chelators, non-proteinogenic amino acid derivatives, into the rhizosphere that form stable Fe(III) chelates [6]. These Fe(III)/chelator complexes, are then subsequently imported by a specific transporter (YS1) that is located at the root surface [8,9]. However, the further allocation of iron, its pool sizes and the fluxes within and between cells, tissues or whole organs as well as the regulating mechanisms, which orchestrate iron homeostasis, still remain elusive.

Hitherto, the knowledge on maize iron homeostasis and the involved genes is mainly derived from mutant studies or the molecular genetic analysis of monogenic differences in graminaceous species, such as rice [6,8,10-14]. Despite, the considerable number of identified genes and pathways in maize, their contribution to the natural phenotypic variation as well as their impact on modulating environmental adaptation is unknown. The investigation of iron deficiency chlorosis in maize using deep RNA sequencing (RNA-Seq) would allow considering the complexity of genotype and treatment related transcriptional differences [15,16]. In this respect, identified transcriptional differences might either represent causal genes for natural variation of iron mobilization and allocation and the associated chlorosis response in maize or be regulated by the latter. Linkage of the gained information with quantitative trait loci (QTL) studies for iron efficiency related traits in the IBM population (Benke et al., unpublished) allows to pin-point differentially regulated genes that co-localize with QTL confidence intervals and thereby represent excellent candidate genes underlying this trait.

The present study investigates the transcriptomes of the maize inbreds B73 and Mo17, which differ significantly in their chlorosis response upon low iron concentration (10 μM), in order to identify genotype and/or treatment related differentially expressed transcripts. The differences in regulation of these transcripts were further validated by qRT-PCR and the corresponding expression patterns at both, limiting (10 μM) and sufficient (300 μM) iron concentration were integrated in a physiological context. Furthermore, a pathway analysis allowed us to substantiate the impact of specific pathways and related genes onto iron deficiency associated chlorosis. Altogether, the proposed genes present interesting candidates that might contribute to natural variation and could be the basis for further functional analysis and genetic proceedings in diverse natural populations.

## Results

### Impact of iron regime on the phenotype of the maize inbreds B73 and Mo17

**Figure 1:**
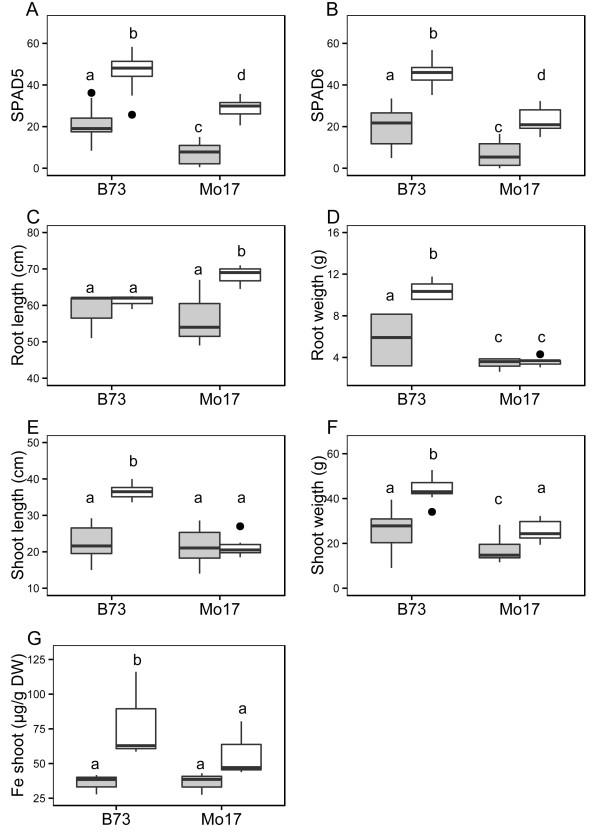
**Phenotypic differences of the maize inbred B73 and Mo17 at 300 and 10 μM iron regime.** The distribution of phenotypic values is shown for both inbreds for **A)** SPAD values of the 5^th^ leaf, **B)** SPAD values of the 6^th^ leaf, **C)** root length in cm, **D)** root weight in g, **E)** shoot length in cm, **F)** shoot weight in g, and **G)** the iron content in the shoot in μg/g shoot dry weight (SDW) at 10 μM iron (light grey) and 300 μM iron (white) regime. Outliers are represented by black dots. Different letters indicate significant differences between samples as determined by a two-sided pairwise t-test with p < 0.05.

After hydroponic growth at limiting (10 μM Fe) and sufficient iron (300 μM Fe) regime, the maize inbreds B73 and Mo17 were evaluated for the relative chlorophyll content of the 5^th^ and the 6^th^ leaf, determined by measuring SPAD units, as well as length and weight of root and shoot, and the iron content in the shoot. Significant differences between genotypes and treatments were observed (Figure 1). In this respect, Mo17 showed significantly lower SPAD values for the 5^th^ as well as the 6^th^ leaf in comparison to B73. These differences were not only observable at limiting iron concentration but also at sufficient iron supply. All over, B73 showed significantly weaker chlorosis symptoms than Mo17 (Figure 1A, B). Interestingly, absolute root length was higher for Mo17 than for B73 at 300 μM iron. Whereas B73 showed no severe decrease in response to iron limitation, Mo17 root length decreased to even lower values than B73 (Figure 1C). Despite higher root length of Mo17 at 300 μM iron, root weight was drastically lower at both iron regimes when compared to B73 (Figure 1D). Furthermore, B73 was characterized by a significantly larger shoot height as Mo17 at 300 μM iron (Figure 1E). Limiting iron supply led to about equal shoot length values for both genotypes (Figure 1E). Shoot weight was significantly different between the two inbreds at both iron regimes, with B73 having a higher shoot mass than Mo17 (Figure 1F). Finally, the iron content of the shoot was higher for B73 at 300 μM iron than for Mo17 but decreased for both genotypes at similar levels upon limitation (Figure 1G).

In conclusion, B73 was characterized by a more vigorous growth under sufficient iron conditions and lower leaf chlorosis under limiting iron (Figure 1 and Additional file 1: Figure S1A). In addition, B73 samples at 300 μM iron treatment showed highest iron content and biomass trait values, except for root length, which was highest in Mo17 plants at 300 μM iron (Additional file 1: Figure S1A, B). As determined by principal component analysis of trait values, samples of both inbreds at 300 μM iron were followed by samples of B73 and finally Mo17 samples grown at 10 μM iron (Additional file 1: Figure S1B). When comparing growth at 10 versus 300 μM iron, the decrease in trait values was higher for shoot and root traits as well as for iron content of the shoot in B73 than for Mo17. Contrary, the decrease in SPAD values and correspondingly stronger leaf chlorosis was observed in Mo17 than B73 (Additional file 1: Figure S1A).

### The iron responsive root transcriptome of B73 and Mo17

**Figure 2:**
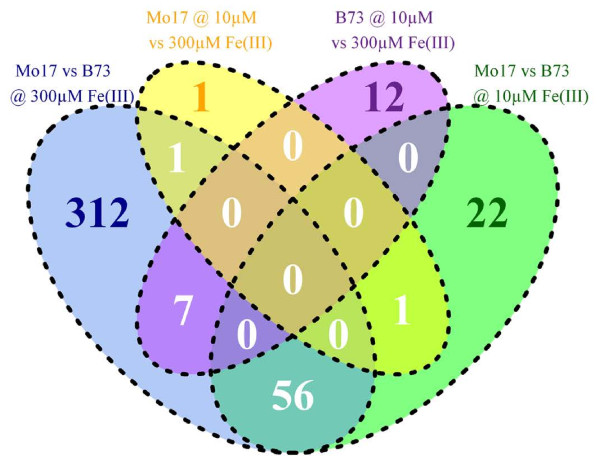
**Significantly regulated genes (experiment wide FDR < 0.05) across two-way comparisons.** The corresponding number of transcripts is indicated for each space of the Venn diagram. Comparison 1, representing transcripts differentially expressed between both inbreds at 300 μM iron is shown in blue. Comparison 2, representing transcripts differentially expressed between both inbreds at 10 μM iron is shown in green. The treatment specific comparisons of Mo17 at 10 μm versus 300 μM iron (Comparison 3) and of B73 at 10 μm versus 300 μM iron (Comparison 4) are shown in orange and purple, respectively.

Root tissue of two biological replicates was collected at two iron concentrations (10 and 300 μM) in order to determine the differences in the transcriptome of B73 and Mo17. RNA-Seq, resulted in a median library size of 7.6 million reads after quality control (Additional file 1: Table S1). Differentially expressed genes were called in four specific two-way comparisons. The two genotype relative comparisons consisted of analysing the expression profile of root tissue from Mo17 versus tissue from B73 grown at 300 μM (Comparison 1) and at 10 μM iron (Comparison 2). For treatment relative comparisons transcriptome signatures of root tissue from Mo17 (Comparison 3) and B73 (Comparison 4) grown at 10 μM was compared with such resulting from root tissue of the same genotype collected at 300 μM. All log_2_ fold changes (log_2_FC) refer to the expression values at condition 1 versus condition 2 (Table 1). In this respect, a positive log_2_FC in Comparison 1 (Comp. 1) reflects a higher expression in Mo17 grown at 300 μM iron than in B73. Differential expression analysis using four different approaches, namely the *cufflinks* pipeline with or without RABT [17] assembly as well as the count table based methods provided by the *R* packages *DeSeq*[18] and *edgeR*[19] yielded a similar number of differentially expressed transcripts (FDR < 0.05). Analysis by *DeSeq* was most stringent as reflected by the lowest number of transcripts with significant expression differences in two-way comparisons. Interestingly, every detection method resulted in unique transcripts (Additional file 1: Figure S2). Moreover, transcripts significant at an FDR < 0.05 in all four statistical approaches could be identified. Such transcripts were called for each of the four two-way comparisons and constituted the final set of the significantly regulated transcripts that was further investigated. None of these transcripts was shared by all four two-way comparisons (Figure 2). Altogether, 412 unique transcripts were identified as being differentially expressed (FDR < 0.05) in all comparisons, with the highest number of such transcripts in the comparison between Mo17 and B73 at 300 μM (1). Among 376 significantly regulated genes within this comparison, 127 showed a higher expression in Mo17 and a median fold change higher than 2, whilst 244 showed higher expression at the same fold change in B73 (Table 1). The transcriptomic comparison of these two genotypes at 10 μM iron supply yielded 79 significant transcripts, with 31 being more abundant in Mo17 and 48 with higher expression values for B73 (Table 1). Interestingly, only 3 transcripts were detected as differentially expressed at an FDR < 0.05 for the expression profiles of Mo17 grown at 10 μM vs. 300 μM iron (Table 1). The iron deficiency chlorosis resistant genotype B73 induced 13 out of 19 significant genes under iron-limiting conditions. Out of the 19 iron responsive B73 genes, seven showed a differentially expression at non-limiting iron concentration between both genotypes (Table 1 and Figure 2). None of 19 genes was significantly responding to iron starvation in Mo17 (Figure 2). Principal component analysis of transcripts and their expression pattern amongst the four two-way comparisons (Additional file 1: Figure S1C) resulted in a clear sample clustering, separating genotype (PC1) and treatment (PC2). However, clear differentiation of iron treatments was more pronounced for B73 as for Mo17 samples (Additional file 1: Figure S1C).

**Table 1:** Differentially regulated transcripts that are significant at an experiment wide FDR <0.05 at the corresponding conditions

**Comparison**	**Condition 1 vs. condition 2**	**Total # significantly regulated transcripts**^**1**^	**Up-regulated**^**2 **^**(in condition 1 vs. 2)**	**Down-regulated**^**3 **^**(in condition 1 vs. 2)**
1	Mo17 vs. B73 @ 300 μM	376	127	244
2	Mo17 vs. B73 @ 10 μM	79	31	48
3	Mo17 @ 10 μM vs. 300 μM	3	2	1
4	B73 @ 10 μM vs. 300 μM	19	13	6

^**1**^Experiment wide FDR < 0.05.

^**2**^Experiment wide median fold change > 2.

^**3**^Experiment wide median fold change < 0.5.

### Significantly regulated transcripts co-localize with relevant trait QTLs

**Figure 3:**
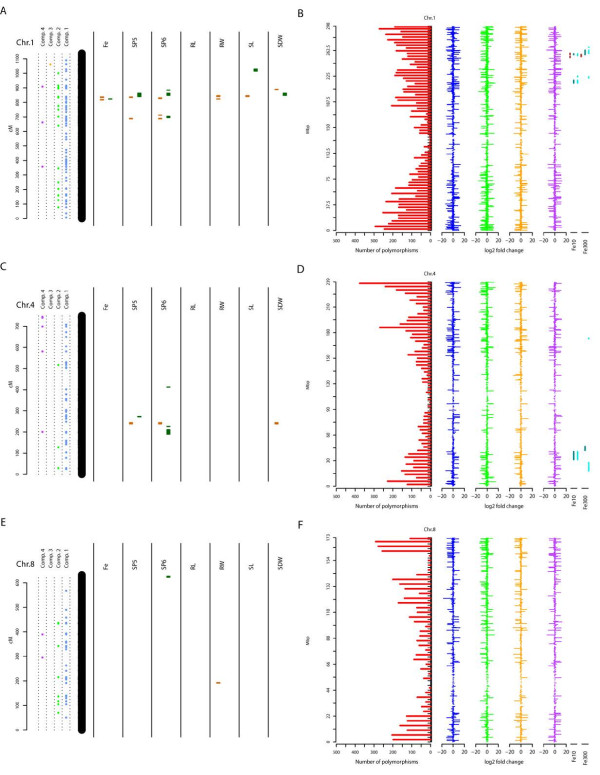
**QTL co-localization of significantly regulated genes and polymorphic transcriptional landscape between B73 and Mo17.** QTL confidence intervals are indicated for corresponding traits (Fe = iron content in the shoot; SP5 = SPAD values of the 5^th^ leaf; SP5 = SPAD values of the 6^h^ leaf, RL = root length; RW = root weight; SL = shoot length; SDW = shoot dry weight) as orange boxes for the 10 μM iron regime and in dark green for the 300 μM iron regime as identified by Benke et al., (unpublished). Genetic distances are given in cM and were retrieved from marker information available at http://www.maizegdb.org for the IBM population. For projection of differentially regulated genes within specific two-way comparisons (Table 1) the available physical transcription start site positions (http://www.maizegdb.org) were converted into putative genetic positions by interference from adjacent marker positions. Candidate genes for Chromosome (Chr.) 1 **(A)**, 4 **(C)** and 8 **(E)** were projected onto the genetic map. The equivalent polymorphic and transcriptional landscape is displayed for Chr.1 **(B)**, 4 **(D)** and 8 **(F)**. The number of polymorphisms (SNPs and INDELs) is given for artificial bins of a size of 4Mbps. The median log2FC for transcripts detected across all statistical approaches is given for specific two-way comparisons (Comp. 1 = blue; Comp. 2 = green; Comp. 3 = orange; Comp. 4 = purple) at the relative physical position, represented by the transcriptional start site. In the case of absent/present expression data, log2FC of ±∞ were exchanged by ±20 (the highest log2FC across the experiment). QTLs confidence intervals for iron content in the shoot (red) and SPAD values of the 5^th^ (turquoise) and the 6^th^ leaf (light cyan) are indicated at their interpolated physical position for the 10 (Fe10) and 300 μM iron (Fe300) regime.

When projecting the final set of significantly regulated on the genetic map, an even distribution across all chromosomes and over their whole length could be observed (Figure 3). Besides, several known candidate genes for iron deficiency repsonse in maize, several of the differentially regulated transcripts located adjacent or within QTL confidence intervals for iron homeostasis in the IBM population (Figure 3, Table 2, Additional file 1: Figure S3). Most of the regulated transcripts were present either in the comparisons of both genotypes in the 300 μM iron regime or when comparing B73 grown at 10 μM to 300 μM iron (Figure 3 and Table 2). The regulated transcripts that mapped to QTLs on maize chromosomes at 300 μM iron, tagged such detected for SPAD values of the 5^th^ and the 6^th^ leaf as well as shoot weight (Table 2). Only a single gene co-localizes with a QTL for shoot length on chromosome I (Table 2). In the case of 10 μM iron regime, the significantly regulated transcripts again co-localized with QTLs for SPAD values of the 5^th^ and 6^th^ leaf, and shoot weight (Table 2). In addition also QTLs for shoot length as well as root weight were tagged. Two transcripts that showed a differential expression between B73 roots harvested at 300 and 10 μM iron mapped to QTL intervals for SP6 at 300 μM iron and SDW at 10 μM, respectively (Table 2).

**Table 2:** Differentially regulated transcripts that co-localize with QTLs for iron efficiency related traits (Benke et al. unpublished)

				**QTL@300 μM**^**5**^			**QTL@10 μM**^**6**^		
**Gene**^**1**^	**Position**^**2**^	**Function**^**3**^	**UniProtKB/TrEMBL**^**4**^	**Comp. 1**^**7**^	**Comp. 2**	**Comp. 4**	**Comp. 1**	**Comp. 2**	**Comp. 4**
GRMZM2G003304	1:218716368-218721179	Uncharacterized protein	B7ZYZ9				SP5,SP6		
GRMZM2G043127	1:253796236-253799897	Uncharacterized protein	B4FCR0				RW		
GRMZM2G027663	1:256566871-256573278	Uncharacterized protein	C0PEH3	SP5	SP5		SP5,SP6,RW,SL	SP5,SP6,RW,SL	
GRMZM2G118821	1:258353072-258355277	Uncharacterized protein	B6U8Z3	SP5,SP6,SDW	SP5,SP6,SDW		RW,SL	RW,SL	
GRMZM2G423972	1:264155924-264160052	NA	NA				SDW		SDW
GRMZM2G042133	1:265023533-265154204	NA	NA				SDW	SDW	
GRMZM2G127521	1:268343581-268346959	NA	NA				SDW	SDW	
GRMZM2G583462	1:269468231-269469313	Uncharacterized protein	K7UIK7					SDW	
GRMZM2G049790	1:289274299-289275170	Putative uncharacterized protein	B6TNU7	SL					
GRMZM2G054905	3:191124530-191185971	NA	NA	SP5	SP5				
GRMZM2G057140	3:191413501-191495940	NA	NA	SP5	SP5				
GRMZM2G127665	3:196137227-196147905	NA	NA	SP5					
GRMZM2G574782	4:18676198-18682894	Probable bifunctional methylthioribulose-1-phosphate dehydratase/enolase-phosphatase E1	B4G0F3	SP6		SP6			
GRMZM2G014902	4:32498151-32531144	Putative MYB DNA-binding domain superfamily protein	K7U156				SP5,SP6,SDW		
GRMZM2G157443	4:37937136-37938763	60S acidic ribosomal protein P1	B4FUB4				SP5,SP6,SDW		
GRMZM2G112792	4:38235714-38238075	Uncharacterized protein	K7U1M0				SP5,SP6,SDW		
GRMZM2G097395	4:44953328-44958944	Nodulin-like protein	K7USY0	SP5					
GRMZM2G179294	5:180731982-180733210	High-affinity nitrate transport	Q0VH26				RW		
GRMZM2G088469	7:17179983-17182140	Putative uncharacterized protein	B6TKI8				SDW		
GRMZM2G140342	7:121316023-121319306	TMEM87A protein; uncharacterized protein	B4F8U5	SP5					
GRMZM2G118119	7:121710274-121712960	Uncharacterized protein	K7UXC8	SP5					
GRMZM2G477325	7:168401561-168403148	Hydrophobic protein LTI6, uncharacterized protein	B4FFP9	SDW					
GRMZM2G039757	7:168744977-168747272	NA	NA	SDW					
GRMZM2G047139	8:24719638-24722490	Esterase; uncharacterized protein	B6TZ91				RW		
GRMZM2G154278	8:26066129-26069719	Pre-mRNA-splicing factor cwc15	B6T6R6				RW		
GRMZM2G123143	8:40678211-40682507	Uncharacterized protein	K7VC64				RW		
GRMZM2G061052	8:43287734-43288541	Putative HLH DNA-binding domain superfamily protein; uncharacterized protein	B4FNT9				RW		

^**1**^Gene identifiers as retrieved from http://www.maizesequence.org - reference annotation file ZmB73_5a.

^**2**^Physical positions as retrieved from http://www.maizesequence.org - maize sequence ZmB73_RefGen_v2.

^**3**^Putative function (database information from http://www.maizesequence.org).

^**4**^UniProtKB/TrEMBL accession number.

^**5**^QTL for corresponding trait detected at 300 μM iron regime (Benke et al., unpublished).

^**6**^QTL for corresponding trait detected at 10 μM iron regime (Benke et al., unpublished).

^**7**^Corresponding two-way Comparison in which genes were identified as being significantly (FDR < 0.05) regulated.

QTL abbreviations: SP5 = SPAD of the 5^th^ leaf; SP6 = SPAD of the 6^th^ leaf, RW = root weight; SDW = shoot dry weight; SL = shoot length.

### GO-term enrichment and pathway analysis

**Figure 4:**
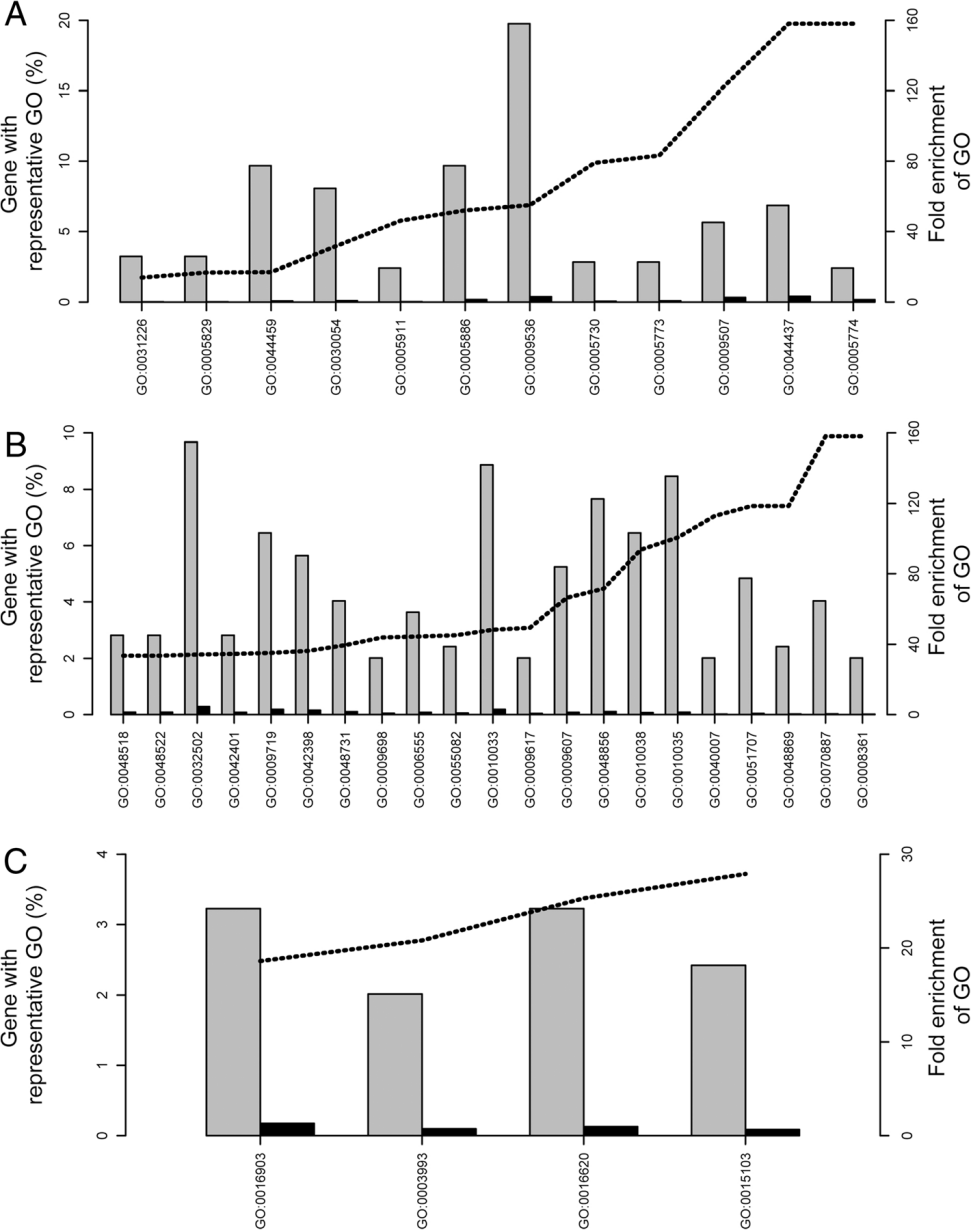
**GO-term analysis of the significantly regulated genes across all two-way comparisons.** Significantly most enriched GO-terms (FDR < 0.05) for cellular component **(A)**, biological process **(B)** and molecular functions GOs **(C)** are plotted according to increasing enrichment (dotted line) of the percentage of genes in the RNA-Seq gene set (grey bars) compared to that of the maize reference set (black bars). Cellular Component GOs - GO:0031226 intrinsic to plasma membrane, GO:0005829 cytosol, GO:0044459 plasma membrane part, GO:0030054 cell junction, GO:0005911 cell-cell junction, GO:0005886 plasma membrane, GO:0009536 plastid, GO:0005730 nucleolus, GO:0005773 vacuole, GO:0009507 chloroplast, GO:0044437 vacuolar part. GO:0005774 vacuolar membrane. Biological Process GOs - GO:0048518 positive regulation of biological process, GO:0048522 positive regulation of cellular process, GO:0032502 developmental process, GO:0042401 cellular biogenic amine biosynthetic process, GO:0009719 response to endogenous stimulus, GO:0042398 cellular amino acid derivative biosynthetic process, GO:0048731 system development, GO:0009698 phenylpropanoid metabolic process, GO:0006555 methionine metabolic process, GO:0055082 cellular chemical homeostasis, GO:0010033 response to organic substance, GO:0009617 response to bacterium, GO:0009607 response to biotic stimulus, GO:0048856 anatomical structure development, GO:0010038 response to metal ion, GO:0010035 response to inorganic substance, GO:0040007 growth, GO:0051707 response to other organism, GO:0048869 cellular developmental process, GO:0070887 cellular response to chemical stimulus, GO:0008361 regulation of cell size. Molecular Function GOs - GO:0016903 oxidoreductase activity, acting on the aldehyde or oxo group of donors, GO:0003993 acid phosphatase activity, GO:0016620 oxidoreductase activity, acting on the aldehyde or oxo group of donors, NAD or NADP as acceptor, GO:0015103 inorganic anion transmembrane transporter activity.

A GO-term enrichment analysis of the 412 significantly regulated genes within all two-way comparisons yielded several cellular, biological and functional enriched GOs (Figure 4). In this respect, over-represented cellular component GOs were related to chloroplast, the vacuole and membranous components (Figure 4 and Additional file 1: Table S2). Concerning the GOs related to biological processes, these can be roughly grouped to developmental processes, regulation of cell size, and responses to biotic and abiotic stimulus as well as, nitrogen, sulphur, carboxylic acid and amino acid synthetic related processes (Figure 4 and Additional file 1: Table S2). Especially, methionine and aspartate biosynthetic as well as catabolic process related GOs were overrepresented within the significant gene set. In addition, GOs of response to metal ions, ion homeostasis, anion transport and inorganic anion transport were also found to be enriched. Within the GOs associated with molecular function, oxidoreductase, inorganic anion transport, and phosphatase activity were significantly enriched (P < 0.05).

**Figure 5:**
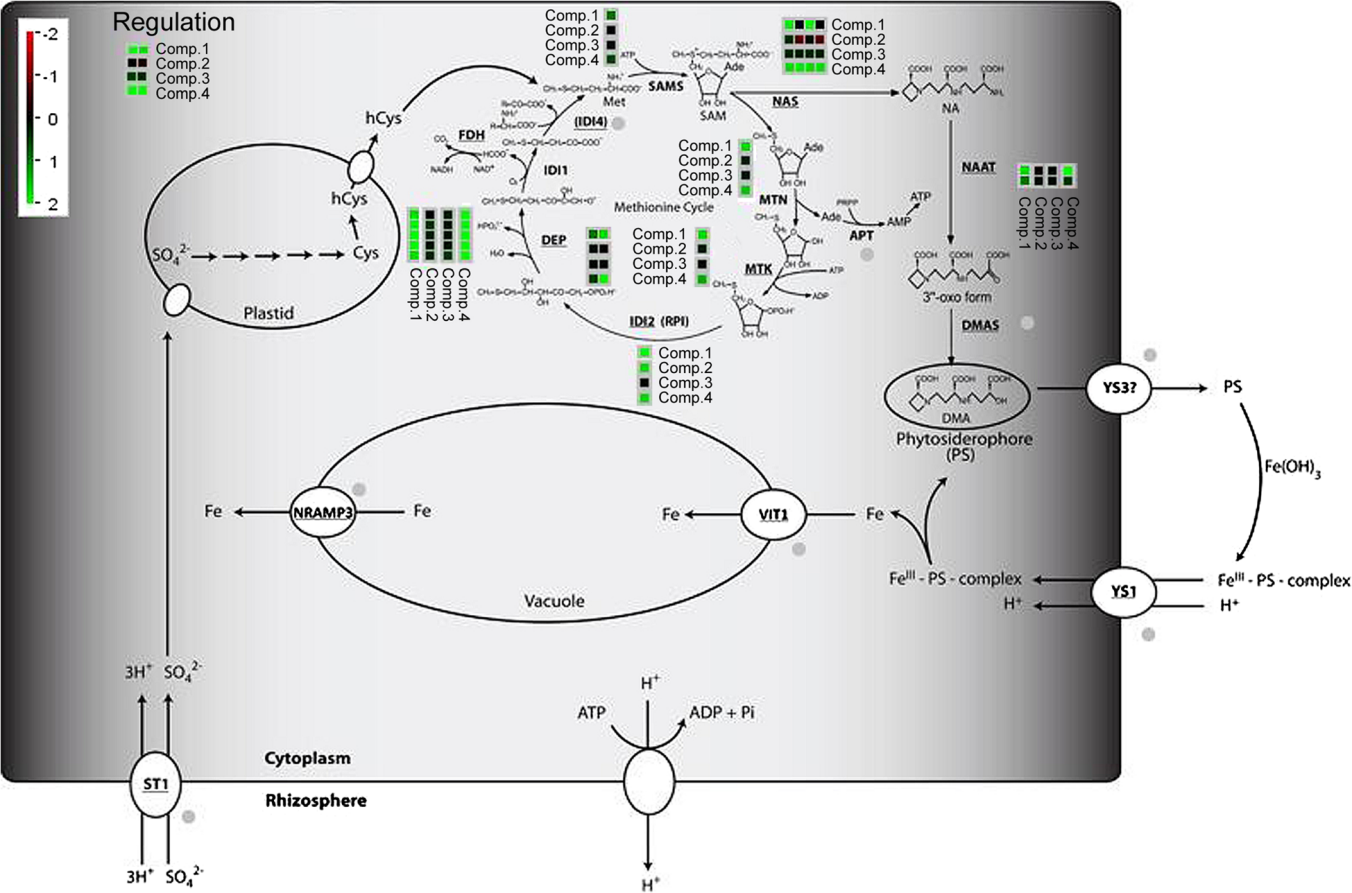
**Pathway analysis of iron deficiency associated chlorosis.** Median log_2_FC across all statistical approaches are depicted by transcripts bins adjacent to corresponding genes, using MapMan and custom pathway [5] as well as mapping files (Additional file 1 and Additional file 2). Regulation of genes within relative Comparisons (1–4) are color-coded (red = lower expression in condition 1 vs. 2; green = higher expression in condition 1 vs. 2). Multiple bins next to respective transcripts indicate multiple isoforms. Abbreviations: Fe = Iron; PS = Phytosiderophores; *YS1* = yellow stripe 1; *YS3* = yellow stripe 3; *ST1* = Sulfate transporter 1; SO_4_^2-^ = sulfate; Cys = cysteine; hCys = homo-cysteine; *FDH* = formate dehydrogenase; *DEP* = methylthioribulose-1-phosphate dehydratase-enolase-phosphatase; *IDI1* = 2-keto-methylthiobutyric-acid forming enzyme; *IDI4* = putative aminotransferase catalyzing the synthesis of methionine from 2-keto-methylthiobutyric acid; *SAMS* = S-adenosyl-methionine synthase; *MTN* = methylthioadenosine / S-adenosyl homocysteine nucleosidase; *MTK* = methylthioribose kinase; *IDI2* = eukaryotic initiation factor 2B-like methylthioribose-1-phosphate isomerase; *NAS* = nicotinamine synthase; *NAAT* = nicotianamine amino-transferase; *DMAS* = 2’-deoxymugineicacid synthase; *APT* = Adenosin phosphoribosyltransferase; *VIT1* = vacuolar iron transporter 1; NRAMP3 = natural resistance associated macrophage protein 3; Regulation = GRMZM2G057413 (homology to *OsIRO2* and GRMZM2G350312 homology to *OsIRO3* and *AtPYE)*.

A pathway analysis showed that significantly regulated genes within the comparison of both genotypes at 300 μM iron supply were involved in amino acid and polyamine metabolism (Additional file 1: Figure S4). Contrary to amino-acid metabolism related transcripts that were induced in Mo17 vs. B73 at non limiting iron supply, expression of polyamine metabolic genes was down-regulated in Mo17 (Additional file 1: Figure S4). Interestingly, transcripts involved in polyamine metabolism were found to be induced in B73 upon iron starvation. In addition, differences were also observed for transport related processes but without a clear trend in respect to genotypic or treatment related comparisons (Additional file 1: Figure S4). Intriguingly, transcripts involved in metal handling processes that were up-regulated in B73 in response to low iron concentration were already present at high expression levels in Mo17 at 300 μM iron (Additional file 1: Figure S4). Interestingly, a custom pathway analysis of phytosiderophore and strategy II iron transport related genes showed differences concerning the expression profile of the significantly regulated gene set for two-way comparisons (Figure 5). Correspondingly, expression of these genes was considerably different at non-limiting iron concentration between Mo17 and B73 (Figure 5). In contrast to B73, showing an induction of genes involved in methionine salvage pathway (YANG pathway) and phytosiderophore synthesis upon iron limitation, Mo17 already expressed these genes at high levels in the 300 μM iron regime (Figure 5).

### The polymorphic landscape of Mo17 and B73 and its transcriptional equivalent

In accordance with previous studies [20], a high degree of polymorphisms between Mo17 and B73 was observed for the majority of chromosomes (Figure 3 and Additional file 1: Figure S5). In addition, conserved genomic regions between both inbreds with low structural variation were identified (Figure 3, Additional file 1: Figure S5) as already described elsewhere [20]. In this respect, an extended region over five arbitrary bins on chromosome VIII (ca. 20 Mbps) was observed (Figure 3, Additional file 1: Figure S5). Remarkably, several strong transcriptional differences were observed in all four two-way comparisons for genes mapping to this region. A high number of polymorphisms could also be detected within the deduced QTL regions for SPAD values of the 5^th^ and the 6^th^ leaf as well as the Fe content in the shoot at both iron concentrations (Additional file 1: Figure S5). In addition, these regions harboured a large number of transcripts, characterized by high median fold changes within the two-way comparisons. The majority of these transcripts were expressed at higher levels in B73 at 300 and 10 μM iron (Additional file 1: Figure S5). Furthermore, these transcripts were induced in B73 at 10 μM and down-regulated in Mo17. The median log_2_FC of the mapped transcripts declined in the vicinity of the centromere but showed no distinct spatial pattern (Additional file 1: Figure S5).

**Figure 6:**
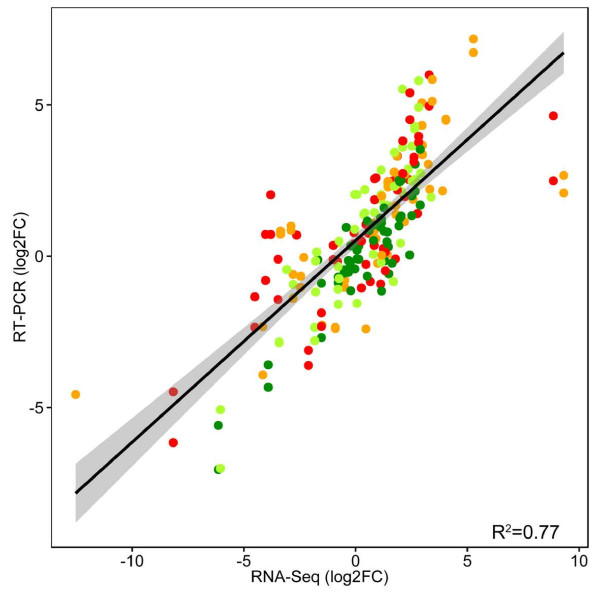
**Correlation RNA-Seq and RT-PCR data.** The relative log_2_FC for known as well as novel candidate genes (Additional file 1: Table S4) to *Actin1* (GRMZM2G126010) are given for corresponding genes. The RNA-Seq log_2_FC represents the median log_2_FC relative to *Actin1* across all statistical approaches. The log_2_FC of corresponding genes for B73 at 300 μM (dark green), B73 at 10 μM (light green), Mo17 at 300 μM (red), Mo17 at 10 μM (orange) is displayed. The Pearson correlation coefficient (R^2^) as well as a linear regression line with confidence interval is indicated.

The validation of differences within the transcriptome of the two maize inbreds B73 and Mo17 by qRT-PCR showed a significant correlation (R^2^ = 0.77) for a selection of 40 genes (Figure 6 and Additional file 1: Table S4). When focusing on specific candidate genes, divergent expression patterns were observed (Table 3). The selection of candidate genes comprised genes significantly regulated in the comparison of B73 grown at 300 and 10 μM iron, except nicotianamine synthase 1 (*NAS1)* and formate dehydrogenase (*FDH)*, which were represented by multiple isoform (Additional file 1: Table S3). However, it is noteworthy that *NAS1*[21] was found to be represented by two loci (GRMZM2G385200 and GRMZM2G034956), which were significantly regulated in Comp. 4. A further analysis manifested five homologous *NAS1* loci in maize that could represent different isoforms arising from duplication events (Additional file 1: Figure S6).

**Table 3:** Candidate genes significantly regulated within the RNA-Seq experiment (FDR < 0.05) and validated by RT-PCR

**Gene**	**Position**	**Function**^**1**^	**Accession**^**2**^	**Comp. 1**	**Comp. 2**	**Comp. 3**	**Comp. 4**
				**(log2FC)**	**(log2FC)**	**(log2FC)**	**(log2FC)**^**3**^
GRMZM2G350312	1:65657063-65660290	bHLH transcription factor	AT3G47640.1(PYE)/LOC_Os03g26210.2/B8A2P8	sig. (1.81)			sig. (3.07)
GRMZM2G106980	1:203647045-203653762	Putative BURP-domain protein, uncharacterized protein	AT5G25610.1(ATRD22,RD22)/LOC_Os06g19800.1/B8A121	sig. (-3.80)	sig. (-4.25)		
GRMZM2G059465	1:209119390-209120903	O-methyl transferase, uncharacterized protein	AT4G35160.1/LOC_Os06g16960.1/B8A095				sig. (-2.49)
GRMZM2G055802	1:293997055-290998873	NA	LOC_Os03g60840.1			sig. (2.53)	
GRMZM2G132678	3:20795800-20806607	Hydroxycinnamoyl-CoA shikimate/quinate hydroxycinnamoyl transferase	AT5G48930.1(HCT)/LOC_Os08g43040.1	sig. (5.35)	sig. (4.50)		
GRMZM2G057413	3:148031451-148062994	bHLH transcription factor	AT3G56970.1(BHLH038,ORG2)/LOC_Os01g72370.1				sig. (3.77)
GRMZM2G400602	3:174361241-174363882	Putative oligopeptide transporter, uncharacterized protein	AT1G22540.1/LOC_Os01g65100.1/C0HEP6				sig. (3.29)
GRMZM5G896496	3:211872426-211879044	Putative chaperone ClpC1, uncharacterized protein	C0HH28	sig. (-5.10)	sig. (-5.00)		
GRMZM2G574782	4:18676198-18682894	Probable bifunctional methylribose-1-phosphate dehydratase/enolase phosphatase E1	AT5G53850.2/LOC_Os11g29370.1/B4G0F3	sig. (1.74)			sig. (1.92)
GRMZM2G096958	4:219581607-219585123	Nicotinamine aminotransferase1, uncharacterized protein	AT5G36160.1/LOC_Os02g20360.1/B4FEZ7	sig. (1.71)			sig. (1.98)
GRMZM2G010280	4:240232549-240234575	High affinity nitrate transporter	AT5G60770.1(ATNRT2.4,NRT2.4/LOC_Os02g02170.1)/Q53CL7				sig. (-3.12)
GRMZM2G010251	4:240243499-240245403	High affinnity nitrate transporter, uncharacterized protein	AT1G08090.1(ACH1,ATNRT2.1)/LOC_Os02g02170.1/B4FSV9				sig. (-2.58)
GRMZM2G089836	567508591-67511890	Glycosyl hydrolase, Invertase	AT1G62660.1/LOC_Os02g01590.1/B6T0A9				sig. (-2.61)
GRMZM2G041980	5:148304652-148307020	Putative aquaporin protein, NIP1-1	AT4G18910.1(ATNLM2,NIP1)/LOC_Os05g11560.1/Q9ATN4	sig. (3.46)		sig. (-2.41)	
GRMZM2G465226	7:3483731-3484794	Pathogenesis related protein 1 (PR-1), uncharacterized protein	AT5G57625.1: CAP/LOC_Os07g03710.1/B4FVP5		sig. (3.71)	sig. (1.83)	
GRMZM2G099049	7:120764735-120766193	Auxin/Dormancy related protein	AT1G56220.2/LOC_Os09g26620.1				sig. (-2.60)
GRMZM2G336694	8:10989824-10990619	NA	NA	sig. (3.67)	sig. (3.38)		
GRMZM2G153977	8:70995815-70998984	Eukaryotic aspartyl protease	AT1G62290.1/LOC_Os05g49200.1		sig. (-4.24)		
GRMZM2G096029	8:104279085-104281118	Obtusifoliol 14α demethylase	AT1G11680.1(CYP51)/LOC_Os05g34325.1				sig. (-2.91)
GRMZM2G135536	10:893975-895713	Putative cytochrome P450	AT1G64940.1(CYP89A6)/LOC_Os10g05020.1	sig. (3.51)	sig. (4.24)		
GRMZM2G133721	10:111856375-111861824	Putative enolase	NA	sig. (7.65)	sig. (6.36)		

^**1**^Annotations from http://www.maizegdb.org.

^**2**^Accession number for the homologous sequences from *Arabidopsis thaliana*, *Oryza sativa* (database information from http://www.maizegdb.org), and UniProtKB/TrEMBL.

^**3**^log2FC for the corresponding comparison in which the gene was identified as being differentially expressed (significant at FDR < 0.05) positive log2FCs reflect higher expression values in Condition2 whilst negative log2FC refer to higher expression in Condition 1 of specific comparisons (cf Table 1).

**Figure 7:**
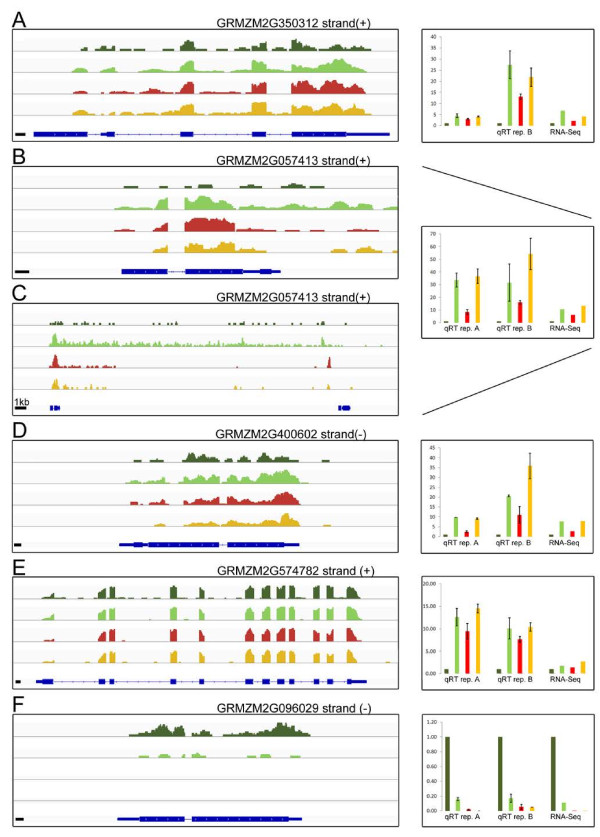
**RNA-Seq read coverage of candidate genes and qRT-PCR validation.** Displayed are the un-normalized coverage tracks for transcripts with respective gene loci **A)** putative bHLH transcription factor, **B)** and **C)** putative bHLH transcription factor, **D)** putative oligopeptide transporter, **E)** probable bifunctional methylribose-1-phosphate dehydratase/enolase phosphatase E1, **F)** obtusifoliol 14α demethylase (more information can be found in Table 3) on the left hand side for B73 at 300 μM iron (dark green), B73 at 10 μM iron (light green), Mo17 at 300 μM iron (red), and Mo17 at 10 μM iron (orange) with the corresponding gene model at the bottom. If not indicated, the black bar represents 100 bps. In addition, the strand orientation is displayed (+ = sense strand; - = antisense strand). On the right hand side, the expression pattern as detected by RNA-Seq for all biological replicates is displayed as fold change (FC) relative to *Actin1* (GRMZM2G126010) expression. Validation of the expression pattern by RT-PCR within each biological replicate is shown as FC relative to *Actin1* expression with standard deviations.

Moreover, the expression level of two putative factors, which showed significant expression differences in Comp. 4 and with high homology to *AtPYE/OsIRO3* and *OsIRO2* respectively [22,23], displayed an induction within B73 upon low iron conditions. Interestingly, these genes showed elevated transcript levels in Mo17 independent of the iron regime. Surprisingly, the *IRO2* homologuous gene was characterized by a high RNA-Seq read coverage over a length of nearly 20 kb downstream of the putative transcription start site (Figure 7 and Additional file 1: Figure S7). This coverage was significantly lower in Mo17 even though the read counts within the gene model of the reference sequence were comparable to those observed in B73 upon induction. Moreover, several genes encoding putative transporters were significantly regulated in Comp. 3 and 4. It is noteworthy that a putative oligopeptide transporter (GRMZM2G400602) was up-regulated in B73 upon limiting iron in contrast to putative nitrate transporters, which displayed a down-regulation of their expression at 10 μM iron in B73 (Figure 7, Table 3 and Additional file 1: Figure S8). In addition, significant genes within the comparison of Mo17 grown at 300 and 10 μM as well as genes at the comparisons of both genotypes at 300 and 10 μM iron showing an experiment wide median P-value within the fourth quartile were included in a further analysis and validation by qRT-PCR. In this respect, a putative *NIP-1* homologous aquaporin was amongst the significant genes in Comparison 3 and showed a down-regulation upon limiting iron supply in the RNA-Seq analysis. However, the expression pattern could not be validated for this *NIP-1* homologue (Figure 7, Table 3 and Additional file 1: Figure S9). Transcripts encoding putative cytochrome P450, known to be involved in secondary metabolism, were up-regulated in Mo17 at 300 and 10 μM iron in comparison to B73 (Figure 7, Table 3 and Additional file 1: Figures S7 and S10). Remarkably, a putative obtusifoliol 14-alpha demethylase like gene showed a strong and significant down-regulation in B73 upon limiting iron (Figure 7, Table 3 and Additional file 1: Figure S10). As stated above, genes involved in methionine salvage pathway and phytosiderophore synthesis (*NAAT* and *DEP1)* were up-regulated in B73 upon low iron regime and already displayed high transcript levels in Mo17 under 300 μM iron (Figure 7, Table 3 and Additional file 1: Figure S8). In addition, methylribose-1-phosphate isomerase displays higher expression levels in Mo17 throughout both iron regimes than B73, which again only induces this gene upon iron starvation.

Another interesting finding consists in a transcript encoding a putative BURP-domain protein [24], which is down-regulated in its expression within B73 at the 10 μM iron regime. This transcript is characterized by a very low expression in Mo17 independent of the applied iron conditions and displays exon-skipping when compared to B73 (Figure 7, Table 3 and Additional file 1: Figure S7). Finally, transcripts encoding uncharacterized proteins, O-methyl- and glycosyl-transferases, a dormancy related and auxin responsive gene, a *ClpC1* homologue, an enolase, and an aspartic peptidase were validated by qRT (Figure 7, Table 3 and Additional file 1: Figures S7, S8, S9 and S10). Pseudogenes, transposable elements and transcripts giving rise to very small peptides (< 50AA) were omitted from a closer analysis.

## Discussion

### Phenotypic responses upon iron regime

As outlined above, the maize inbreds B73 and Mo17 showed a contrasting chlorosis response upon limiting iron regime (Figure 1A, B, Additional file 1: Figure S1A, B). Our observations might reflect that Mo17 cannot deal as efficiently as B73 with available iron, even far above limiting concentrations. In addition, the evaluation of the morphological values of shoot and root showed a stronger morphological impact of decreasing iron concentration for Mo17 than for B73 (Figure 1C-F, Additional file 1: Figure S1A, B). These might also be due to an altered iron homeostasis of Mo17, which even at 300 μM tries to maximise iron uptake by increasing root length as well as branching (Benke et al., unpublished). Despite an increase of Mo17 root length, root weight stays significantly lower at both iron treatments, when compared to B73. Correspondingly, the genetic basis of root differences between B73 and Mo17 might influence iron homeostasis and a potential cross-talk between the underlying pathways might exist as shown for *Arabidopsis thaliana*[22]. If higher biomass of B73 is also a result of a more efficient iron and in general nutrient homeostasis cannot be clearly answered. Interestingly, the iron content in the shoot of both genotypes was not different at limiting iron but lower in Mo17 than in B73 at 300 μM available iron (Figure 1G, Additional file 1: Figure S1A). This in consequence, might indicate that either Mo17 displays a significantly altered iron allocation, a deregulation of iron deficiency signal pathways or impairment in iron store build-up. Although, B73 is characterized by a more vigorous growth under 300 μM treatment, iron content of the shoot was not drastically increased under these conditions. Nevertheless, iron deficiency asscoiated chlorosis is significantly lower for B73 than for Mo17, which might indicate, in addition to other morphological differences, a more efficient iron homeostasis of B73. The nature of the iron deficiency signals, the linked molecular responses, as well as their variation between different genotypes still remain unresolved and should be addressed by further experiments. In this respect, physiological investigations of siderophore production, pool sizes and fluxes as well as of corresponding siderophore/Fe(III)-chelate ratios of a diverse germplasm set might provide answers to these issues.

### A transcriptomic compendium of B73 and Mo17 at two different iron regimes

Apart from transcriptional differences a high number of polymorphisms could be detected between both inbreds, which is in concordance to previous studies [25]. Correspondingly, a striking drop in polymorphisms on the long arm of chromosome VIII was observed as previously described [20] (Figure 3, Additional file 1: Figure S5). In contrast to this study [20], a considerable number of differentially regulated transcripts has been detected (Figure 3, Additional file 1: Figure S5). The very low level of differential gene expression between B73 and Mo17 in the region on chromosome VIII described by Springer et al., (2009) results from comparative analysis by Affimetrix 17 K microarray data of seedling, embryo and endosperm tissue [26] without any variation in iron regime. In this respect, the observed strong regulation differences (Figure 3, Additional file 1: Figure S5) in the present study are likely due to the action of trans-acting factors, being polymorphic between B73 and Mo17. Moreover, this emphasizes a putative involvement of regulated transcripts in processes linked to efficient iron homeostasis or stress response in general. The relative low number of polymorphisms between both inbred are likely due to the fact that B73 and Mo17 are identical by descent for this region [20]. However, as recent studies could show that a maize diversity set displays sequence variation in the corresponding chromosome section [25,27], a selective fixation during the breeding process [28,29] or a general lack of structural variation in this region seems to be unlikely. Further investigations of this polymorphic [25] and transcriptional landscape, using a maize diversity set grown at different iron regimes, might provide extended information about genes underlying iron efficiency not only of the IBM population but of maize in general.

Concerning transcriptional differences in the iron responsive signatures of both inbreds, altogether 412 significantly regulated genes over all two-way comparisons could be identified (Figure 2, Table 1, Additional file 1: Table S3). These differences were validated by qRT-PCR for several known [5,6] as well as novel candidate genes (Table 3). Overall, a highly significant correlation (p < 0.001, R^2^ = 0.77) was observed (Figure 6), which confirms the quality of the presented transcriptional profiling.

Differentially regulated genes resulted from a combination of the most widely used analytical approaches for the analysis of RNA-Seq data, notably RPKM/FPKM based cufflinks, as well as raw count data based *edgeR* and *DeSeq* proceedings. These methods can be divided into two concepts referring to library size (*edgeR* and *DeSeq*) or distribution adjustment of read counts (*cufflinks*) [30]. Both *edgeR* and *DeSeq* rely on the hypothesis that most of the genes are not differentially expressed and propose a scaling factor for data normalization [30]. In contrast *cufflinks* uses library size and gene length normalization [17]. Beside these differences in normalization, determination of differentially expressed genes also relies on slightly different statistical tests. This in consequence results DE genes that are specific for single analysis approaches (Additional file 1: Figure S1). Nevertheless, most significant genes are conserved among the different approaches. *DeSeq* appears to be most stringent in the determination of DE genes, which might be due to the fact that several transcripts are characterized by low expression and elevated biological variation between biological replicates. These in consequence will be discarded by the *DeSeq* algorithm. In contrast, *edgeR* seems to be liberal for lowly expressed genes but compensates for this by being more conservative with strongly expressed genes [18]. Recently, *cufflinks* and the RPKM normalization, which are still widely used, were described to be ineffective in the context of differential transcriptome analysis and should be abandoned [30]. In contrast, *DeSeq* and *edgeR* normalization methods and data analysis were described to be more robust in the presence of different library sizes and widely different library compositions, both of which are typical of real RNA-seq data [30]. Finally, combination of these different, approaches, as presented in this study, offers the possibility to access significant DE genes in single approaches as well as conserved DE genes among all used approaches. In consequence, we propose the use of an experiment wide FDR significance threshold (FDR < 0.05) to unify the results of such a multi statistical transcriptome survey as a robust gene selection criterion. In this respect, the two genotype specific comparisons yielded the highest number of differentially expressed genes (Table 1). Although, the overlap between treatment and genotype comparisons was low seven out of 19 significantly regulated genes within Comparison 4 were also identified as such in Comparison 1 (Figure 2). The low number of significantly regulated genes within Mo17 in the experiment wide and the single statistical test approaches in response to the applied iron regimes (Comparison 3) provides further evidence that Mo17 is unable to efficiently respond upon low iron availability. Furthermore, the overlap detected between the genotype specific comparisons at both iron regimes might indicate that in addition to the treatment responsive genes, other factors for example such, influencing genotype specific root and shoot development and morphology might also contribute to overall stress tolerance and in consequence might also impact the iron deficiency associated chlorosis response. Whether the differential expression of the identified transcripts is causal for the observed phenotypic differences, or if it is merely a consequence of factors acting up-stream of these genes and modulating their expression needs further functional investigation. Nevertheless, principal component analysis of significant transcripts and their expression patterns allows to clearly separate genotype and treatment samples as well as differentiate a pronounced iron deficiency response for B73, which is not the case for Mo17 (Additional file 1: Figure S1C). This result further substantiates that Mo17 is unable to efficiently respond upon low iron availability.

### Differentially expressed transcripts co-localize with QTLs for iron related traits in the IBM population

A first hint, if the identified genes might not only contribute to phenotypic differences between both inbreds as result of genetic background but also to the natural variation of iron efficiency related traits within the IBM population relies in the projection of the corresponding genes onto the genetic map with corresponding QTLs (Benke et al., unpublished). Indeed, 27 genes co-localized within QTL confidence intervals, detected by Benke et al. (Figure 3, Table 2 and Additional file 1: Figure S3). The majority of these genes arose from the genotype specific comparisons (Table 2). However, it is noteworthy that two genes from the comparison of B73 grown at the 10 vs. 300 μM iron regime also mapped to QTL confidence intervals (Table 2). These candidates (GRMZM2G574782 and GRMZM2G423972) encode a putative bifunctional methylribose-1-phosphate dehydratase/enolase phosphatase E1 (*DEP1*) and a formate dehydrogenase isoform, respectively (Table 2). As both genes are important for methionine salvage pathway, a prerequisite for an efficient phytosiderophore synthesis [6,31-33], they represent excellent candidates underlying natural variation of iron efficiency within the IBM population (Table 2).

### Analysis of iron deficiency associated chlorosis by an integrative approach using transcriptome, functional and pathway information

This hypothesis is further strengthened by a GO-term enrichment and a pathway analysis that highlights the importance methionine, related processes (Figures 4 and 5, Additional file 1: Figure S4) in concordance to comparable studies in other plants [34-39]. Correspondingly, Mo17 displayed higher stress levels even at high iron regime as consequence of an inefficient iron homeostasis (Additional file 1: Figure S4). As amino-acid and carboxylic acid metabolism yield necessary backbones for phytosiderophore synthesis [5,32], an up-regulation as observed for Mo17 at both iron regimes fortifies the hypothesis of inefficient iron deficiency response for Mo17, independent of physiological iron supply. Even the compensation response of Mo17, increasing amino-acid metabolism and nucleotide as well as methionine salvage pathways, [32] together with a reduced polyamine synthesis in order to achieve a higher metabolite flux towards phytosiderophore production, as deduced from the transcriptional profile, fails to complement the inefficient iron response (Figure 5, Additional file 1: Figure S4). In contrast, B73 induces nucleotide and methionine salvage pathways only upon limiting iron supply, and does not require modulating polyamine synthesis (Figure 5, Additional file 1: Figure S4). Further evidence for this hypothesis is provided by the up-regulation of metal handling pathways and corresponding transcripts independent of iron concentration in Mo17 (Figure 5, Additional file 1: Figure S4).

### Novel candidates and old cues with new twists contribute to the iron responsive transcriptome of B73 and Mo17

A closer analysis of phytosiderophore and strategy II related pathways substantiates the picture of a severely flawed pathway in Mo17 when compared to B73. Whilst the majority of Yang cycle genes [32] as well as those being involved in phytosiderophore synthesis [5] are already up-regulated at 300 μM iron in Mo17, all of these are only induced by B73 to similar transcript levels than Mo17 upon limiting iron (Figure 5). A particularly striking fact is that Mo17 shows constitutively high expression levels for these genes at both iron regimes (Figure 5). Intriguingly, *NAS1*, which was previously thought to be one of three *NAS* isoforms in maize [21], was identified as being differentially expressed in the comparison of B73 grown at 10 versus 300 μM iron and represented by two different gene identifiers (Table 3, Additional file 1: Figure S7, S8, S9 and S10). A closer look at the corresponding chromosome region and a BLAST search manifested that two distinct regions harbored all together five *NAS1* genes (Additional file 1: Figure S6). The two detected differentially regulated isoforms map to chromosome 9 and are separated by another *NAS1* homologue that also shows differences in read coverage between B73 and Mo17. Moreover, the high sequence homology between these isoforms prohibits the correct affiliation of RNA-Seq reads and therefore the detection of truly differentially expressed isoforms. Nevertheless, the deduced read coverage in our study suggests a preferential expression of the isoforms GRMZM2G385200 and GRMZM2G034956 in B73 (Additional file 1: Figure S6). The drop in read coverage within the *NAS1* isoform GRMZM2G312481 in B73 in contrast to Mo17 points to an expression that is specific for Mo17 (Additional file 1: Figure S6). However, as the high homology of the corresponding genetic loci hampers the validation of these results by qRT-PCR, other approaches like pyro-sequencing based assays have to be applied in order to fully understand the specific spatio-temporal regulation pattern. In addition to *NAS1*, also *NAAT* and a *DEP1* homologous transcript showed transcriptional differences within both inbreds upon iron deficiency (Figure 7, Table 3 and Additional file 1: Figures S8 and S10). In contrast to Mo17, which displayed elevated expression levels independent of iron conditions for the corresponding genes, B73 induced the latter only upon iron deficiency to equal levels than Mo17 (Figure 7, Additional file 1: Figures S8 and S10). Correspondingly, we suggest that a transcription factor regulating expression of these and other downstream genes, is either impaired in its regulatory function or its own expression upon the iron deficiency signal.

Interestingly, two bHLH transcription factors, which were significantly induced in B73 upon limited iron showed elevated transcript levels at Mo17 across both iron regimes (Figure 7). These regulators show high homology to *IRO2* from rice [23,40,41] as well as *PYE* from Arabidopsis and *IRO3* from rice, respectively [22]. The gene, GRMZM2G057413 might encode the maize *IRO2* homologue, which is responsible for the induction of iron deficiency related genes, including those involved in nicotianamine synthesis [6,23]. The high levels of *IRO2* in Mo17 even at 300 μM provide an explanation for the elevated expression of genes related to methionine salvage pathway and nicotianamine synthesis [42,43]. In addition, read coverage within the corresponding gene model exceeded the current one for 20 kb downstream of the putative transcription start (Figure 7, Additional file 1: Figure S7). Surprisingly, read coverage was high in Mo17 in a region far away from the putative transcription start site but at the vicinity of another gene (GRMZM2G057506) with no mapped reads. Interestingly the rice homologue also possesses a bHLH domain (data not shown). Altogether, our data indicate that the gene model for the maize locus GRMZM2G057314 might have to be revised and furthermore that this locus might undergo alternative splicing. In addition, qRT validated higher expression levels for Mo17 even at 300 μM iron but also suggested a further significant induction at 10 μM iron for Mo17 (Figure 7). High *IRO2* levels in rice result from the action of the up-stream *iron deficiency responsive element-binding factor 1* (*IDEF1)*[42,43]. Although, a slight difference in *IDEF1* could be detected by qRT (data not shown), the expression pattern within the RNA-Seq data was not significantly different at an FDR < 0.05. Intriguingly, the second bHLH transcription factor showed as closest homologues the rice *IRO3* gene and *PYE* (Popeye) of Arabidopsis. Induction of this gene in B73 upon limiting iron and its expression profile, which is comparable to GRMZM2G057413 in Mo17 (Figure 7) indicates a contribution to efficient iron homeostasis. *AtPYE*, is known to be involved in maintaining iron homeostasis under low iron conditions and regulating root hair morphology of the strategy I plant Arabidopsis [22]. *OsIRO3*, which also shows high homology to GRMZM2G350312 has been described as an iron regulated bHLH transcription factor that plays an important role for Fe homeostasis in rice by acting as negative regulator of the Fe deficiency response [44]. The transcription profile of GRMZM2G350312, as observed in this study is identical to the one of *IRO3*in rice. In this respect, the corresponding maize gene might act analogous to *OsIRO3*. If both maize transcription factors act independent of each other or are both controlled by higher ranking factors like *IDEF1* needs further investigation. Moreover, as bHLH transcription factors often form homo- and/or hetero-dimers [45,46], a possible interaction of these genes might impact on iron deficiency associated chlorosis of both inbreds. A more essential question that has to be addressed by further experiments is, if solely internal cellular iron levels act as iron deficiency signal and trigger the *IDEF1*/*IRO2*[47] and *IRO3* like regulation networks or if other mechanisms are involved. An e-QTL study of the identified iron responsive transcription factors (GRMZM2G057413 and GRMZM2G350312) would provide first answers.

In addition, a putative oligopeptide transporter (GRMZM2G400602) could also be identified that showed an induction in its expression within the RNA-Seq experiment for both genotypes upon limiting iron conditions (Figure 7, Table 3). This regulation pattern could be validated by qRT, although induction upon 10 μM iron regime in replicate B was stronger for Mo17 than for B73 (Figure 7). One might speculate, that this transport protein, which only shows weak homology to the yellow stripe like (YSL) transporter clade [48] might be involved in the intracellular transport of phytosiderophores, their precursors or related iron complexes. A putative function in phytosiderophore export to the rhizosphere can be excluded, as the maize homologues [49] of the corresponding gene in rice *OsTOM1*[50] differ from this putative oligopetide transporter.

An intriguing finding is the strong expression difference of a putative obtusifoliol-14α demethylase (GRMZM2G096029), which undergoes a down-regulation in B73 upon limiting iron, but displays nearly no expression in Mo17 (Figure 7, Table 3). As this gene is involved in sterol biosynthesis, and as it has been previously described that the relative abundance of Δ^5^-sterols correlates with aluminum tolerance in rice [51], a potential consequence of the extremely low transcript expression in Mo17 could be an altered sterol composition of root membranes. As low sterol abundance seems to correlate with higher membrane permeability and an accumulation of metal ions [51], higher sterol abundance in B73 root tissue might improve its iron efficiency by reducing undesired metal accumulation and toxicity.

In addition, the regulation pattern of the gene GRMZM2G106980 encoding a putative BURP-domain could be due to developmental processes impacted by the stress response as described elsewhere [24]. Moreover, expression of a *ClpC1* homologous chaperone gene could also be validated by qRT-PCR (Additional file 1: Figure S8). *AtClpC1*, has been identified as being responsible for the Arabidopsis mutant phenotype irm1, showing typical Fe-deficiency chlorosis [52]. In this respect, ClpC1 is involved in leaf iron homeostasis, presumably via chloroplast translocation of some nuclear-encoded proteins which function in Fe transport. The homologous gene in maize might fulfill similar functions. The high transcript levels of two cytochrome P450 genes, involved in polyphenol synthesis, in Mo17 at both iron regimes points to an iron deficiency compensation response (Additional file 1: Figures S7 and S10). In this respect, a flawed iron deficiency chlorosis response in Mo17 might lead at both iron conditions to increased production of phenols that, after being excreted to the rhizosphere, solubilize Fe(III) from the soil [6]. Whether this Strategy I related mechanism supports iron assimilation by phytosiderophores [6] remains theoretical. Another, explanation would be a general stress response of Mo17, which in consequence results in elevated phenol production [53].

## Conclusion

Over 400 significantly regulated transcripts at two iron regimes within both inbreds were identified that besides novel candidate genes included known iron responsive loci. The integration of QTL information and transcriptome data emphasize a contribution of the above mentioned genes to natural variation of the investigated traits. Further analysis of the proposed candidate genes and pathways in a diverse germplasm set will extend our understanding about the impact of the latter to natural trait variation in maize. The presented data is a valuable resource for researcher investigating iron deficiency response in graminaceous and non-graminaceous plants and represents a vantage point for the generation of molecular markers in order to improve iron deficiency chlorosis resistance in maize.

## Methods

### Plant material, hydroponic growth and root tissue sampling

Seeds of the maize genotypes B73 and Mo17 were obtained from the Maize Genetics Cooperation Stock Center (http://maizecoop.cropsci.uiuc.edu/). Maize seeds were sterilized with a 3% NaClO solution for three minutes and then treated with 60°C hot water for another five minutes. Seeds for each genotype were germinated in Petri dishes (Greiner Bio-One GmbH, Frickenhausen, Germany) between two filter paper sheets moistened with saturated CaSO_4_ solution in the dark at room temperature. After six days of imbibition, germinated seeds were transferred to a continuously aerated nutrient solution with nutrient concentrations as described [54] and supplied with 100 μM Fe(III)-EDTA for the following seven days. Afterwards, half of the pots representing a genotype group were shifted to either limiting [10 μM Fe(III)-EDTA] or non-limiting [300 μM Fe(III)-EDTA] iron conditions. The nutrient solution was exchanged every third day. Four plants of each genotype were grown in one 5 liter pot until the 28^th^ day in a growth chamber at a relative humidity of 60%, light intensity of 170 μmol m^-2^⋅s^-1^ in the leaf canopy, and a day-night temperature regime of 16 h/24°C and 8 h/22°C, respectively. In order to correct for the biological variation, the experiment was carried out two times each with three pots harbouring four plants of a given genotype at the mentioned iron conditions.

### Phenotypic evaluation

Phenotypic evaluation of chlorosis symptoms of the 5^th^ and the 6^th^ leaf, measured as SPAD units (SPAD5, 6), was carried out 25 days after germination for individual plants with a SPAD meter (Konica Minolta SPAD 502, Langenhagen, Germany). All other traits analysed in this study were recorded at harvest (28 days after germination). Root length (RL) and root weight (RW) were measured for all plants in one pot as one sample and root tissue was immediately frozen in liquid nitrogen at harvest for subsequent RNA-Seq analysis. Shoot length (SL) was measured independently for each plant and shoot dry weight (SDW) was evaluated after drying the shoot material for 7 days at 70°C. Dried shoot material of all the plants from one pot was pooled to one sample of each of the two experimental replications. Afterwards, shoot samples were ground and measured for Fe concentration using inductively coupled plasma optical emission spectrometry ICP-MS (Elan 6000, Perkin Elmer Sciex, Rodgau, Germany).

### RNA isolation, library construction and RNA-Seq run on Illumina HiSeq2000

After harvest and phenotypic evaluation, frozen root tissue for all plants in one pot was used for RNA isolation. Root tissue samples from three pots each with four plants of a specific genotype at a given iron regime were pooled for each biological replicate (two independent experiments) and ground in a mortar under liquid nitrogen. About 200 mg of tissue from both biological replicates was subjected to total RNA isolation using the Qiagen RNAeasy Plant Mini Kit (QIAGEN, Hilden, Germany). RNA concentration was measured by the QuBit broad range RNA assay kit (Life Technologies GmbH, Darmstadt; Germany) and the QuBit fluorimeter (Life Technologies GmbH, Darmstadt; Germany). Integrity of isolated total RNA and possible DNA contamination was checked on 1.2% agarose gels. DNA contamination was subsequently eliminated, using the Ambion DNA-free TM kit, (Life Technologies GmbH, Darmstadt; Germany). Successful DNAse treatment was monitored on the Agilent Bioanalyser (Agilent Technologies, Böblingen; Germany). Further processing of RNA included an rRNA depletion step using the RiboMinusTM Plant Kit (Life Technologies GmbH, Darmstadt; Germany) and the monitoring of depleted RNA using the Agilent Bioanalyser pico Chip (Agilent Technologies, Böblingen; Germany). RNA-seq libraries were prepared from depleted samples according to the recommendations of the supplier (TruSeq RNA sample preparation v2 guide; Illumina). Libraries were quantified by fluorometry, immobilized and processed onto a flow cell with a cBot (Illumina), followed by sequencing-by-synthesis with TruSeq v3 chemistry on a HiSeq2000 system. Raw sequencing data was processed with Illumina software *CASAVA* (ver. 1.8.2). Raw data files can be accessed as FASTQ files via the Short Read Archive at NCBI (http://www.ncbi.nlm.nih.gov/sra) under the BioProject ID PRJNA187035.

### Transcriptomic data analysis

Raw RNA-Seq reads were analysed, using the statistical software *R* and reads having a Phred score of equal to, or less than 20 in more than 30 percent of the cycles were removed [55]. After creating an indexed reference with *Bowtie2 v.2.0.0-beta 6*[56,57] and the current maize sequence (ZmB73_RefGen_v2, http://ftp.maizesequence.org) as well as the reference annotation file (ZmB73_5a, http://ftp.maizesequence.org) [58], high quality reads were aligned to the reference using *TopHat v.2.0.3* with standard settings [17,59]. The resulting BAM files were sorted and indexed with *SAMtools v.1.4*[60] as well as processed to SAM files. The further transcript assembly and calling of differentially expressed transcripts was carried out, using four different procedures. For transcript assembly by *cufflinks v.2.0.2*, sorted SAM files were used either by providing a annotation file for reference annotation based transcript assembly (RABT) in order to rapidly identify novel transcripts [61] or without any annotation reference as previously described [17]. The further analysis was performed using *cufflinks v.2.0.2* with default settings [17]. In this respect, transcript abundances is calculated in Fragments Per Kilobase of exon per Million fragments mapped (FPKM), which is analagous to Reads Per Kilobase of exon per Million fragments mapped (RPKM) when analysing single-read runs. Subsequent proceeding of Cufflinks output with Cuffcompare, Cuffmerge and Cuffdiff with default settings resulted in determination of differentially expressed transcripts after data normalization in order to correct for differences in both library sizes and gene length. Furthermore, sorted and indexed BAM files were processed with the statistical software *R v.2.15.1*[62] and the *R* package *EasyRNASeq* by providing an annotation object created by the *R* packages *EasyRNASeq*, *biomaRt*, *GenomicRanges* and *GenomicFeatures*[63]. Correspondingly, calling the *easyRNASeq()* function of the *R* package *EasyRNASeq* resulted in tables with raw count data that can be further analysed by the packages *DeSeq* or *edgeR* using as output format option within the *easyRNASeq()* function *“DeSeq”* or *“edgeR”*, respectively. The derived count tables for the *R* packages *DeSeq*[18] and *edgeR*[19] were computed per gene models. Identification of differentially expressed transcripts was carried out as previously described [18,19,63,64]. In this respect, normalisation of count data is achieved using the *DeSeq* functions *estimateSizeFactors()* and *sizeFactors()* in order to determine a normalization factor as well as following the trimmed Mean of *M*-values (TMM) approach using the edgeR function *calcNormFactors()*.

Differentially expressed transcripts were called for specific two-way comparisons for each analysis pipeline (Additional file 2). In this respect, Mo17 transcript expression was compared to B73 at 300 μM and at 10 μM iron supply. In addition, Mo17 as well as B73 transcript expression at 10 μM iron was compared to that present at 300 μM. In order to standardize selection of differentially expressed transcripts the threshold of significance was set to an experiment wide false discovery rate (FDR) [65] lower than 0.05 for all tests. Only transcripts significant at this experiment wide FDR throughout all four statistical approaches for corresponding two-way comparisons were further analysed.

Genetic map and QTL intervals (Benke et al., unpublished) were used for the projection of the physical positions of differentially expressed and significant genes onto the corresponding map. Information on physical positions, sequences accession numbers and molecular characteristics were retrieved from corresponding web resources [58,66]. Transcripts showing expression values in all four statistical approaches were projected with their experiment wide median log_2_FC for specific two-way comparisons onto the physical map for each chromosome.

### GO-term enrichment and pathway analysis

Based on version 5b of the filtered gene set of the maize genome, sequences of significantly regulated transcripts within all four two-way comparisons (FDR < 0.05) throughout all four statistical approaches were determined. An unique protein identifier from the UniProt Knowledgebase data set [67] was identified for each transcript using BLAST software [68] with an expectation cutoff value of 1e^-10^. Based on the unique protein identifier, gene ontology (GO) terms were assigned. Singular GO term enrichment analysis [69] was carried out using the maize transcript sequence file version 5a as reference. To determine significantly enriched GO terms between the significantly regulated genes within the RNA-Seq approach and the reference a hypergeometric test with a level of false discovery rate lower than 0.05 was applied [70]. GO-terms showing a fold enrichment within the last quartile of all values for each category are plotted in Figure 4.

Visualisation of transcript expression differences within specific pathways was carried out, using *MapMan v.3.5.1R2*[71]. Genes that were identified as differentially expressed within two-way comparisons were used as input together with their median log_2_FC across all statistical tests. In order to use the mapping file provided by the *MapMan* homepage (Zm_GENOME_RELEASE_09, http://mapman.gabipd.org), gene identifiers were converted into transcript identifiers by adding the extension “_T01”. Moreover, custom iron homeostasis pathway files (Additional files 3 and 4) were used.

### Variant calling and data adjustment

In order to detect polymorphisms between B73 and Mo17, *TopHat v.2.0.3* was used to generate BAM files out of all FASTQ files of a given genotype across both iron treatments and the two replications. By using *SAMtools v.1.4* BAM files were sorted and indexed. Variant calling was conducted, using the *mpileup* function with default settings together with the current maize sequence (ZmB73_RefGen_v2) as reference. The *SAMtools* output was further processed with *SAMtools*/*BCFtools v. v.1.4*[60] into VCF files [72]. VCF files were filtered using *R*. Only polymorphisms having an average read depth above 10 and being clearly called as homozygous (a single PL value of 0) were kept. The number of polymorphisms (SNPs and INDELs) was computed for arbitrary bins (size = 4 MBps) for each chromosome and plotted along the physical coordinates.

### RT-PCR and selection of candidate genes

Candidate gene selection criteria were the following: i) pseudogenes, transposable elements and transcripts giving rise to very small peptides (< 50AA) were omitted from validation by RT-PCR, ii) all genes significantly regulated in the comparison of B73 grown at 300 and 10 μM iron (Comparison 4 – 11 genes), except *Nas1* and *Fdh*, which were represented by multiple isoform as well as the significantly regulated genes of the comparsion of Mo17 grown at 300 and 10 μM iron (Comparison 3 – 3genes) were included in the RT-PCR validation, iii) together with genes significant at the comparisons of both genotypes at 300 and 10 μM iron (Comparison 1 and 2 – 6 genes), respectively and ranging with their experiment wide median P-values within the upper quartile of the corresponding distribution. In addition, 20 known iron responsive genes were also integrated in the validation approach (Additional file 1: Table S4). Total RNA for RT-PCR analysis was extracted from B73 and Mo17 root tissue as described above. After DNAse treatment (Ambion DNA-free, Invitrogen) cDNA synthesis was carried out using Reverse Transcriptase (SuperScript VILO, Life Technologies GmbH, Darmstadt; Germany) and random hexamer primers. Expression analysis by RT-PCR was conducted according to manufacturer’s instructions (DyNAmo ColorFlash SYBR Green qPCR Kit, Biozym Scientific GmbH, Hessisch Oldendorf; Germany) using Actin as an internal control to normalize transcript abundance. Corresponding primers and PCR conditions for candidate gene amplification are given in Additional file 1: Table S4.

## Competing interests

The authors declare that they have no competing interests.

## Authors’ contributions

CU, AB and JM carried out the hydroponic growth of maize genotypes, tissue sampling, phenotypic evaluation and all molecular laboratory procedures. CU designed and evaluated the RNA-Seq experiment and conducted raw-data analysis. CU and AB carried out further computational analysis of transcriptome data. CU and BS conceived of the study and drafted the manuscript. BH and RR conceived of the study and provided “next-generation sequencing” infrastructure. BH carried out the Illumina HiSeq run. All authors read and approved the manuscript.

## Supplementary Material

Additional file 1 Supplemental Figures, tables and corresponding legends. Click here for file

Additional file 2 Regulated transcripts, significant in single data analysis pipelines. Click here for file

Additional file 3 **MapMan Pathway file.** JPG file for siderophore and Fe homeostasis related transcripts adopted from *Benke et al.*[5] without transcript bins. Click here for file

Additional file 4 **MapMan Mapping File.** Transcript mapping file. Click here for file
