# Supplementary material for: Ups and downs of a transcriptional landscape shape iron deficiency associated chlorosis of the maize inbreds B73 and Mo17
Source: BMC Plant Biol. 2013 Dec 13;13:213. doi: 10.1186/1471-2229-13-213 (PMC3881016; doi:10.1186/1471-2229-13-213)
Supplement: Additional file 1 — Supplemental Figures, tables and corresponding legends. [file 1471-2229-13-213-S1.pdf]

Supplemental Information: Ups and downs of a transcriptional landscape shape iron deficiency associated chlorosis of the maize inbreds B73 and Mo17

Claude Urbany[1], Andreas Benke[1], Johanna Marsian[2], Bruno Huettel[3], Richard Reinhardt[3], Benjamin Stich[1*]

[1]Max Planck Institute for Plant Breeding Research, Quantitative Crop Genetics, 50829 Cologne Germany

[2]John Innes Centre, Department of Biological Chemistry, Norwich NR4 7UH, United Kingdom

[3]Max Planck Genome Centre Cologne, 50829 Cologne Germany

Email addresses:

CU: urbany@mpipz.mpg.de

AB: benke@mpipz.mpg.de

JM: johanna.marsian@jic.ac.uk

BH: huettel@mpipz.mpg.de

RR: reinhardt@mpipz.mpg.de

BS: stich@mpipz.mpg.de

* Corresponding author

Benjamin Stich

Max Planck Institute for Plant Breeding Research

Quantitative Crop Genetics

Carl-von-Linné-Weg 10

50829 Cologne, Germany

Phone: ++49 221 5062-401

Fax: ++49 221 5062-413

Running Title: Investigation of variable iron deficiency associated chlorosis

Supplemental Figures

Supplemental Figure 1 Ratios of phenotypic differences of the maize inbred B73 and Mo17 at 300 and 10µM iron regime as well as principal component analysis (PCA) of phenotypic and transcriptional differences. A) The ratios of the observed trait values were calculated and plotted as percent of variation for B73 versus Mo17 samples at 300µM iron (blue), B73 versus Mo17 samples at 10µM iron (green), Mo17 samples at low (10µM) versus sufficient (300µM) iron in yellow and B73 samples at low (10µM) versus sufficient (300µM) iron in purple. B) PCA of phenotypic data for both inbreds at 10 and 300µM iron. Phenotypic data for all replicates of corresponding samples was used for determination of PCAs. Trait values are plotted as loadings and genotypes at specific treatment conditions as samples (B73 plants at 300µM in dark green, B73 plants at 10µM in light green, Mo17 plants at 300µM in red, Mo17 plants at 10µM in orange). The percent of variance explained by the corresponding principal component is indicated. C) PCA of expression data for the set of significantly regulated transcripts at an experiment wide FDR < 0.05. Transcriptome data (FPKMs and count information) for corresponding transcripts amongst samples was used for determination of PCAs. Transcript values are plotted as loadings and genotypes at specific treatment conditions as samples (B73 plants at 300µM in dark green, B73 plants at 10µM in light green, Mo17 plants at 300µM in red, Mo17 plants at 10µM in orange). The percent of variance explained by the corresponding principal component is indicated.

Supplemental Figure 2 Venn diagrams of DE-Genes for specific statistical approaches (A-D) and for each two-way comparison using all four approaches (E-H). The corresponding number of DE genes is indicated for each space of Venn diagrams. The cufflinks based pipeline for identification of DE genes (A) shows Comparison 1 in blue, Comparison 2 in green, Comparison 3 in orange and Comparison 4 in purple. Furthermore, the number of DE genes within specific comparisons as well as their intersection is displayed for cufflinks by RABT (B), DeSeq (C) and edgeR (D). For the determination of truly DE genes (detected as differentially expressed across all four statistical approaches, namely cufflinks = dark orange, cufflinks by RABT = pink, DeSeq = cyan, and edgeR= light green) is determined for each two-way comparison (Comparison 1 = E; Comparison 2 = F, Comparison 3 = G, Comparison 4 = H). Only DE genes in the intersection of all four approaches are further analyzed.

Supplemental Figure 3 Projection of significantly regulated genes at all four statistical approaches (experiment wide FDR < 0.05) across all four two-way comparisons onto the genetic map with QTL confidence intervals for iron efficiency related traits (Benke et al., unpublished). QTL confidence intervals are indicated for corresponding traits (Fe = iron content in the shoot; SP5 = SPAD values of the 5th leaf; SP5 = SPAD values of the 6^{h} leaf, RL = root length; RW = root weight; SL = shoot length; SDW = shoot dry weight) as orange boxes for the 10µM iron regime and in dark green for the 300µM iron regime as identified by Benke et al., unpublished. Genetic distances are given in cM and were retrieved from marker information available at www.maizegdb.org for the IBM population. For projection of differentially regulated genes within specific two-way comparisons (Table 1) the available physical transcription start site positions (www.maizegdb.org) were converted into putative genetic positions by interference from adjacent marker positions. DE genes for each comparison (Comparison 1 = blue; Comparison 2 = green, Comparison 3 = orange, Comparison 4 = purple) were projected onto the genetic map for the 10 maize chromosomes.

Supplemental Figure 4 Pathway analysis of significantly regulated genes for each two-way comparison using MapMan. Median log2FC across all statistical approaches are depicted by transcript bins, using the MapMan mapping file (Zm_GENOME_RELEASE_09, http://mapman.gabipd.org) and the provided pathway files. Shown is the overall overview pathway representation and the regulation of differentially expressed genes (FDR < 0.05) within each Comparison (Comparison 1 = A; Comparison 2 = B, Comparison 3 = C, Comparison 4 = D) as color-code (red = lower expression in condition 1 vs. 2; green = higher expression in condition 1 vs. 2). Pathway 1 = Photosynthesis; Pathway 2 = major CHO Metabolism; Pathway 3 = minor CHO Metabolism; Pathway 4 = glycolysis; Pathway 5 = fermentation; Pathway 6 = gluconeogenesis/glyoxylate cycle; Pathway 7 = Oxydative pentose phosphate cycle; Pathway 8 = Tricarboxylic acid cycle; Pathway 9 = mitochondrial electron transport/ ATP synthesis; Pathway 10 = cell wall; Pathway 11 = lipid metabolism; Pathway 12 = nitrogen metabolism; Pathway 13 = amino acid metabolism; Pathway 14 = sulfur assimilation; Pathway 15 = metal handling; Pathway 16 = secondary metabolism; Pathway 17 = hormone metabolism; Pathway 18 = Co-factor and vitamin metabolism; Pathway 19 = tetrapyrole synthesis; Pathway 20 = stress; Pathway 21 = redox regulation; Pathway 22 = polyamine metabolism; Pathway 23 = nucleotide metabolism; Pathway 24 = biodegradation of xenobiotics; Pathway 25 = C1 metabolism; Pathway 26 = miscellaneous; Pathway 27 = RNA; Pathway 28 = DNA; Pathway 29 = Protein; Pathway 30 = signalling; Pathway 31 =

cell; Pathway 32 = micro RNA, natural antisense etc.; Pathway 33 = ; Pathway 34 = transport; Pathway 35 = not assigened; Pathway 36 = C4 Photosynthesis.

Supplemental Figure 5 Polymorphic and transcriptional landscape of the maize inbreds B73 and Mo17 in response to iron availability. The polymorphic and transcriptional landscape is displayed for each maize chromosome (Chr.). The number of polymorphisms (SNPs and INDELs) is given for artificial bins of a size of 4Mbps. The median log2FC for transcripts detected across all statistical approaches is shown for specific two-way comparisons (Comp. 1 = blue; Comp. 2 = green; Comp. 3 = orange; Comp. 4 = purple) at the relative physical position, represented by the transcriptional start site. In the case of absent/present expression data, log2FC of $\pm\infty$ were exchanged by ±20 (the highest log2FC across the experiment). QTLs confidence intervals for iron content in the shoot (red) and SPAD values of the 5th (turquoise) and the 6th leaf (light cyan) are indicated at their interpolated physical position for the 10 (Fe10) and 300µM iron (Fe300) regime.

Supplemental Figure 6 Schematic representation of *NAS1* gene clusters on chromosome 9 and corresponding RNA-Seq read coverage. Displayed are two clusters of NAS1 isoforms on maize chromosome 9 at the physical position 135,298,525 – 135,308,218 (A) and at position 135,438,861 – 135,798,318 (B). The un-normalized coverage tracks of corresponding *NAS1* gene models within each cluster is displayed for B73 at 300µM iron (dark green), B73 at 10µM iron (light green), Mo17 at 300µM iron (red), and Mo17 at 10µM iron (orange). The black bar represents the relative physical distances within each cluster.

Supplemental Figure 7-10 RNA-Seq read coverage of specific candidate genes and validation of the expression pattern by qRT-PCR. Displayed are the un-normalized coverage tracks of candidate transcripts (Table 3) on the left hand side for B73 at 300µM iron (dark green), B73 at 10µM iron (light green), Mo17 at 300µM iron (red), and Mo17 at 10µM iron (orange) with the corresponding gene model at the bottom. If not indicated, the black bar represents 100bps. In addition, the strand orientation is displayed (+ = sense strand; - = antisense strand). On the right hand side, the expression pattern as detected by RNA-Seq for all biological replicates is displayed as fold change (FC) relative to *Actin1* (GRMZM2G126010) expression. Validation of the expression pattern by RT-PCR within each biological replicate is shown as FC relative to *Actin1* expression with standard

deviations. For genes with missing validation by qRT-PCR no expression pattern as detected by RNA-Sea is displayed.

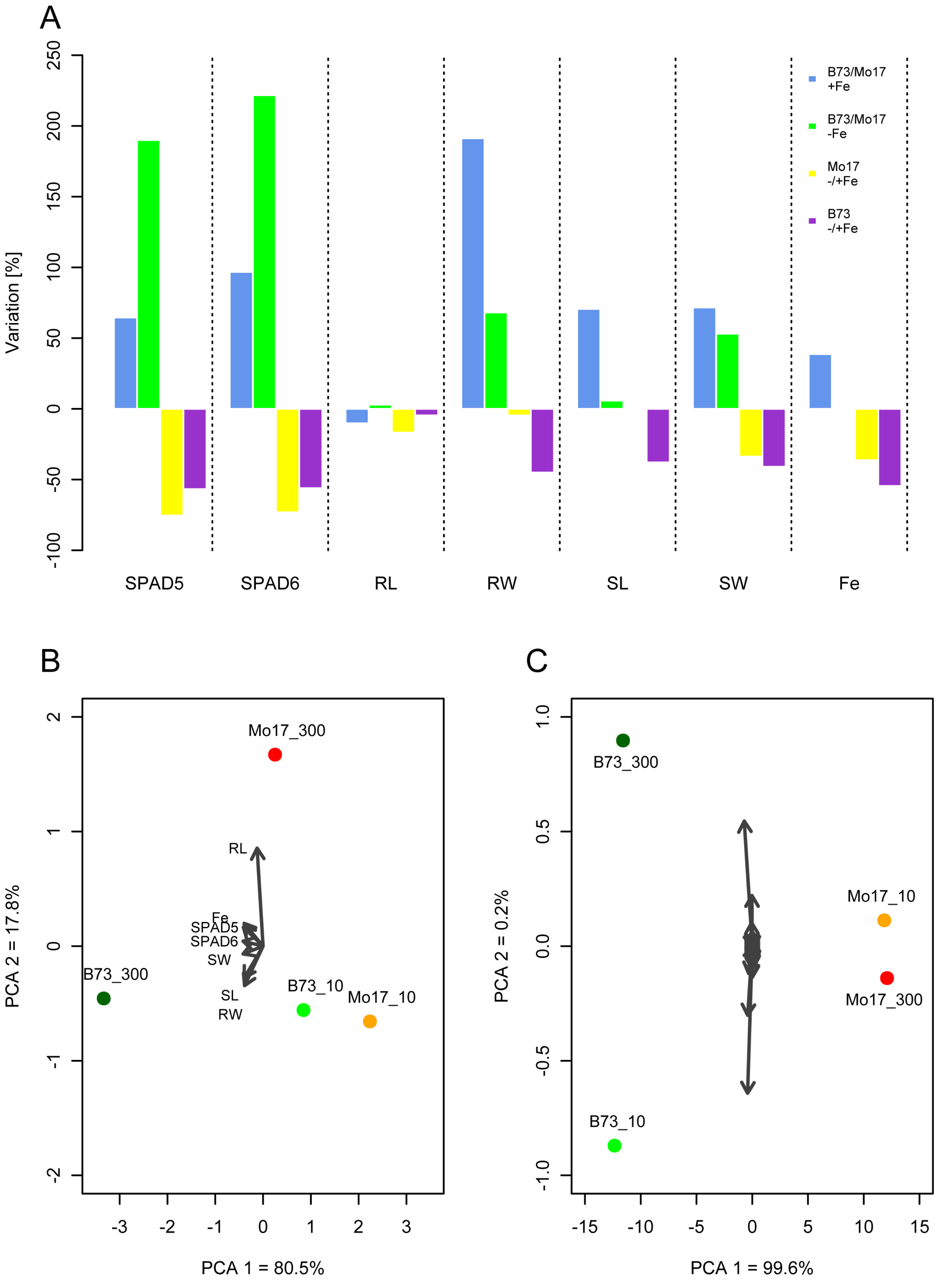

Supplemental Figure 1

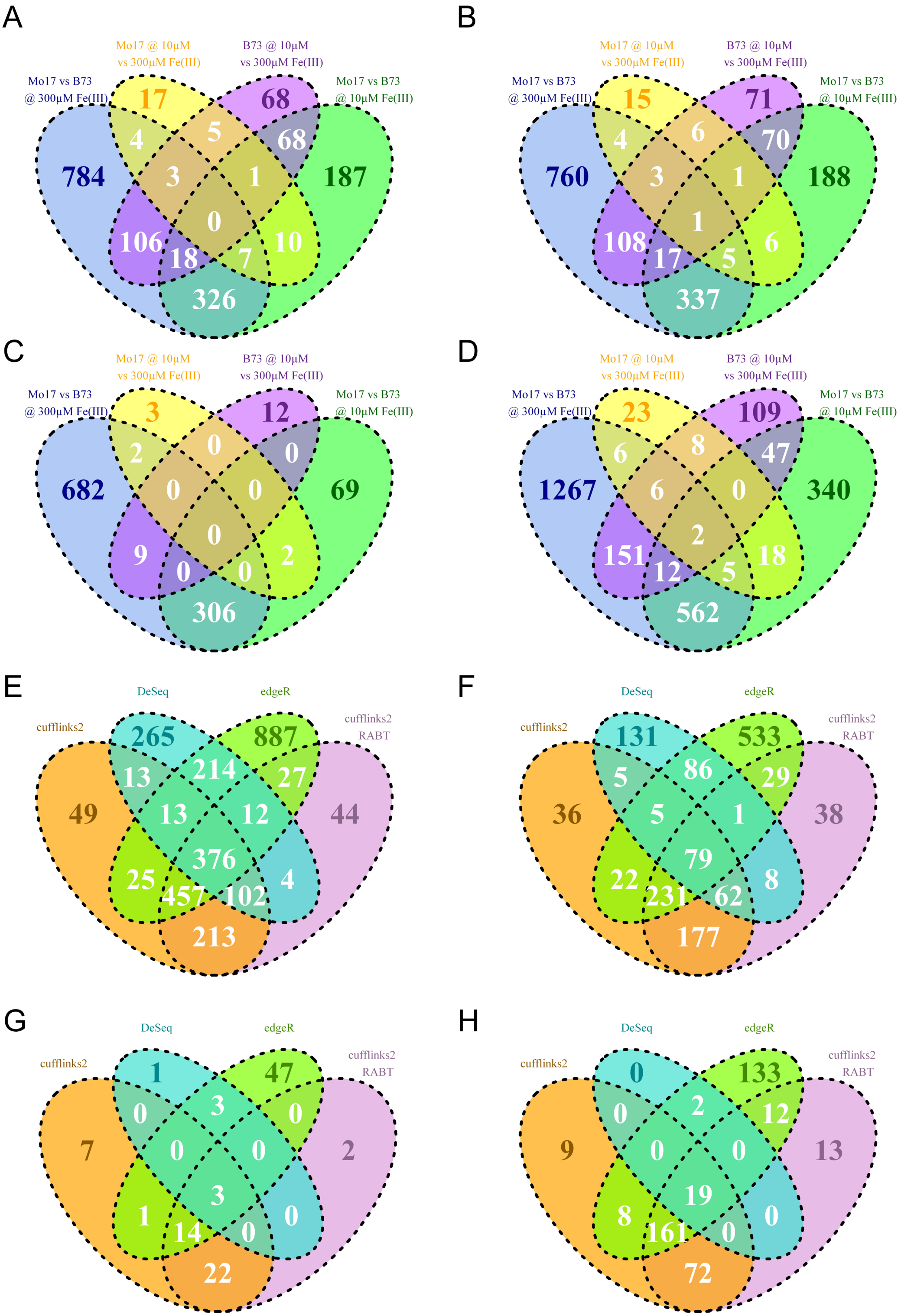

Supplemental Figure 2

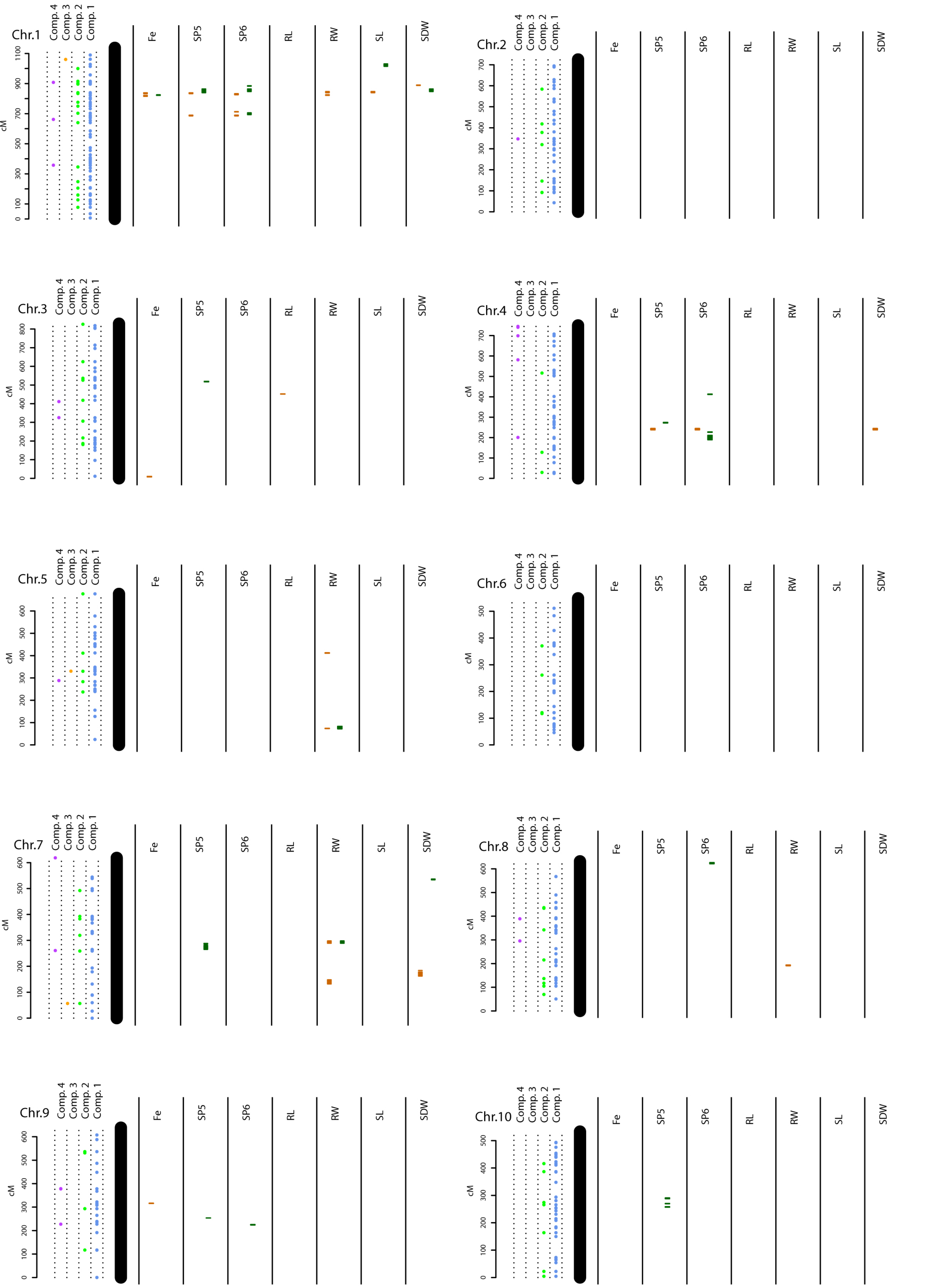

Supplemental Figure 3

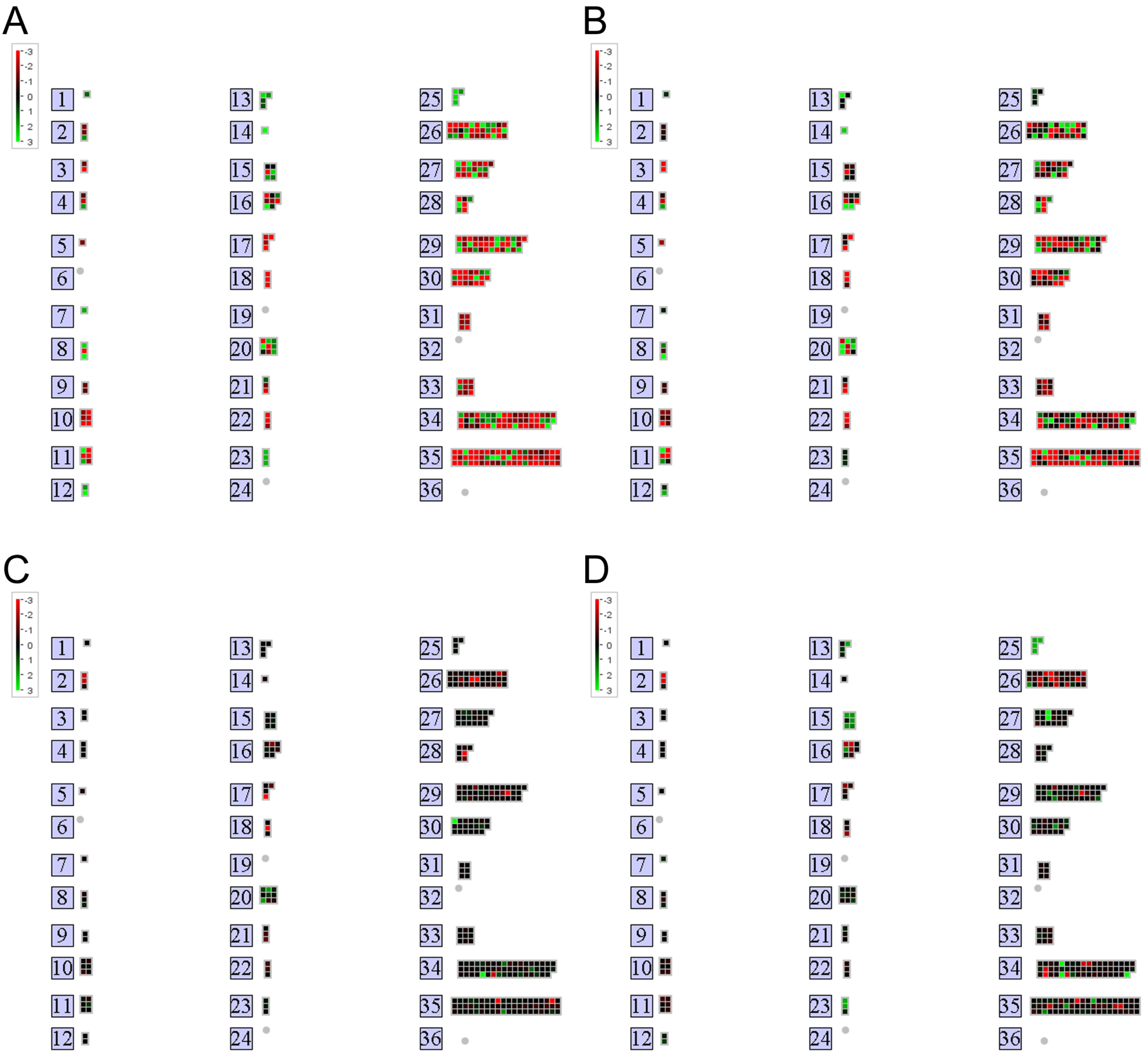

Supplemental Figure 4

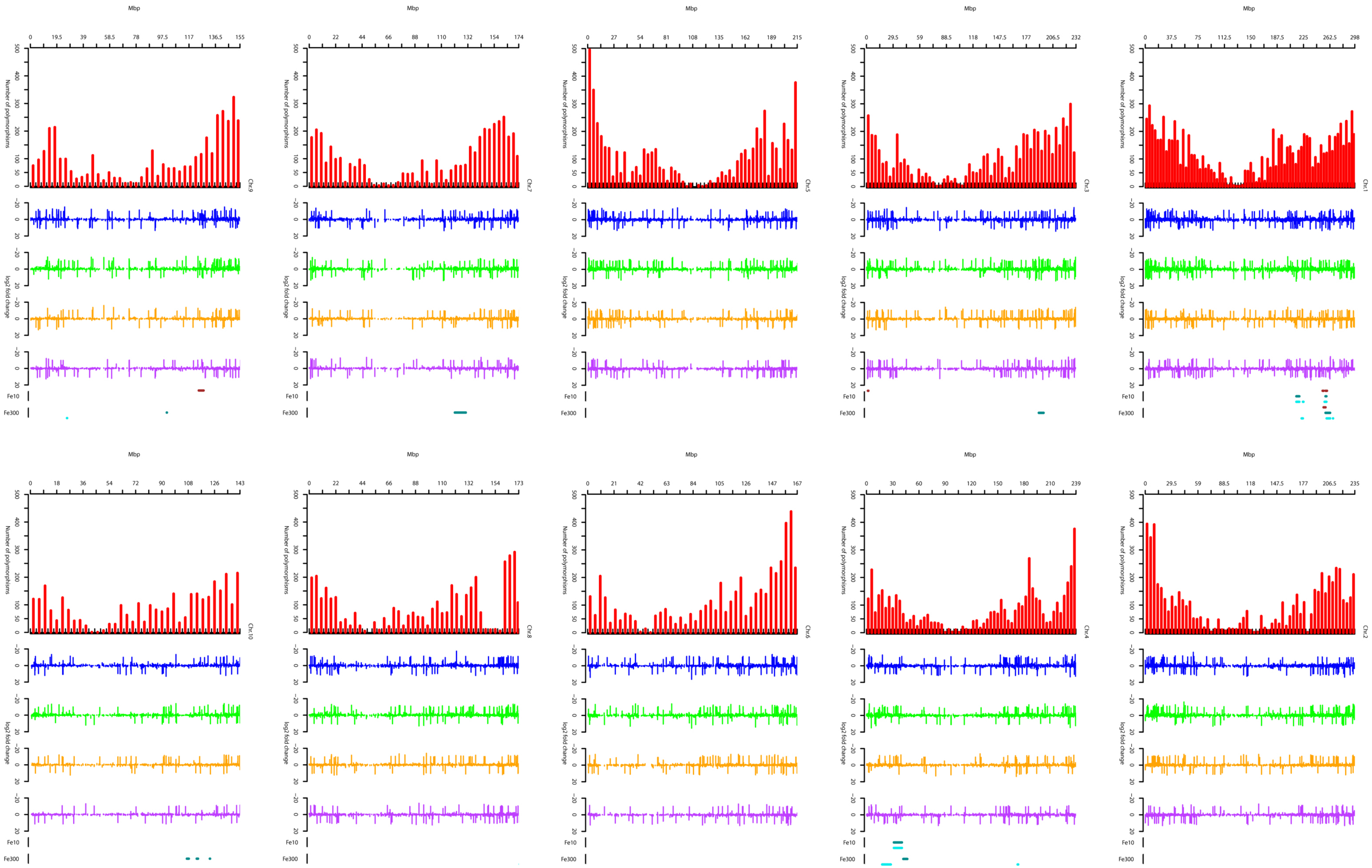

Supplemental Figure 5

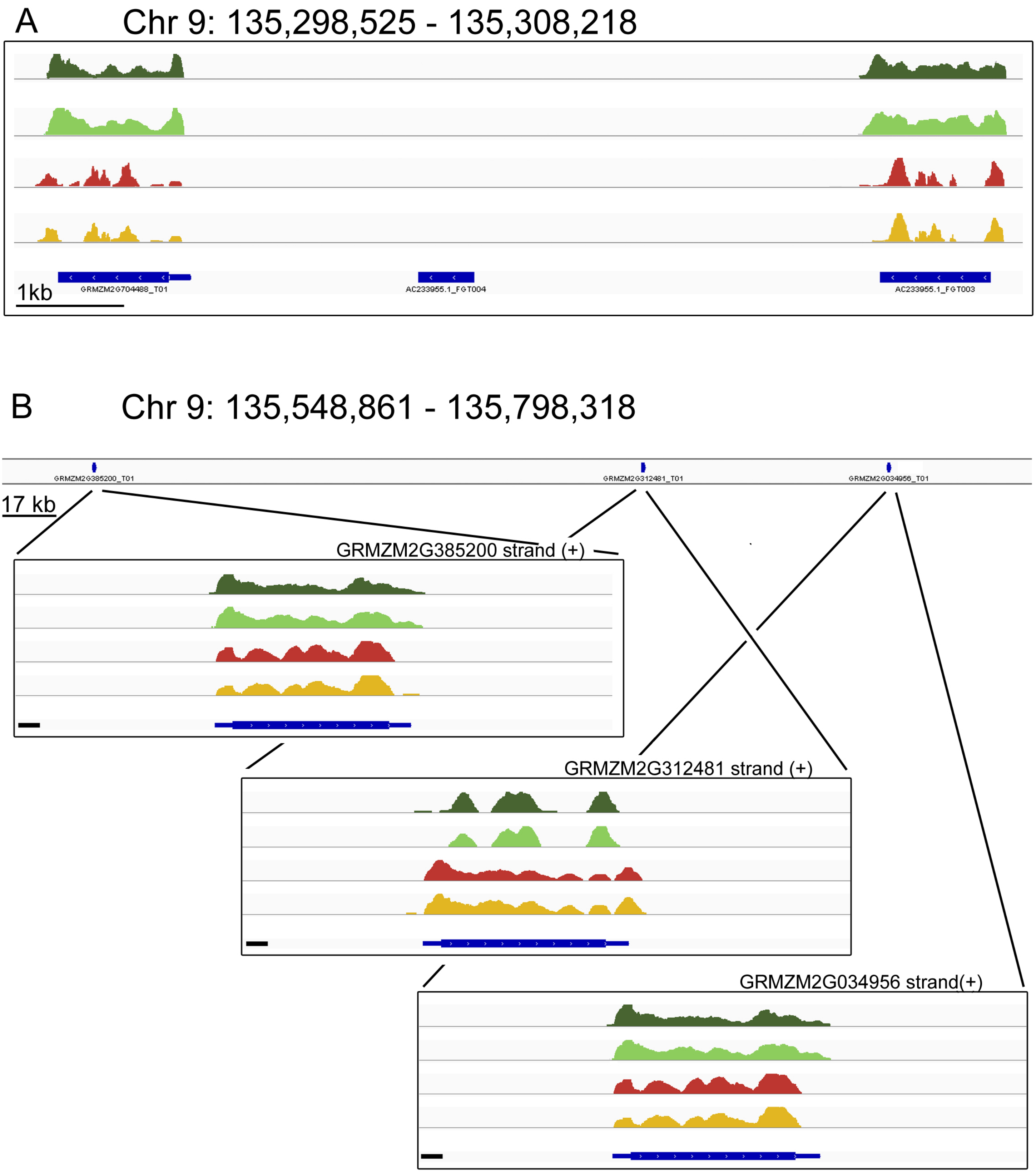

Supplemental Figure 6

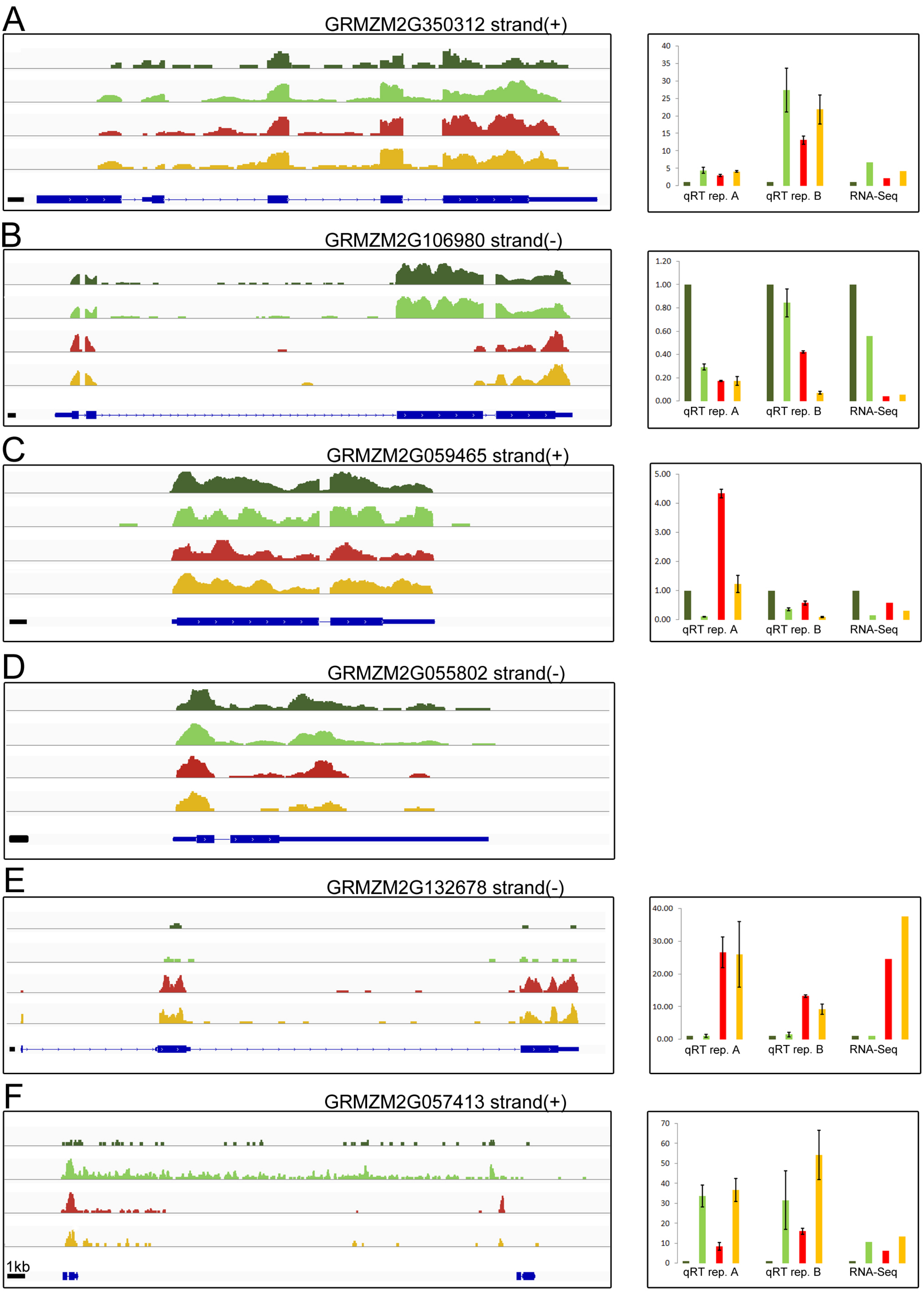

Supplemental Figure 7

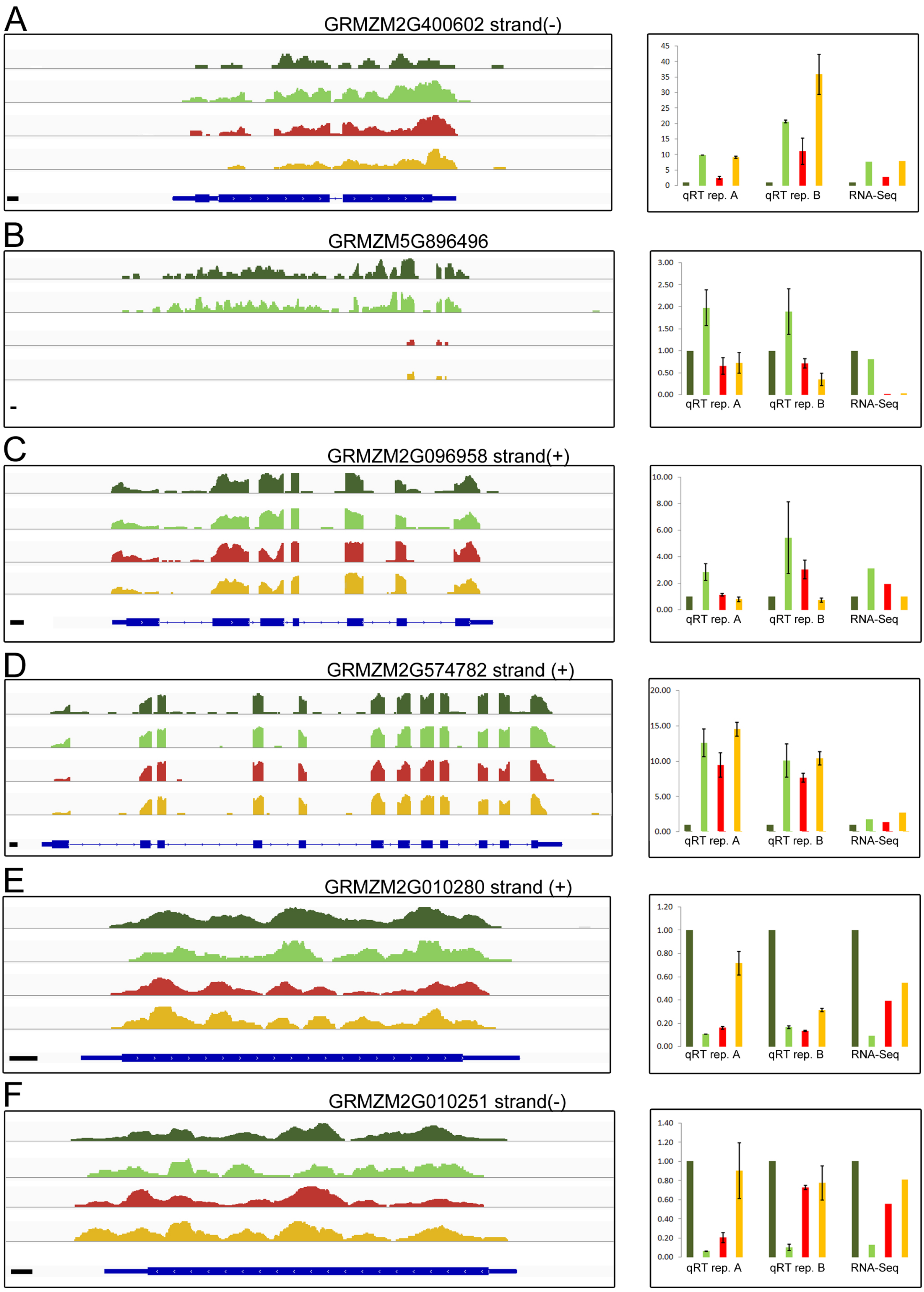

Supplemental Figure 8

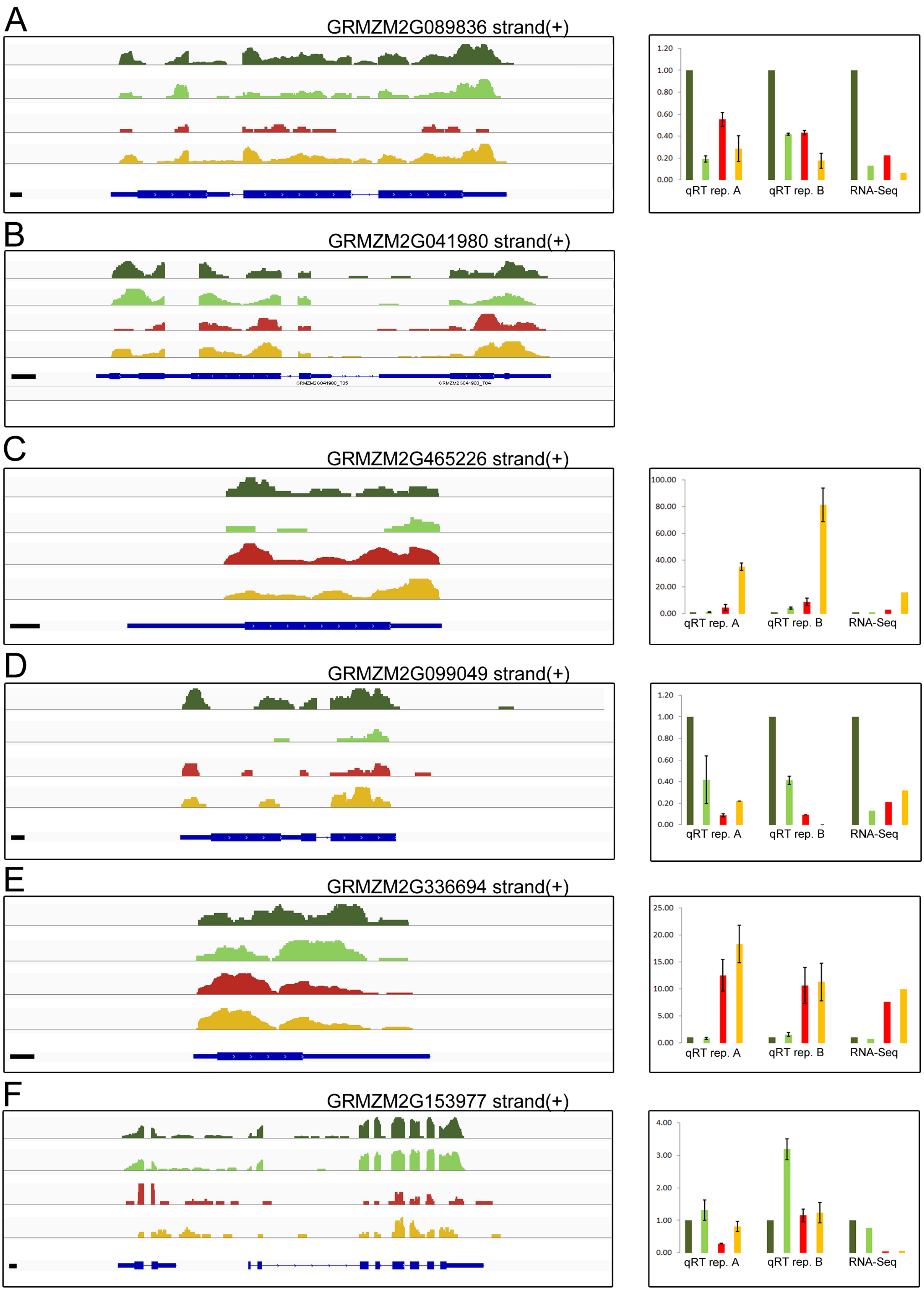

Supplemental Figure 9

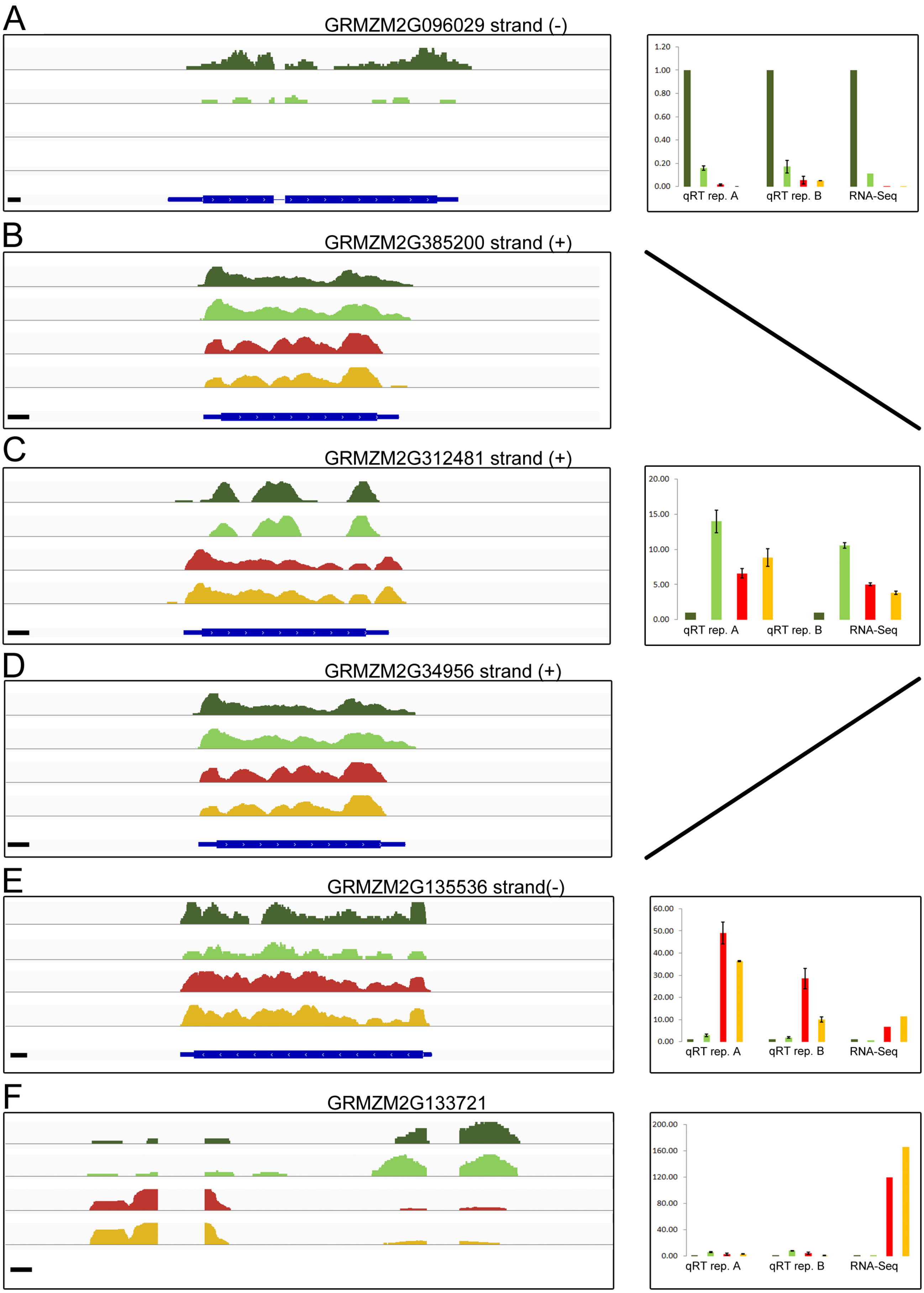

Supplemental Figure 10

Supplemental Tables

Supplemental Table 1 General read information for transcriptome approach.

sample	Total reads	Reads after QC[1]
B73 @ 10µM a	14,873,769	8,898,402
B73 @ 10µM b	13,554,414	8,330,572
B73 @ 300µM a	11,716,557	7,385,882
B73 @ 300µM b	13,001,317	7,592,845
Mo17 @ 10µM a	12,532,106	7,617,802
Mo17 @ 10µM b	8,937,296	5,544,901
Mo17 @ 300µM a	12,002,202	7,303,865
Mo17 @ 300µM b	15,063,187	8,897,259
Median[6]	12,766,712	7,605,324

[1] cf Material and Methods section for quality control (QC) filter criteria

Supplemental Table 2 GO Term enrichment analysis

GO term	Ontology[1]	Description	Fold enrichment versus Reference [2]
GO:0044464	C	cell part	2.08
GO:0005623	C	cell	2.08
GO:0005622	C	intracellular	2.14
GO:0044424	C	intracellular part	2.51
GO:0043229	C	intracellular organelle	2.64
GO:0043226	C	organelle	2.64
GO:0016020	C	membrane	2.83
GO:0043227	C	membrane-bounded organelle	3.83
GO:0043231	C	intracellular membrane-bounded organelle	3.86
GO:0005737	C	cytoplasm	4.36
GO:0005634	C	nucleus	2.31
GO:0044425	C	membrane part	4.06
GO:0044444	C	cytoplasmic part	4.55
GO:0031224	C	intrinsic to membrane	4.85
GO:0016021	C	integral to membrane	4.77
GO:0044422	C	organelle part	3.76
GO:0044446	C	intracellular organelle part	3.76
GO:0005576	C	extracellular region	5.15
GO:0031090	C	organelle membrane	7.63
GO:0031975	C	envelope	3.96
GO:0005739	C	mitochondrion	7.14
GO:0031967	C	organelle envelope	4.59
GO:0044428	C	nuclear part	7.46
GO:0005783	C	endoplasmic reticulum	5.50
GO:0030312	C	external encapsulating structure	8.66
GO:0031974	C	membrane-enclosed lumen	5.91
GO:0012505	C	endomembrane system	6.52
GO:0043233	C	organelle lumen	6.69
GO:0070013	C	intracellular organelle lumen	6.69
GO:0005618	C	cell wall	11.03
GO:0005829	C	cytosol	16.80
GO:0031981	C	nuclear lumen	8.43
GO:0005886	C	plasma membrane	51.98
GO:0005794	C	Golgi apparatus	6.87
GO:0044459	C	plasma membrane part	17.02
GO:0048046	C	apoplast	11.29
GO:0009579	C	thylakoid	11.66
GO:0015934	C	large ribosomal subunit	10.98
GO:0009536	C	plastid	54.98
GO:0031226	C	intrinsic to plasma membrane	13.95
GO:0005887	C	integral to plasma membrane	11.62
GO:0005773	C	vacuole	83.20
GO:0030054	C	cell junction	31.62
GO:0009507	C	chloroplast	122.38

GO:0005911	C	cell-cell junction	46.11
GO:0005730	C	nucleolus	79.04
GO:0044437	C	vacuolar part	158.08
GO:0005774	C	vacuolar membrane	158.08
GO:0009987	P	cellular process	1.43
GO:0006807	P	nitrogen compound metabolic process	1.69
GO:0050896	P	response to stimulus	3.88
GO:0006950	P	response to stress	3.10
GO:0044281	P	small molecule metabolic process	3.07
GO:0009628	P	response to abiotic stimulus	2.64
GO:0042180	P	cellular ketone metabolic process	3.30
GO:0006082	P	organic acid metabolic process	3.19
GO:0043436	P	oxoacid metabolic process	3.20
GO:0019752	P	carboxylic acid metabolic process	3.20
GO:0034641	P	cellular nitrogen compound metabolic process	3.85
GO:0009056	P	catabolic process	3.15
GO:0009308	P	amine metabolic process	4.13
GO:0006519	P	cellular amino acid and derivative metabolic process	4.33
GO:0044106	P	cellular amine metabolic process	4.22
GO:0006520	P	cellular amino acid metabolic process	3.17
GO:0044283	P	small molecule biosynthetic process	6.03
GO:0042221	P	response to chemical stimulus	10.61
GO:0055086	P	nucleobase, nucleoside and nucleotide metabolic process	3.32
GO:0006952	P	defense response	5.46
GO:0044271	P	cellular nitrogen compound biosynthetic process	5.18
GO:0009605	P	response to external stimulus	9.37
GO:0009611	P	response to wounding	4.53
GO:0009309	P	amine biosynthetic process	7.27
GO:0051716	P	cellular response to stimulus	9.76
GO:0033554	P	cellular response to stress	6.04
GO:0032787	P	monocarboxylic acid metabolic process	5.65
GO:0007154	P	cell communication	8.28
GO:0016265	P	death	6.29
GO:0008219	P	cell death	6.29
GO:0051704	P	multi-organism process	11.05
GO:0000003	P	reproduction	10.48
GO:0012501	P	programmed cell death	6.59
GO:0006820	P	anion transport	7.95
GO:0006575	P	cellular amino acid derivative metabolic process	16.47
GO:0006790	P	sulfur metabolic process	12.60
GO:0032502	P	developmental process	34.18
GO:0015698	P	inorganic anion transport	10.25
GO:0007275	P	multicellular organismal development	29.55
GO:0019438	P	aromatic compound biosynthetic process	12.16
GO:0044272	P	sulfur compound biosynthetic process	11.53
GO:0019748	P	secondary metabolic process	16.82
GO:0009066	P	aspartate family amino acid metabolic process	17.76

GO:0022414	P	reproductive process	20.46
GO:0006576	P	cellular biogenic amine metabolic process	13.17
GO:0010033	P	response to organic substance	48.30
GO:0009719	P	response to endogenous stimulus	35.13
GO:0009725	P	response to hormone stimulus	30.74
GO:0009067	P	aspartate family amino acid biosynthetic process	11.98
GO:0042398	P	cellular amino acid derivative biosynthetic process	36.28
GO:0009314	P	response to radiation	18.14
GO:0009416	P	response to light stimulus	18.14
GO:0000096	P	sulfur amino acid metabolic process	24.53
GO:0048878	P	chemical homeostasis	22.99
GO:0050801	P	ion homeostasis	14.37
GO:0000097	P	sulfur amino acid biosynthetic process	17.90
GO:0048519	P	negative regulation of biological process	19.36
GO:0048856	P	anatomical structure development	71.51
GO:0048731	P	system development	39.52
GO:0010035	P	response to inorganic substance	100.59
GO:0048518	P	positive regulation of biological process	33.53
GO:0048522	P	positive regulation of cellular process	33.53
GO:0006555	P	methionine metabolic process	44.46
GO:0042401	P	cellular biogenic amine biosynthetic process	34.58
GO:0009607	P	response to biotic stimulus	66.29
GO:0009086	P	methionine biosynthetic process	26.35
GO:0010038	P	response to metal ion	93.68
GO:0032535	P	regulation of cellular component size	29.27
GO:0090066	P	regulation of anatomical structure size	29.27
GO:0055082	P	cellular chemical homeostasis	45.16
GO:0009698	P	phenylpropanoid metabolic process	43.91
GO:0051707	P	response to other organism	118.56
GO:0009617	P	response to bacterium	49.40
GO:0070887	P	cellular response to chemical stimulus	158.08
GO:0048869	P	cellular developmental process	118.56
GO:0040007	P	growth	112.91
GO:0008361	P	regulation of cell size	158.08
GO:0048037	F	cofactor binding	2.90
GO:0050662	F	coenzyme binding	3.02
GO:0042578	F	phosphoric ester hydrolase activity	4.25
GO:0016616	F	oxidoreductase activity, acting on the CH-OH group of donors, NAD or NADP as acceptor	4.01
GO:0016791	F	phosphatase activity	5.01
GO:0016705	F	oxidoreductase activity, acting on paired donors, with incorporation or reduction of molecular oxygen	9.66
GO:0016765	F	transferase activity, transferring alkyl or aryl (other than methyl) groups	7.43
GO:0051287	F	NAD or NADH binding	7.96
GO:0016903	F	oxidoreductase activity, acting on the aldehyde or oxo group of donors	18.60
GO:0008483	F	transaminase activity	13.87
GO:0016620	F	oxidoreductase activity, acting on the aldehyde or oxo group of donors, NAD or NADP as acceptor	25.29

GO:0003993	F	acid phosphatase activity	20.80
GO:0015103	F	inorganic anion transmembrane transporter activity	27.90

[1] Ontolgy: C = cellular component; P = biological process; F = molecular function

[2] Fold enrichment of the GO-term as represented by the percentage of genes being differentially regulated in the RNA-Seq approach (FDR < 0.05) versus the percentage of genes with the corresponding GO-term in the reference gene set of maize

Supplemental Table 3 Genes differentially regulated at an experiment wide FDR < 0.05 across all two-way Comparisons

Gene	Position	Comp. 1 (p value)[1]	Comp. 2 (p value)	Comp. 3 (p value)	Comp. 4 (p value)	Comp. 1 (log2FC)[2]	Comp. 2 (log2FC)	Comp. 3 (log2FC)	Comp. 4 (log2FC)	Function / Accession[3]
GRMZM2G388684	1:2692900-2694270	1.36	n.s.	n.s.	n.s.	-3.18	-1.10	-0.20	-0.70	Uncharacterized protein [Source:Uniprot/SPTREMBL;Acc:B4FHS5]
GRMZM2G013634	1:4038301-4040423	2.00	n.s.	n.s.	n.s.	-2.71	-4.72	-1.18	-0.16	
GRMZM2G055575	1:5538007-5542785	5.21	5.20	n.s.	n.s.	-1.77	-2.89	-1.27	-1.40	Uncharacterized protein [Source:Uniprot/SPTREMBL;Acc:C0P3A2]
GRMZM2G090738	1:7164299-7168523	3.21	n.s.	n.s.	n.s.	2.16	1.38	-0.12	0.27	
GRMZM2G086179	1:10435984-10436526	11.21	n.s.	n.s.	n.s.	-3.27	-3.75	-0.89	-0.66	
GRMZM2G028521	1:10541840-10546402	n.s.	n.s.	n.s.	n.s.	-1.47	0	0	0	Uncharacterized protein [Source:Uniprot/SPTREMBL;Acc:B4FMP1]
GRMZM2G165998	1:12514730-12519380	3.19	n.s.	n.s.	n.s.	1.39	0.13	0.16	1.42	Uncharacterized protein [Source:Uniprot/SPTREMBL;Acc:B4FIQ9]
GRMZM2G556515	1:13996236-13997167	20	6.91	n.s.	n.s.	-7.10	-6.79	-0.12	-0.28	
GRMZM2G156486	1:15990465-15997559	10.35	n.s.	n.s.	n.s.	3.88	3.11	0.04	0.42	Uncharacterized protein [Source:Uniprot/SPTREMBL;Acc:B8A2V8]
GRMZM2G018984	1:17493459-17495464	12.88	n.s.	n.s.	n.s.	-2.85	-2.39	0.11	-0.13	Uncharacterized protein [Source:Uniprot/SPTREMBL;Acc:B4FBE6]
GRMZM2G410704	1:17721994-17725220	12.17	n.s.	n.s.	n.s.	-8.40	-9.30	-0.67	-0.21	
GRMZM2G061806	1:17782918-17784536	n.s.	1.93	n.s.	n.s.	0.56	3.30	0.20	-0.81	
GRMZM2G164539	1:19634360-19634656	20	20	n.s.	n.s.	-8.16	-8.28	0.07	0.12	
GRMZM2G055025	1:22627386-22633408	7.00	n.s.	n.s.	n.s.	2.11	1.26	-0.12	0.29	Outer mitochondrial membrane protein porin [Source:Uniprot/SPTREMBL;Acc:B6SS48]
GRMZM2G111324	1:27626301-27631083	2.39	3.59	n.s.	n.s.	2.26	2.67	0.12	0	Uncharacterized protein [Source:Uniprot/SPTREMBL;Acc:B4F8X5]
GRMZM2G152390	1:31997373-32001173	6.32	n.s.	n.s.	n.s.	-2.93	-0.85	0.20	-0.34	Uncharacterized protein [Source:Uniprot/SPTREMBL;Acc:B4FSX0]
GRMZM2G152447	1:32021587-32024355	12.88	n.s.	n.s.	n.s.	-3.99	-3.46	-0.33	-0.60	Uncharacterized protein [Source:Uniprot/SPTREMBL;Acc:B4FM74]
GRMZM2G360234	1:32756096-32757729	3.44	n.s.	n.s.	n.s.	-1.59	-2.01	-0.13	0.06	Uncharacterized protein [Source:Uniprot/SPTREMBL;Acc:B4FLV4]
GRMZM2G043878	1:41436542-41437697	n.s.	2.45	n.s.	n.s.	3.06	3.21	0.41	0.35	Uncharacterized protein [Source:Uniprot/SPTREMBL;Acc:B4FUR2]
GRMZM2G137839	1:43678295-43681497	2.90	n.s.	n.s.	n.s.	-1.29	-1.96	-0.78	-0.39	APx1-Cytosolic Ascorbate Peroxidase; Ascorbate peroxidase [Source:Uniprot/SPTREMBL;Acc:B6U9S6]
GRMZM2G319781	1:44027291-44029869	2.14	n.s.	n.s.	n.s.	-4.41	-1.30	-0.38	-2.52	
GRMZM2G124785	1:49320819-49323269	7.56	n.s.	n.s.	n.s.	1.91	0.34	0.28	1.75	Nicotianamine synthase 2 [Source:Uniprot/SPTREMBL;Acc:G3FCZ9]
GRMZM2G020940	1:53621182-53623074	5.25	n.s.	n.s.	n.s.	-1.81	-2.39	-0.13	0.15	Uncharacterized protein [Source:Uniprot/SPTREMBL;Acc:B4G0K5]

GRMZM2G378907	1:60092678-60101657	2.09	n.s.	n.s.	n.s.	-1.35	-0.69	0	-0.05	Tubby protein [Source:Uniprot/SPTREMBL;Acc:B6T7S6]
GRMZM2G038153	1:61012518-61018961	n.s.	6.19	n.s.	n.s.	-1.48	-2.54	0.37	1.78	Terpene synthase 8 [Source:Uniprot/SPTREMBL;Acc:Q29VN3]
GRMZM2G040515	1:63611561-63614648	3.23	n.s.	n.s.	n.s.	2.77	0.44	0	0.78	Uncharacterized protein [Source:Uniprot/SPTREMBL;Acc:C4J0E7]
GRMZM2G121990	1:65490013-65490687	20	n.s.	n.s.	n.s.	-2.65	-1.15	0.02	-0.17	
GRMZM2G350312	1:65657063-65660290	3.45	n.s.	n.s.	7.67	1.81	-0.27	0.37	3.07	BHLH transcription factor; Uncharacterized protein [Source:Uniprot/SPTREMBL;Acc:B8A2P8]
GRMZM2G412430	1:67848969-67851238	2.78	n.s.	n.s.	n.s.	-2.76	-1.09	-0.10	-0.38	Uncharacterized protein [Source:Uniprot/SPTREMBL;Acc:B4FTM7]
GRMZM2G048324	1:70424653-70429257	2.74	n.s.	n.s.	n.s.	1.11	0.10	-0.05	0.38	Uncharacterized protein [Source:Uniprot/SPTREMBL;Acc:B4FLB0]
GRMZM5G828488	1:70564124-70565403	2.99	n.s.	n.s.	n.s.	-4.39	-5	-5	-2.96	Ids4-like protein [Source:Uniprot/SPTREMBL;Acc:B6TC58]
GRMZM2G125241	1:73966090-73968225	2.05	n.s.	n.s.	n.s.	-1.72	-0.30	0.03	-0.54	Glucan endo-1,3-beta-glucosidase 4; Uncharacterized protein [Source:Uniprot/SPTREMBL;Acc:B6TIP4]
GRMZM2G074138	1:76609644-76615925	2.00	n.s.	n.s.	n.s.	-1.52	-0.54	0.21	-0.02	Sigma factor sigB regulation protein rsbQ; Uncharacterized protein [Source:Uniprot/SPTREMBL;Acc:B4FHV3]
GRMZM2G084063	1:76839834-76842563	3.36	n.s.	n.s.	n.s.	-1.47	-0.49	-0.05	-0.19	Uncharacterized protein [Source:Uniprot/SPTREMBL;Acc:B6SIA6]
GRMZM2G066369	1:81639292-81640765	1.40	n.s.	n.s.	n.s.	3.42	5	-0.61	-5	Glutathione S-transferase GST 38 [Source:Uniprot/SPTREMBL;Acc:Q9FQA1]
GRMZM2G125304	1:82488504-82496043	1.46	n.s.	n.s.	n.s.	-3.66	-3.65	0	0	Uncharacterized protein [Source:Uniprot/SPTREMBL;Acc:C4IY64]
GRMZM2G047187	1:85829899-85834581	1.51	n.s.	n.s.	n.s.	-10	-5	0	-0.41	Uncharacterized protein [Source:Uniprot/SPTREMBL;Acc:B4F9E5]
GRMZM5G887922	1:95485355-95487308	1.76	n.s.	n.s.	n.s.	1.83	0.76	-0.35	-0.25	Retrotransposon protein [Source:Uniprot/SPTREMBL;Acc:B6SJU9]
GRMZM2G179325	1:155675314-155678789	4.19	n.s.	n.s.	n.s.	-2.26	-2.33	-0.37	-0.35	
GRMZM2G131907	1:164639294-164642137	20	n.s.	n.s.	n.s.	2.17	0.39	0.32	2.01	Uncharacterized protein [Source:Uniprot/SPTREMBL;Acc:B4FS03]
GRMZM2G047875	1:174224914-174229713	n.s.	n.s.	n.s.	n.s.	-0.64	0	0	0	
GRMZM2G037265	1:183557958-183561991	n.s.	n.s.	n.s.	n.s.	-1.30	-2.81	0	0	Uncharacterized protein [Source:Uniprot/SPTREMBL;Acc:B8A2Z1]
GRMZM2G088627	1:189145768-189150346	4.21	n.s.	n.s.	n.s.	2.04	0.57	-0.18	0.26	Aminoacylase-1 [Source:Uniprot/SPTREMBL;Acc:B6T4W6]
GRMZM2G171111	1:194210576-194219751	5.53	n.s.	n.s.	n.s.	1.76	0.29	0.22	1.57	Uncharacterized protein [Source:Uniprot/SPTREMBL;Acc:B4FDZ6]
GRMZM2G136453	1:195331029-195338316	5.14	n.s.	n.s.	n.s.	1.68	2.52	0.09	-0.34	Uncharacterized protein [Source:Uniprot/SPTREMBL;Acc:B4FA21]
GRMZM2G106980	1:203647045-203653762	20	20	n.s.	n.s.	-3.80	-4.25	-0.75	-0.50	Uncharacterized protein [Source:Uniprot/SPTREMBL;Acc:B8A121]

GRMZM2G089528	1:207274163-207276641	3.57	n.s.	n.s.	n.s.	-2.18	-3.17	-0.86	-0.38	Flavonoid 3-monooxygenase; Uncharacterized protein [Source:Uniprot/SPTREMBL;Acc:B4FQ24]
GRMZM2G059465	1:209119390-209120903	n.s.	n.s.	n.s.	6.60	-0.04	0.42	-1.54	-2.49	Uncharacterized protein [Source:Uniprot/SPTREMBL;Acc:B8A095]
GRMZM2G161245	1:213097240-213099953	6.39	n.s.	n.s.	n.s.	2.59	1.51	-0.36	0.15	Malate dehydrogenase [Source:Uniprot/SPTREMBL;Acc:B4FG53]
GRMZM2G160560	1:214111609-214117150	2.93	n.s.	n.s.	n.s.	-4.10	-2.76	0.18	-0.43	Transmembrane protein PFT27; Uncharacterized protein [Source:Uniprot/SPTREMBL;Acc:B6TV17]
GRMZM2G003304	1:218716368-218721179	5.25	n.s.	n.s.	n.s.	-1.78	-0.36	-0.16	-0.69	Uncharacterized protein [Source:Uniprot/SPTREMBL;Acc:B7ZYZ9]
GRMZM2G024145	1:222719188-222723682	n.s.	2.16	n.s.	n.s.	-6.44	-4.27	0.86	-0.23	Serine incorporator 3; Uncharacterized protein [Source:Uniprot/SPTREMBL;Acc:B4FAN1]
GRMZM2G176209	1:223842176-223846120	1.46	n.s.	n.s.	n.s.	-2.33	-3.06	-0.70	-0.34	Aquaporin NIP3-1 [Source:Uniprot/SWISSPROT;Acc:Q9ATN1]
GRMZM2G093951	1:228664949-228666474	1.63	n.s.	n.s.	n.s.	-4.68	0.23	1.31	-2.53	Uncharacterized protein [Source:Uniprot/SPTREMBL;Acc:C0PDX3]
GRMZM2G085974	1:231155368-231157244	20	8.88	n.s.	n.s.	-4.44	-3.48	0.30	-0.18	Uncharacterized protein [Source:Uniprot/SPTREMBL;Acc:C0PB98]
GRMZM2G013131	1:234696672-234697372	2.42	n.s.	n.s.	n.s.	1.01	0.35	-0.08	0.06	
GRMZM2G490997	1:234697760-234698386	3.11	n.s.	n.s.	n.s.	1.21	0.46	0.00	0.12	
GRMZM2G038898	1:238463703-238466430	6.12	4.95	n.s.	n.s.	-3.15	-3.19	0.11	0.14	Uncharacterized protein [Source:Uniprot/SPTREMBL;Acc:C0PC24]
GRMZM2G083156	1:244976753-244978209	2.36	n.s.	n.s.	n.s.	2.33	3.07	-0.20	-0.58	
GRMZM2G092825	1:246993896-246996587	1.34	n.s.	n.s.	n.s.	1.16	1.58	-0.14	-0.38	
GRMZM2G120621	1:247941255-247945054	1.70	n.s.	n.s.	n.s.	-3.47	-0.93	-0.20	-1.01	Uncharacterized protein [Source:Uniprot/SPTREMBL;Acc:C0P556]
GRMZM2G043127	1:253796236-253799897	3.08	n.s.	n.s.	n.s.	-2.57	-1.96	0.22	-0.09	Uncharacterized protein [Source:Uniprot/SPTREMBL;Acc:B4FCR0]
GRMZM2G027663	1:256566871-256573278	1.32	1.41	n.s.	n.s.	-3.31	-3.24	-0.03	-0.07	Uncharacterized protein [Source:Uniprot/SPTREMBL;Acc:C0PEH3]
GRMZM2G118821	1:258353072-258355277	20	4.99	n.s.	n.s.	-2.98	-2.19	0.42	0.02	Uncharacterized protein; Zinc transporter 10 [Source:Uniprot/SPTREMBL;Acc:B6U8Z3]
GRMZM2G423972	1:264155924-264160052	3.92	n.s.	n.s.	2.43	2.11	0.18	0.18	2.06	
GRMZM2G042133	1:265023533-265154204	20	20	n.s.	n.s.	3.50	3.41	0.11	0.15	
GRMZM2G127521	1:268343581-268346959	4.48	4.41	n.s.	n.s.	4.10	7.10	-0.22	-0.25	
GRMZM2G583462	1:269468231-269469313	n.s.	1.44	n.s.	n.s.	9.49	8.67	-0.06	0.37	Uncharacterized protein [Source:Uniprot/SPTREMBL;Acc:K7UIK7]
GRMZM2G149952	1:275090702-275094517	20	7.35	n.s.	n.s.	3.08	2.49	-0.28	0.05	Uncharacterized protein [Source:Uniprot/SPTREMBL;Acc:C0P6J3]
GRMZM2G483308	1:282125428-282125646	1.74	n.s.	n.s.	n.s.	5.87	5.93	0.37	-0.04	
GRMZM2G035890	1:286240395-286242460	n.s.	4.39	n.s.	n.s.	1.33	2.13	1.61	0.43	

GRMZM2G016150	1:288207520-288212296	1.44	n.s.	n.s.	n.s.	-2.80	-2.84	0.20	0.18	Opaque2 heterodimerizing protein 1 [Source:Uniprot/SPTREMBL;Acc:Q03462]
GRMZM2G049790	1:289274299-289275170	1.58	n.s.	n.s.	n.s.	-3.23	-2.88	0.12	-0.07	Putative uncharacterized protein [Source:Uniprot/SPTREMBL;Acc:B6TNU7]
GRMZM5G853702	1:293997055-293998873	n.s.	n.s.	1.79	n.s.	-1.18	-0.29	2.53	0.96	Uncharacterized protein [Source:Uniprot/SPTREMBL;Acc:C4J6Z2]
GRMZM2G177438	1:294465873-294471943	1.68	n.s.	n.s.	n.s.	3.23	2.87	0	0.15	
GRMZM2G119755	1:295630216-295631181	4.71	n.s.	n.s.	n.s.	-2.44	-0.41	0.37	-0.45	Cell number regulator 7 [Source:Uniprot/SWISSPROT;Acc:D9HP23]
GRMZM2G371857	2:3074888-3077194	3.03	n.s.	n.s.	n.s.	-3.57	-3.78	-0.25	-0.16	
GRMZM2G042055	2:7290713-7293868	5.63	n.s.	n.s.	n.s.	-2.32	-0.58	0.20	-0.38	
GRMZM2G584110	2:7848275-7848502	3.59	2.79	n.s.	n.s.	-6.17	-4.22	0.83	-0.15	
GRMZM2G575328	2:7945776-7946002	20	20	n.s.	n.s.	5.73	5.82	-0.01	-0.04	
GRMZM5G876898	2:8906079-8909067	5.15	n.s.	n.s.	n.s.	-1.73	-2.02	-0.24	-0.10	Aminomethyltransferase [Source:Uniprot/SPTREMBL;Acc:B6TQ06]
GRMZM2G040397	2:9502966-9507292	1.85	n.s.	n.s.	n.s.	-1.68	-0.77	0.06	-0.02	Uncharacterized protein [Source:Uniprot/SPTREMBL;Acc:C0P4R8]
GRMZM2G094328	2:9547649-9549271	2.81	n.s.	n.s.	n.s.	1.52	2.69	0.18	-0.43	Germin-like protein subfamily 1 member 11 [Source:Uniprot/SPTREMBL;Acc:B6TTY1]
GRMZM2G007157	2:9998299-10004356	2.56	n.s.	n.s.	n.s.	2.08	1.73	0.07	0	Uncharacterized protein [Source:Uniprot/SPTREMBL;Acc:C4IZK0]
GRMZM2G071907	2:11753530-11755126	1.55	n.s.	n.s.	n.s.	-2.05	-0.60	0.20	-0.23	Uncharacterized protein [Source:Uniprot/SPTREMBL;Acc:B4FEI8]
GRMZM2G176225	2:12600465-12603346	3.05	n.s.	n.s.	n.s.	-3.73	-3.46	-0.48	-0.62	WAK53a-OsWAK receptor-like protein kinase [Source:Uniprot/SPTREMBL;Acc:B6SYA6]
GRMZM2G149649	2:12677823-12681229	3.70	2.15	n.s.	n.s.	-3.09	-3.40	-0.05	0.11	60S ribosomal protein L12; Uncharacterized protein [Source:Uniprot/SPTREMBL;Acc:B4FRM7]
GRMZM2G032218	2:13856244-13864605	2.39	n.s.	n.s.	n.s.	1.29	0.47	-0.32	-0.20	
GRMZM2G322819	2:17861165-17865525	6.28	n.s.	n.s.	n.s.	-3.58	-5.05	-1.01	-0.03	
GRMZM2G145045	2:28195145-28198901	2.25	n.s.	n.s.	n.s.	-3.15	-1.18	-0.13	-0.53	
GRMZM2G145075	2:28233887-28237813	3.81	n.s.	n.s.	n.s.	-1.93	-0.49	0.10	-0.38	
GRMZM2G145109	2:28274091-28278099	4.14	n.s.	n.s.	n.s.	-2.43	-0.74	0.17	-0.31	Uncharacterized protein [Source:Uniprot/SPTREMBL;Acc:B4F8T7]
GRMZM2G051943	2:33534100-33535449	2.10	n.s.	n.s.	n.s.	-3.77	-2.29	0.83	0.08	Endochitinase A [Source:Uniprot/SWISSPROT;Acc:P29022]
GRMZM2G009892	2:41885687-41887591	1.50	n.s.	n.s.	n.s.	-3.15	-2.13	-0.27	-0.71	Uncharacterized protein [Source:Uniprot/SPTREMBL;Acc:C0P5H4]
GRMZM2G056507	2:45048585-45049754	3.01	n.s.	n.s.	n.s.	2.44	-0.13	-0.14	1.98	
GRMZM2G044132	2:53844492-53846005	20	2.93	n.s.	n.s.	-4.02	-3.11	-0.34	-0.80	Putative uncharacterized protein [Source:Uniprot/SPTREMBL;Acc:B6UCP6]

GRMZM5G800014	2:55482906-55484252	5.26	n.s.	n.s.	n.s.	-2.62	-3.15	-0.53	-0.27	Uncharacterized protein [Source:Uniprot/SPTREMBL;Acc:C0P9B2]
GRMZM2G074238	2:57643339-57646559	1.32	n.s.	n.s.	n.s.	-4.18	-0.60	1.22	-0.28	Uncharacterized protein [Source:Uniprot/SPTREMBL;Acc:B4FYI3]
GRMZM2G157027	2:78496008-78501564	3.49	n.s.	n.s.	n.s.	3.20	0.74	-0.36	0.49	Uncharacterized protein [Source:Uniprot/SPTREMBL;Acc:B4FFA5]
GRMZM2G171552	2:100217866-100219316	n.s.	n.s.	n.s.	2.20	1.79	0.36	0.46	2.03	
GRMZM2G045809	2:106286426-106287698	4.66	n.s.	n.s.	n.s.	2.11	0.00	-0.37	0.68	Germin-like protein subfamily 1 member 17; Uncharacterized protein [Source:Uniprot/SPTREMBL;Acc:B4FUT3]
GRMZM2G118759	2:130277984-130278842	4.24	n.s.	n.s.	n.s.	1.66	1.67	0.30	0.26	Glycine-rich cell wall structural protein 2 [Source:Uniprot/SPTREMBL;Acc:B6TEE0]
GRMZM2G018649	2:168292353-168293777	n.s.	2.84	n.s.	n.s.	4.10	4.46	0.12	0.17	
GRMZM2G104563	2:172843793-172863857	3.65	n.s.	n.s.	n.s.	1.69	-0.14	0.09	2.14	
GRMZM2G080466	2:189499627-189501644	n.s.	2.73	n.s.	n.s.	-1.33	-2.77	-0.57	0.14	Uncharacterized protein [Source:Uniprot/SPTREMBL;Acc:C4J9K1]
GRMZM2G134334	2:189571461-189572675	2.86	n.s.	n.s.	n.s.	-1.58	-2.94	0.01	0.68	Multiple stress-responsive zinc-finger protein ISAP1 [Source:Uniprot/SPTREMBL;Acc:B6UIJ0]
GRMZM2G022032	2:193829791-193833616	4.80	n.s.	n.s.	n.s.	-1.77	-0.68	0.19	-0.01	
GRMZM2G448247	2:199209920-199220265	2.59	n.s.	n.s.	n.s.	-3.99	-1.14	0.31	-0.56	
GRMZM2G026532	2:203521403-203523650	2.39	n.s.	n.s.	n.s.	-2.75	-5	-5	-1.87	Uncharacterized protein [Source:Uniprot/SPTREMBL;Acc:B4FRN1]
GRMZM2G101769	2:211210657-211213272	4.23	n.s.	n.s.	n.s.	-1.88	-2.13	-0.31	-0.20	TIFY transcription factor; Uncharacterized protein [Source:Uniprot/SPTREMBL;Acc:B4G1J1]
GRMZM2G124919	2:211533716-211538264	6.99	n.s.	n.s.	n.s.	2.70	0.81	0.16	1.97	
GRMZM2G056612	2:211733206-211739189	3.14	n.s.	n.s.	n.s.	-2.55	-2.69	-0.07	0.00	Uncharacterized protein [Source:Uniprot/SPTREMBL;Acc:C0PIB1]
GRMZM2G092877	2:213341767-213343903	1.83	n.s.	n.s.	n.s.	-4.43	-5.78	0.00	0.67	Uncharacterized protein [Source:Uniprot/SPTREMBL;Acc:B7ZYD5]
GRMZM2G172330	2:219804156-219808169	n.s.	2.38	n.s.	n.s.	-10	-6.71	5	0.18	Brassinosteroid LRR receptor kinase [Source:Uniprot/SPTREMBL;Acc:B6U7C7]
GRMZM2G171818	2:220669657-220672227	1.87	n.s.	n.s.	n.s.	-2.03	-0.61	0.26	-0.14	BHLH transcription factor [Source:Uniprot/SPTREMBL;Acc:B6UBF8]
GRMZM2G349839	2:220725728-220729080	4.24	n.s.	n.s.	n.s.	3.60	4.23	-0.11	-0.44	
GRMZM2G074496	2:225227431-225230275	2.53	n.s.	n.s.	n.s.	-4.63	-2.12	0.59	0.28	
GRMZM2G158175	2:227594473-227598371	2.02	n.s.	n.s.	n.s.	-4.08	-1.67	-0.74	-2.23	
GRMZM2G100700	2:229109985-229111240	3.45	n.s.	n.s.	n.s.	-1.29	-0.73	-0.08	0.00	Cytochrome c biogenesis C [Source:Uniprot/SPTREMBL;Acc:Q0GR56]
GRMZM2G178890	2:233961265-233962444	1.31	n.s.	n.s.	n.s.	2.52	2.34	0.06	0.12	
GRMZM2G399844	2:234223534-234227624	2.31	n.s.	n.s.	n.s.	-3.83	-4.98	0	0	Uncharacterized protein [Source:Uniprot/SPTREMBL;Acc:C0PFB3]

GRMZM2G520154	2:234409396-234409729	20	n.s.	n.s.	n.s.	-3.95	-3.24	0.24	-0.12	
GRMZM2G099678	3:2001769-2004402	1.31	n.s.	n.s.	n.s.	3.58	5.12	0.12	-0.65	Vegetative storage protein PNI288 [Source:Uniprot/SPTREMBL;Acc:B6T4U2]
GRMZM2G178738	3:6583937-6584465	11.21	n.s.	n.s.	n.s.	1.99	0.49	0.18	1.32	
GRMZM2G023952	3:13068004-13070604	4.24	n.s.	n.s.	n.s.	-2.44	-0.46	-0.08	-1.67	Cytochrome P450 monooxygenase [Source:Uniprot/SPTREMBL;Acc:C4PJN6]
GRMZM2G158342	3:13101530-13103292	4.32	n.s.	n.s.	n.s.	-1.64	0.15	-0.15	-2.26	
GRMZM2G102047	3:15472504-15473455	20	n.s.	n.s.	n.s.	-4.48	-3.17	-0.53	-2.39	
GRMZM2G132678	3:20795800-20806607	2.63	4.50	n.s.	n.s.	5.35	4.50	0.02	0.44	
GRMZM2G032107	3:22570387-22571485	2.51	2.22	n.s.	n.s.	-2.80	-3.38	0.03	0.31	Putative uncharacterized protein [Source:Uniprot/SPTREMBL;Acc:B6U3Q6]
GRMZM2G491268	3:27434967-27435593	3.11	n.s.	n.s.	n.s.	1.21	0.43	0	0.09	
GRMZM2G049895	3:32033592-32037043	3.45	n.s.	n.s.	n.s.	-1.70	-0.71	0.03	-0.12	Uncharacterized protein [Source:Uniprot/SPTREMBL;Acc:C4IYD7]
GRMZM2G701449	3:41135860-41136173	20	20	n.s.	n.s.	-3.71	-4.29	-0.22	0.07	
GRMZM5G819302	3:49863490-49867479	2.55	n.s.	n.s.	n.s.	-1.77	-0.40	0.33	-0.11	
GRMZM2G169562	3:129296727-129301658	3.99	n.s.	n.s.	n.s.	-3.98	-3.81	-0.50	-0.59	
GRMZM2G032198	3:130520349-130521198	3.39	n.s.	n.s.	n.s.	-1.25	-0.63	0.00	0.00	
GRMZM2G374405	3:130829487-130830015	6.19	5.00	n.s.	n.s.	2.86	4.69	-0.08	-1.02	
GRMZM2G030790	3:131609759-131610735	8.49	n.s.	n.s.	n.s.	-7.13	-5	-5	-3.04	Jasmonate-induced protein [Source:Uniprot/SPTREMBL;Acc:B6TLX4]
GRMZM2G140101	3:131702643-131704144	5.39	6.25	n.s.	n.s.	2.11	3.34	-0.07	-0.72	Phytase [Source:Uniprot/SPTREMBL;Acc:Q9T0N7]
GRMZM2G175761	3:134613811-134615464	3.36	n.s.	n.s.	n.s.	-2.07	-0.61	-0.10	-0.53	Pollen-specific protein SF3 [Source:Uniprot/SPTREMBL;Acc:B6SXB1]
GRMZM2G179465	3:146643021-146645957	1.49	n.s.	n.s.	n.s.	3.83	3.04	-0.18	0.20	Putative uncharacterized protein [Source:Uniprot/SPTREMBL;Acc:B6SHV5]
GRMZM2G057413	3:148031451-148062994	n.s.	n.s.	n.s.	2.78	3.40	0	0.48	3.77	
GRMZM2G400602	3:174361241-174363882	n.s.	n.s.	n.s.	8.89	2.20	-0.09	0.46	3.29	Uncharacterized protein [Source:Uniprot/SPTREMBL;Acc:C0HEP6]
GRMZM2G078314	3:175868331-175869457	n.s.	2.75	n.s.	n.s.	-0.52	-2.73	-0.44	0.40	Histone H3 [Source:Uniprot/SPTREMBL;Acc:B6TTP0]
GRMZM2G053284	3:175897256-175900286	2.52	n.s.	n.s.	n.s.	-1.69	-0.79	-0.22	-0.28	Uncharacterized protein [Source:Uniprot/SPTREMBL;Acc:B4FV86]
GRMZM2G085577	3:178982015-178990918	3.91	n.s.	n.s.	n.s.	1.72	0.20	-0.31	0.33	Phosphorylase [Source:Uniprot/SPTREMBL;Acc:B5AMJ8]
GRMZM5G866320	3:185576301-185576927	3.11	n.s.	n.s.	n.s.	1.21	0.43	0	0.09	
GRMZM2G344630	3:185932046-185933025	1.76	n.s.	n.s.	n.s.	-2.95	-1.30	0.22	0.04	GTP binding protein [Source:Uniprot/SPTREMBL;Acc:B6SS88]
GRMZM2G306143	3:188925684-188929697	2.27	n.s.	n.s.	n.s.	1.72	1.95	-0.26	-0.39	

GRMZM2G054905	3:191124530-191185971	10.82	4.19	n.s.	n.s.	2.33	2.11	0.03	0.14	
GRMZM2G057140	3:191413501-191495940	3.61	1.40	n.s.	n.s.	-5.25	-3.98	-0.01	-0.25	
GRMZM2G058931	3:194920162-194925079	5.46	3.62	n.s.	n.s.	3.32	4.11	-0.01	-0.40	
GRMZM2G127665	3:196137227-196147905	4.48	n.s.	n.s.	n.s.	-2.11	-2.52	-0.09	0.06	
GRMZM2G015908	3:202182373-202183608	2.88	n.s.	n.s.	n.s.	-4.88	-1.99	-0.52	-1.94	Phosphatase phospho1 [Source:Uniprot/SPTREMBL;Acc:B6TXH8]
GRMZM2G312712	3:207063509-207079840	3.24	n.s.	n.s.	n.s.	1.75	0.08	-0.01	0.73	
GRMZM5G896496	3:211872426-211879044	3.81	1.65	n.s.	n.s.	-5.10	-5.00	0.09	0.03	Uncharacterized protein [Source:Uniprot/SPTREMBL;Acc:C0HH28]
GRMZM2G125193	3:215654138-215663757	1.75	n.s.	n.s.	n.s.	2.16	0.54	-0.14	0.39	Uncharacterized protein [Source:Uniprot/SPTREMBL;Acc:C4J093]
GRMZM2G180699	3:217630821-217633671	2.47	n.s.	n.s.	n.s.	-2.94	-1.11	0.09	-0.19	GTPase activating protein; Uncharacterized protein [Source:Uniprot/SPTREMBL;Acc:B4FFK7]
GRMZM2G071154	3:226763646-226771691	2.10	n.s.	n.s.	n.s.	-1.24	-0.50	-0.33	-0.46	Uncharacterized protein [Source:Uniprot/SPTREMBL;Acc:B4F858]
GRMZM2G065989	3:227425324-227426759	8.08	n.s.	n.s.	n.s.	-3.15	-0.89	0.02	-0.68	
GRMZM2G159908	3:229308914-229310895	4.08	n.s.	n.s.	n.s.	-1.47	-0.68	0.06	0.00	Uncharacterized protein [Source:Uniprot/SPTREMBL;Acc:B4FE65]
GRMZM2G181081	3:229904997-229916936	n.s.	2.77	n.s.	n.s.	-3.34	-3.77	0.39	0.48	CIPK-like protein 1 [Source:Uniprot/SPTREMBL;Acc:B6SY58]
GRMZM2G457287	4:1992636-1995216	3.42	n.s.	n.s.	n.s.	-2.90	-1.36	-0.30	-0.40	Uncharacterized protein [Source:Uniprot/SPTREMBL;Acc:K7U8I5]
GRMZM2G020508	4:2621434-2625622	5.12	1.66	n.s.	n.s.	-4.36	-3.51	-0.28	-0.58	
GRMZM2G039630	4:4477135-4477981	2.25	n.s.	n.s.	n.s.	-3.34	-0.59	0.56	-0.53	Uncharacterized protein [Source:Uniprot/SPTREMBL;Acc:K7TQY9]
GRMZM2G145213	4:5962320-5967839	3.57	n.s.	n.s.	n.s.	2.23	1.34	0.13	0.57	14-3-3-like protein; Uncharacterized protein [Source:Uniprot/SPTREMBL;Acc:B4FEW4]
GRMZM2G117971	4:9680498-9681375	n.s.	2.19	n.s.	n.s.	0.42	3.41	1.86	-0.33	Uncharacterized protein [Source:Uniprot/SPTREMBL;Acc:B4F9D6]
GRMZM5G870077	4:11705447-11707400	4.67	n.s.	n.s.	n.s.	-1.67	-0.70	1.52	0.62	Uncharacterized protein [Source:Uniprot/SPTREMBL;Acc:C0PDT7]
GRMZM2G017557	4:11916302-11917798	4.28	n.s.	n.s.	n.s.	-1.71	-0.55	-0.61	-1.86	O-methyltransferase ZRP4 [Source:Uniprot/SWISSPROT;Acc:P47917]
GRMZM2G139533	4:12777381-12783007	20	n.s.	n.s.	n.s.	3.23	1.72	0.10	1.70	Methylthioribose-1-phosphate isomerase [Source:Uniprot/SWISSPROT;Acc:B6TZD1]
GRMZM5G866334	4:14632235-14634105	3.25	n.s.	n.s.	n.s.	-1.69	-0.78	-0.23	-0.30	Uncharacterized protein [Source:Uniprot/SPTREMBL;Acc:B4F878]
GRMZM2G070011	4:15297908-15299396	2.44	n.s.	n.s.	n.s.	-2.93	-1.29	-1.22	-2.80	Uncharacterized protein; Vignain [Source:Uniprot/SPTREMBL;Acc:B4FZ79]
GRMZM2G107591	4:17141693-17143298	4.30	n.s.	n.s.	n.s.	-3.74	-4.24	-0.61	-0.41	Uncharacterized protein [Source:Uniprot/SPTREMBL;Acc:K7TZV6]
GRMZM2G441753	4:18221971-18222862	2.11	n.s.	n.s.	n.s.	-1.91	-0.70	0.00	-0.22	

GRMZM2G574782	4:18676198-18682894	8.91	n.s.	n.s.	4.26	1.74	0.11	0.21	1.92	Probable bifunctional methylthioribulose-1-phosphate dehydratase/enolase-phosphatase E1 Methylthioribulose-1-phosphate dehydratase Enolase-phosphatase E1 [Source:Uniprot/SWISSPROT;Acc:B4G0F3]
GRMZM2G014902	4:32498151-32531144	9.96	n.s.	n.s.	n.s.	2.13	0.42	0.07	0.69	Putative MYB DNA-binding domain superfamily protein [Source:Uniprot/SPTREMBL;Acc:K7U156]
GRMZM2G157443	4:37937136-37938763	3.49	n.s.	n.s.	n.s.	-2.24	-1.71	0.40	0.13	60S acidic ribosomal protein P1; 60S acidic ribosomal protein P1 isoform 1; 60S acidic ribosomal protein P1 isoform 2; 60S acidic ribosomal protein P1 isoform 3; Uncharacterized protein [Source:Uniprot/SPTREMBL;Acc:B4FUB4]
GRMZM2G112792	4:38235714-38238075	4.91	n.s.	n.s.	n.s.	2.11	2.55	-0.02	-0.29	Uncharacterized protein [Source:Uniprot/SPTREMBL;Acc:K7U1M0]
GRMZM2G088317	4:43167408-43168598	3.31	n.s.	n.s.	n.s.	-1.18	-0.55	0.15	0.11	
GRMZM2G097395	4:44953328-44958944	4.11	n.s.	n.s.	n.s.	1.75	0.24	-0.08	0.54	Nodulin-like protein [Source:Uniprot/SPTREMBL;Acc:K7USY0]
GRMZM2G425999	4:55074185-55085664	7.26	n.s.	n.s.	n.s.	2.14	1.32	-0.15	0.24	Uncharacterized protein [Source:Uniprot/SPTREMBL;Acc:K7UTN9]
GRMZM2G164146	4:80651593-80727070	2.12	n.s.	n.s.	n.s.	2.32	-0.06	-0.07	2.36	
GRMZM2G458845	4:89097335-89099983	n.s.	n.s.	n.s.	n.s.	1.71	0	0	0	
GRMZM2G370915	4:89101671-89102955	n.s.	n.s.	n.s.	n.s.	1.62	1.56	0	0	Uncharacterized protein [Source:Uniprot/SPTREMBL;Acc:K7UV98]
GRMZM2G492492	4:111745452-111745892	1.53	n.s.	n.s.	n.s.	1.14	0.38	0.01	0.18	
GRMZM2G000380	4:123666104-123668701	2.78	n.s.	n.s.	n.s.	-1.52	-0.20	0.10	-0.44	Uncharacterized protein [Source:Uniprot/SPTREMBL;Acc:K7TXF9]
GRMZM2G138640	4:133929995-133930505	5.37	n.s.	n.s.	n.s.	1.57	0.25	0.09	0.60	
GRMZM2G310410	4:153531421-153537080	2.43	n.s.	n.s.	n.s.	-2.95	-2.68	-0.03	-0.17	Uncharacterized protein [Source:Uniprot/SPTREMBL;Acc:K7UXZ3]
GRMZM2G046348	4:153749854-153753146	2.96	n.s.	n.s.	n.s.	-1.88	-2.11	-0.14	-0.03	Uncharacterized protein [Source:Uniprot/SPTREMBL;Acc:B4FCZ8]
GRMZM2G549241	4:156784269-156784709	2.22	n.s.	n.s.	n.s.	1.16	0.43	0.01	0.13	
GRMZM2G342515	4:160954462-160955623	3.05	n.s.	n.s.	n.s.	-4.43	-6.75	-1.20	0.22	Histone H2B.5 [Source:Uniprot/SWISSPROT;Acc:P54348]
GRMZM2G065030	4:162271172-162273744	7.89	n.s.	n.s.	n.s.	2.34	0.36	0.26	2.05	Uncharacterized protein [Source:Uniprot/SPTREMBL;Acc:K7UYT7]
GRMZM2G161746	4:166243311-166245777	4.13	n.s.	n.s.	n.s.	1.97	-0.06	-0.08	0.94	Uncharacterized protein [Source:Uniprot/SPTREMBL;Acc:C0HF51]
GRMZM2G142777	4:186835238-186837134	2.44	n.s.	n.s.	n.s.	-2.31	-2.34	-0.21	-0.17	Uncharacterized protein [Source:Uniprot/SPTREMBL;Acc:C0P3B4]
GRMZM5G891282	4:187449296-187450591	4.58	n.s.	n.s.	n.s.	2.97	0.48	0.18	2.31	Ribose-5-phosphate isomerase; Uncharacterized protein [Source:Uniprot/SPTREMBL;Acc:C0PBH7]

GRMZM2G428035	4:189366896-189408688	9.33	5.68	n.s.	n.s.	-4.02	-4.03	-0.01	-0.01	Uncharacterized protein [Source:Uniprot/SPTREMBL;Acc:K7U3J4]
GRMZM2G067865	4:191294315-191295835	2.47	n.s.	n.s.	n.s.	-2.59	-1.17	-0.60	-0.74	Putative RING zinc finger domain superfamily protein [Source:Uniprot/SPTREMBL;Acc:K7U3P8]
GRMZM2G446426	4:191542396-191544646	4.08	n.s.	n.s.	n.s.	3.58	3.07	-0.11	0.13	Putative MADS-box transcription factor family protein [Source:Uniprot/SPTREMBL;Acc:K7U795]
GRMZM2G332660	4:194112156-194114320	1.38	n.s.	n.s.	n.s.	4.66	1.68	0.31	1.79	Putative calcium-dependent protein kinase family protein [Source:Uniprot/SPTREMBL;Acc:K7U3W7]
GRMZM2G096958	4:219581607-219585123	8.27	n.s.	n.s.	5.03	1.71	-0.08	0.07	1.98	Nicotianamine aminotransferase1; Uncharacterized protein [Source:Uniprot/SPTREMBL;Acc:B4FEZ7]
GRMZM2G110843	4:231145931-231149829	1.37	n.s.	n.s.	n.s.	-3.66	-2.37	0.47	-0.18	Uncharacterized protein [Source:Uniprot/SPTREMBL;Acc:B6SQ76]
GRMZM2G506447	4:235464791-235465351	4.51	n.s.	n.s.	n.s.	1.31	1.44	-0.10	-0.20	
GRMZM2G548321	4:236950366-236950576	20	n.s.	n.s.	n.s.	-3.73	-3.58	0.04	-0.04	
GRMZM2G362021	4:238337298-238350692	7.46	n.s.	n.s.	3.81	2.35	0.28	0.19	2.11	Uncharacterized protein [Source:Uniprot/SPTREMBL;Acc:K7UBY2]
GRMZM2G366973	4:239081136-239095955	3.08	n.s.	n.s.	n.s.	-3.40	-0.88	0.61	-0.22	
GRMZM2G010280	4:240232549-240234575	n.s.	n.s.	n.s.	20	-0.60	0.79	-0.16	-3.12	High affinity nitrate transporter; Putative high affinity nitrate transporter [Source:Uniprot/SPTREMBL;Acc:Q53CL7]
GRMZM2G010251	4:240243499-240245403	n.s.	n.s.	n.s.	7.82	-0.11	2.16	-0.09	-2.58	High affinity nitrate transporter; Uncharacterized protein [Source:Uniprot/SPTREMBL;Acc:B4FSV9]
GRMZM2G136854	5:1235620-1243504	1.80	n.s.	n.s.	n.s.	2.10	0	0	0	
GRMZM2G072071	5:6877418-6883253	3.38	n.s.	n.s.	n.s.	1.30	0.41	0.00	0.22	Uncharacterized protein [Source:Uniprot/SPTREMBL;Acc:B8A1R5]
GRMZM2G104313	5:9798625-9798781	10.22	n.s.	n.s.	n.s.	-7.11	-8.80	-0.80	0.06	
GRMZM2G486936	5:23161903-23162462	n.s.	1.95	n.s.	n.s.	6.13	5.47	0.11	0.47	
GRMZM2G086237	5:23872218-23877754	4.34	n.s.	n.s.	n.s.	-1.77	-0.44	0.08	-0.37	
GRMZM2G154954	5:31444525-31445711	2.81	n.s.	n.s.	n.s.	-2.08	-0.77	0.26	-0.02	Glycine rich protein 3 [Source:Uniprot/SPTREMBL;Acc:Q2XQA4]
GRMZM2G326707	5:31880968-31883167	4.01	n.s.	n.s.	n.s.	-1.48	-0.44	-0.25	-0.56	Uncharacterized protein [Source:Uniprot/SPTREMBL;Acc:B4FSY5]
GRMZM2G418005	5:33582886-33584618	2.97	n.s.	n.s.	n.s.	2.13	0.32	0.20	1.88	
GRMZM2G042927	5:34305142-34308829	2.02	n.s.	n.s.	n.s.	1.61	1.27	-0.09	0.05	
GRMZM2G096228	5:37374780-37376902	2.81	n.s.	n.s.	n.s.	-3.64	-3.11	-0.21	-0.48	Caltractin [Source:Uniprot/SPTREMBL;Acc:B6TJ23]
GRMZM2G104484	5:46109709-46119468	1.89	n.s.	n.s.	n.s.	-4.06	-5	-5	-0.81	

GRMZM2G026980	5:53906405-53908269	2.52	n.s.	n.s.	n.s.	-2.31	-0.79	0.17	-0.20	Uncharacterized protein; Xyloglucan endo-transglycosylase homolog; Xyloglucan endotransglucosylase/hydrolase protein 23 [Source:Uniprot/SPTREMBL;Acc:Q42446]
GRMZM2G132331	5:62683002-62684543	3.52	3.76	n.s.	n.s.	3.09	4.82	0.15	-0.64	
GRMZM2G037284	5:63709506-63713912	3.78	n.s.	n.s.	n.s.	4.52	-0.27	0.19	5.41	Ser/Thr-rich protein T10 in DGCR region [Source:Uniprot/SPTREMBL;Acc:B6TDB2]
GRMZM2G089836	5:67508591-67511890	n.s.	n.s.	n.s.	6.53	-1.42	-0.61	-2.39	-2.61	Beta-fructofuranosidase 1; Invertase [Source:Uniprot/SPTREMBL;Acc:B6T0A9]
GRMZM2G029951	5:121407893-121413490	3.21	n.s.	n.s.	n.s.	1.10	0.22	0.14	0.45	Putative uncharacterized protein [Source:Uniprot/SPTREMBL;Acc:D2XBY1]
GRMZM2G098925	5:143009479-143010117	2.21	n.s.	n.s.	n.s.	-2.66	-0.37	0.66	-0.30	
GRMZM2G069542	5:144808459-144816059	10.23	n.s.	n.s.	n.s.	1.90	1.88	0.20	0.18	Phosphoenolpyruvate carboxylase [Source:Uniprot/SPTREMBL;Acc:Q9SAZ6]
GRMZM2G154942	5:146481853-146482978	n.s.	2.48	n.s.	n.s.	4.99	4.39	0.35	0.73	
GRMZM2G041980	5:148304652-148307020	9.41	n.s.	2.07	n.s.	3.46	-0.25	-2.41	0.81	Aquaporin NIP1-1 [Source:Uniprot/SWISSPROT;Acc:Q9ATN4]
GRMZM2G119623	5:153945529-153948183	2.77	n.s.	n.s.	n.s.	-1.49	-0.69	-0.16	-0.21	
GRMZM2G362312	5:154480775-154482181	5.90	n.s.	n.s.	n.s.	-1.95	-0.64	-0.17	-0.52	
GRMZM2G412604	5:158684738-158689453	2.17	n.s.	n.s.	n.s.	0.95	0.12	0.07	0.40	Uncharacterized protein [Source:Uniprot/SPTREMBL;Acc:B4F891]
GRMZM2G043279	5:163112214-163119453	3.57	n.s.	n.s.	n.s.	-1.66	-1.80	0.07	0.13	60S ribosomal protein L35; Uncharacterized protein [Source:Uniprot/SPTREMBL;Acc:B6SX73]
GRMZM2G012690	5:163209219-163228678	4.68	n.s.	n.s.	n.s.	1.71	1.56	-0.05	0.01	Uncharacterized protein [Source:Uniprot/SPTREMBL;Acc:B8A0D7]
GRMZM2G011627	5:163229122-163232017	1.72	n.s.	n.s.	n.s.	-1.53	-0.29	0.18	-0.02	Uncharacterized protein [Source:Uniprot/SPTREMBL;Acc:C4J649]
GRMZM2G307368	5:163739990-163741157	1.83	n.s.	n.s.	n.s.	-2.71	-2.83	-0.15	-0.10	Drought-induced protein 1; Uncharacterized protein [Source:Uniprot/SPTREMBL;Acc:B6UGD9]
GRMZM2G312281	5:164364956-164366457	1.98	n.s.	n.s.	n.s.	4.23	5	0.09	0	
GRMZM2G136296	5:167061159-167068865	5.63	n.s.	n.s.	n.s.	1.59	1.41	-0.21	-0.15	
GRMZM2G044788	5:167347515-167348479	2.41	n.s.	n.s.	n.s.	-4.65	-5	-0.73	-2.05	Putative uncharacterized protein [Source:Uniprot/SPTREMBL;Acc:B6SLC8]
GRMZM2G040602	5:180424999-180428892	n.s.	2.38	n.s.	n.s.	-3.70	-8.30	5	0.67	
GRMZM2G179294	5:180731982-180733210	3.90	n.s.	n.s.	n.s.	-1.28	-0.35	-0.27	-0.57	High affinity nitrate transporter [Source:Uniprot/SPTREMBL;Acc:Q0VH26]
GRMZM2G148385	5:185403879-185404382	6.20	n.s.	n.s.	n.s.	1.86	0.43	0.09	0.56	
GRMZM2G170692	5:186795549-186798126	4.70	n.s.	n.s.	n.s.	-1.80	-0.31	-0.22	-1.62	Phenylalanine ammonia-lyase [Source:Uniprot/SPTREMBL;Acc:C0HJ40]
GRMZM2G175177	5:187657033-187668727	4.04	n.s.	n.s.	n.s.	2.42	0.33	0.17	2.09	Replication factor A; Uncharacterized protein [Source:Uniprot/SPTREMBL;Acc:B6TFF7]

GRMZM2G056236	5:192167119-192169231	1.46	n.s.	n.s.	n.s.	-3.47	-1.57	-0.42	-0.59	Uncharacterized protein [Source:Uniprot/SPTREMBL;Acc:B4FW28]
GRMZM2G112247	5:193726749-193731587	4.59	n.s.	n.s.	n.s.	-4.38	-4.58	-0.03	0.03	Uncharacterized protein [Source:Uniprot/SPTREMBL;Acc:B4FTH4]
GRMZM2G021270	5:199862396-199869608	5.07	n.s.	n.s.	n.s.	1.77	1.64	0.02	0.05	Uncharacterized protein [Source:Uniprot/SPTREMBL;Acc:B4FGK4]
GRMZM2G161641	5:204062210-204065650	4.69	n.s.	n.s.	n.s.	-3.09	-3.03	-0.29	-0.33	
GRMZM5G872068	5:205237018-205240533	9.37	n.s.	n.s.	n.s.	-2.06	-1.29	-0.13	-0.49	Glutamine synthetase root isozyme 3 [Source:Uniprot/SWISSPROT;Acc:P38561]
GRMZM5G878558	5:210731863-210735309	20	n.s.	n.s.	n.s.	2.52	-0.57	-0.56	2.49	Uncharacterized protein [Source:Uniprot/SPTREMBL;Acc:C0PE76]
GRMZM2G100467	5:213819764-213820720	1.52	2.18	n.s.	n.s.	-1.46	-2.26	-0.25	0.14	60S ribosomal protein L39 [Source:Uniprot/SWISSPROT;Acc:P51425]
GRMZM5G836910	5:213904654-213909590	5.05	n.s.	n.s.	n.s.	-2.41	-0.74	0.16	-0.32	Uncharacterized protein [Source:Uniprot/SPTREMBL;Acc:C4J030]
GRMZM2G180430	5:214089780-214098979	1.93	n.s.	n.s.	n.s.	-1.80	-3.00	-0.71	-0.12	Uncharacterized protein [Source:Uniprot/SPTREMBL;Acc:B4F814]
GRMZM2G024303	6:2438892-2445703	4.18	n.s.	n.s.	n.s.	-1.53	-1.93	-0.23	-0.04	Uncharacterized protein [Source:Uniprot/SPTREMBL;Acc:K7U9G1]
GRMZM2G180659	6:6890037-6895101	2.59	n.s.	n.s.	n.s.	-1.85	-0.50	0.23	-0.20	LHT1; Uncharacterized protein [Source:Uniprot/SPTREMBL;Acc:B4FVN6]
GRMZM2G180625	6:6898694-6903246	2.17	n.s.	n.s.	n.s.	-1.11	-0.55	-0.13	-0.13	Glyceraldehyde-3-phosphate dehydrogenase 2, cytosolic [Source:Uniprot/SWISSPROT;Acc:Q09054]
GRMZM2G348325	6:15505528-15508780	3.14	n.s.	n.s.	n.s.	-6.74	-10	-5	-0.26	
GRMZM2G005622	6:21941042-21944318	1.71	n.s.	n.s.	n.s.	-1.87	-0.76	-0.19	-0.36	Uncharacterized protein [Source:Uniprot/SPTREMBL;Acc:B4FBP9]
GRMZM2G479746	6:32536837-32553298	1.57	n.s.	n.s.	n.s.	-3.25	-2.92	0.33	0.18	Uncharacterized protein [Source:Uniprot/SPTREMBL;Acc:B4FKD8]
GRMZM2G130173	6:38086164-38086854	n.s.	3.60	n.s.	n.s.	-1.72	-2.13	-0.01	0.19	Metallothionein-like protein type 2; Uncharacterized protein [Source:Uniprot/SPTREMBL;Acc:B6SP45]
GRMZM2G466498	6:65509408-65511415	n.s.	n.s.	n.s.	n.s.	-0.69	0	0	0	Uncharacterized protein [Source:Uniprot/SPTREMBL;Acc:K7VBU5]
GRMZM2G433801	6:80184235-80200723	2.67	n.s.	n.s.	n.s.	1.58	1.21	0.06	0.21	Uncharacterized protein [Source:Uniprot/SPTREMBL;Acc:K7UDH4]
GRMZM2G145518	6:82813530-82815309	4.13	3.97	n.s.	n.s.	4.03	2.97	0.05	0.62	Uncharacterized protein [Source:Uniprot/SPTREMBL;Acc:K7ULF7]
GRMZM2G349895	6:89883474-89885926	2.26	n.s.	n.s.	n.s.	1.16	0.16	0.05	0.45	Uncharacterized protein [Source:Uniprot/SPTREMBL;Acc:K7UM57]
GRMZM2G179638	6:105093952-105098839	2.70	n.s.	n.s.	n.s.	-1.95	-3.14	-0.63	-0.04	Uncharacterized protein [Source:Uniprot/SPTREMBL;Acc:C4J4J6]
GRMZM2G403029	6:105437098-105442762	1.99	n.s.	n.s.	n.s.	3.51	0.32	-0.43	0.58	
GRMZM2G373435	6:108074206-108076509	2.50	n.s.	n.s.	n.s.	-2.73	-0.69	0.14	-0.42	Uncharacterized protein [Source:Uniprot/SPTREMBL;Acc:K7UJC3]
GRMZM2G122892	6:114480445-114484114	2.30	n.s.	n.s.	n.s.	2.63	2.63	-0.13	-0.12	Uncharacterized protein [Source:Uniprot/SPTREMBL;Acc:K7UGT0]

GRMZM2G132373	6:117794563-117803763	5.13	n.s.	n.s.	n.s.	1.48	0.17	-0.39	0.16	Putative 1-phosphatidylinositol-4-phosphate 5-kinase/ zinc ion binding family [Source:Uniprot/SPTREMBL;Acc:K7UH43]
GRMZM2G383404	6:120018886-120020772	10.26	n.s.	n.s.	n.s.	-2.75	-1.02	-0.40	-0.76	Anthocyanidin 3-O-glucosyltransferase [Source:Uniprot/SPTREMBL;Acc:K7V1F2]
GRMZM2G142409	6:121943416-121946294	2.71	n.s.	n.s.	n.s.	-2.78	-0.86	0.57	0.04	Reticulon [Source:Uniprot/SPTREMBL;Acc:B6TG01]
GRMZM2G371793	6:129787843-129789773	3.06	2.30	n.s.	n.s.	1.71	2.49	-0.26	-0.62	Uncharacterized protein [Source:Uniprot/SPTREMBL;Acc:K7V2G3]
GRMZM2G441656	6:129879766-129880816	10.83	n.s.	n.s.	n.s.	-2.85	-3.14	0.20	0.34	Uncharacterized protein [Source:Uniprot/SPTREMBL;Acc:B6TFN4]
GRMZM2G383564	6:141670124-141673809	n.s.	n.s.	n.s.	n.s.	-0.97	0	0	0	Uncharacterized protein [Source:Uniprot/SPTREMBL;Acc:C0PN81]
GRMZM2G456568	6:147920772-147925808	2.17	n.s.	n.s.	n.s.	-1.31	-1.72	-0.35	-0.16	Putative NAC domain transcription factor superfamily protein isoform 1; Putative NAC domain transcription factor superfamily protein isoform 2 [Source:Uniprot/SPTREMBL;Acc:K7UT11]
GRMZM2G059285	6:151448309-151454115	5.34	2.31	n.s.	n.s.	-3.23	-3.33	-0.14	-0.10	Putative oxidoreductase, aldo/keto reductase family protein [Source:Uniprot/SPTREMBL;Acc:K7V540]
GRMZM2G143745	6:151870531-151873473	4.77	n.s.	n.s.	n.s.	1.75	0.44	0.00	0.39	Uncharacterized protein [Source:Uniprot/SPTREMBL;Acc:K7UKC6]
GRMZM5G849334	6:153224670-153228757	2.71	n.s.	n.s.	n.s.	-2.16	-0.94	-0.05	-0.20	Uncharacterized protein [Source:Uniprot/SPTREMBL;Acc:C4J8S7]
GRMZM2G141026	6:158688400-158689808	2.64	n.s.	n.s.	n.s.	-2.79	-1.16	-0.05	-0.29	Uncharacterized protein [Source:Uniprot/SPTREMBL;Acc:B4FRD8]
GRMZM2G069325	6:162990574-162992103	1.81	n.s.	n.s.	n.s.	-2.00	-0.83	-0.23	-0.36	Putative MYB DNA-binding domain superfamily protein; Uncharacterized protein [Source:Uniprot/SPTREMBL;Acc:B4FEC5]
GRMZM2G038284	6:164372183-164375563	4.30	n.s.	n.s.	n.s.	1.52	1.62	0.43	0.37	Fiber protein Fb2; Uncharacterized protein [Source:Uniprot/SPTREMBL;Acc:B4FJS2]
GRMZM2G135839	7:1192170-1200800	2.58	n.s.	n.s.	n.s.	-2.01	-0.89	-0.89	-2.05	
GRMZM2G117281	7:2741414-2742837	3.13	n.s.	n.s.	n.s.	-4.20	-4.07	0.53	0.46	Uncharacterized protein [Source:Uniprot/SPTREMBL;Acc:C0P8B3]
GRMZM2G465226	7:3483731-3484794	n.s.	12.43	5.06	n.s.	2.29	3.71	1.83	0.21	Uncharacterized protein [Source:Uniprot/SPTREMBL;Acc:B4FVP5]
GRMZM2G450866	7:3884719-3887155	20	n.s.	n.s.	n.s.	-4.78	-1.69	-0.42	-2.24	
GRMZM2G320298	7:7118086-7119389	1.61	n.s.	n.s.	n.s.	-3.01	-3.01	0.07	0.06	
GRMZM2G347270	7:7340994-7348207	2.58	n.s.	n.s.	n.s.	5.31	5.31	-0.11	-0.46	
GRMZM2G443668	7:7495433-7502357	2.89	n.s.	n.s.	n.s.	-1.37	-0.51	-0.10	-0.29	
GRMZM2G158489	7:13610538-13613654	4.24	n.s.	n.s.	n.s.	-1.84	-0.74	-0.05	-0.23	Uncharacterized protein [Source:Uniprot/SPTREMBL;Acc:C0P9T2]
GRMZM2G088469	7:17179983-17182140	3.36	n.s.	n.s.	n.s.	-2.71	-2.20	-0.33	-0.60	Putative uncharacterized protein [Source:Uniprot/SPTREMBL;Acc:B6TKI8]

GRMZM2G092137	7:83764957-83767371	4.13	n.s.	n.s.	n.s.	2.23	1.08	-0.37	0.16	Uncharacterized protein [Source:Uniprot/SPTREMBL;Acc:C0PFI4]
GRMZM2G354621	7:95428215-95431857	n.s.	n.s.	n.s.	n.s.	-1.52	-1.14	0	0	Uncharacterized protein [Source:Uniprot/SPTREMBL;Acc:B4FMA1]
GRMZM2G354615	7:95446340-95451284	n.s.	n.s.	n.s.	n.s.	-6.74	-5.14	0	0	
GRMZM2G060369	7:118015999-118018602	4.60	n.s.	n.s.	n.s.	-2.87	-3.69	-0.90	-0.50	S-adenosylmethionine decarboxylase proenzyme [Source:Uniprot/SPTREMBL;Acc:B8A123]
GRMZM2G461159	7:118074243-118077015	9.24	2.11	n.s.	n.s.	-3.46	-4.17	-0.77	-0.42	S-adenosylmethionine decarboxylase proenzyme [Source:Uniprot/SPTREMBL;Acc:B6TCF0]
GRMZM2G052667	7:120351650-120354724	8.16	n.s.	n.s.	n.s.	-3.23	-2.27	0.61	0	Ethylene response factor [Source:Uniprot/SPTREMBL;Acc:B6TS80]
GRMZM2G099049	7:120764735-120766193	n.s.	n.s.	n.s.	1.52	-1.54	0.49	-0.01	-2.60	
GRMZM2G140342	7:121316023-121319306	3.99	n.s.	n.s.	n.s.	-3.25	-3.30	-0.35	-0.33	TMEM87A protein; Uncharacterized protein [Source:Uniprot/SPTREMBL;Acc:B4F8U5]
GRMZM2G118119	7:121710274-121712960	1.89	n.s.	n.s.	n.s.	-1.53	-2.15	-0.53	-0.17	Uncharacterized protein [Source:Uniprot/SPTREMBL;Acc:K7UXC8]
GRMZM2G176430	7:132515986-132518574	n.s.	4.39	n.s.	n.s.	-5	-5.41	5	3.24	Uncharacterized protein [Source:Uniprot/SPTREMBL;Acc:C0PFM2]
GRMZM2G016836	7:133622968-133624950	4.25	n.s.	n.s.	n.s.	-6.20	-3.64	-0.60	-0.07	Dihydroflavonol-4-reductase [Source:Uniprot/SPTREMBL;Acc:B6TR31]
GRMZM2G004990	7:134079460-134083853	1.86	n.s.	n.s.	n.s.	-2.17	-1.02	-0.26	-0.20	Uncharacterized protein [Source:Uniprot/SPTREMBL;Acc:C0HEF4]
GRMZM2G131281	7:135591965-135593007	1.41	n.s.	n.s.	n.s.	-3.58	-0.75	0.24	-0.78	
GRMZM2G430052	7:136142239-136143040	4.25	n.s.	n.s.	n.s.	-2.43	-0.75	-0.28	-0.76	Uncharacterized protein [Source:Uniprot/SPTREMBL;Acc:C0P725]
GRMZM2G109448	7:143296603-143298282	1.69	n.s.	n.s.	n.s.	-6.91	-10	-5	0.29	Histone H2A [Source:Uniprot/SPTREMBL;Acc:B6T101]
GRMZM2G033634	7:146486588-146490051	1.37	n.s.	n.s.	n.s.	1.47	-0.04	0.07	1.58	Uncharacterized protein [Source:Uniprot/SPTREMBL;Acc:B4F8F0]
GRMZM2G134711	7:149945733-149948087	4.93	3.34	n.s.	n.s.	3.33	4.53	-0.16	-0.77	Putative uncharacterized protein [Source:Uniprot/SPTREMBL;Acc:B6U1C5]
GRMZM2G108849	7:152772008-152776320	3.57	n.s.	n.s.	n.s.	-1.37	-1.64	-0.17	-0.05	Cathepsin B-like cysteine proteinase 3 [Source:Uniprot/SPTREMBL;Acc:B6TLR9]
GRMZM2G075826	7:153248972-153249284	8.70	10.65	n.s.	n.s.	-2.95	-2.73	0.12	0.00	
GRMZM2G157505	7:164456635-164457479	2.75	1.94	n.s.	n.s.	-2.34	-3.81	-0.63	0.09	EGG APPARATUS-1 protein [Source:Uniprot/SPTREMBL;Acc:B6TF57]
GRMZM2G089736	7:165456773-165459269	2.19	n.s.	n.s.	n.s.	-1.53	-0.96	-0.42	-0.24	Uncharacterized protein [Source:Uniprot/SPTREMBL;Acc:B4FQE1]
GRMZM2G477325	7:168401561-168403148	20	n.s.	n.s.	n.s.	-2.98	-2.96	-0.50	-0.52	Hydrophobic protein LTI6A; Uncharacterized protein [Source:Uniprot/SPTREMBL;Acc:B4FFP9]
GRMZM2G039757	7:168744977-168747272	3.87	n.s.	n.s.	n.s.	-1.74	-0.99	-0.61	-0.50	
GRMZM2G136429	7:170855214-170865748	n.s.	n.s.	n.s.	1.74	2.19	0.51	2.31	3.47	

GRMZM2G060190	8:4593244-4596043	1.89	n.s.	n.s.	n.s.	-1.53	-2.15	-0.53	-0.17	Uncharacterized protein [Source:Uniprot/SPTREMBL;Acc:K7UXC8]
GRMZM2G058681	8:6243295-6256960	n.s.	5.52	n.s.	n.s.	2.96	3.38	0.25	0.03	Uncharacterized protein [Source:Uniprot/SPTREMBL;Acc:K7UPC5]
GRMZM2G077914	8:9626905-9629545	6.57	3.18	n.s.	n.s.	-3.46	-3.27	0.18	0.07	Uncharacterized protein [Source:Uniprot/SPTREMBL;Acc:B4FE27]
GRMZM2G336694	8:10989824-10990619	20	9.01	n.s.	n.s.	3.67	3.38	-0.24	-0.08	Uncharacterized protein [Source:Uniprot/SPTREMBL;Acc:K7UY89]
GRMZM5G876768	8:12713843-12717640	1.92	n.s.	n.s.	n.s.	-2.58	-0.87	0.05	-0.38	Uncharacterized protein [Source:Uniprot/SPTREMBL;Acc:B4FSX0]
GRMZM2G052869	8:14541174-14542451	5.25	5.23	n.s.	n.s.	-3.30	-4.25	-0.21	0.26	Metallothionein-like protein type 2; Uncharacterized protein [Source:Uniprot/SPTREMBL;Acc:B4FT00]
GRMZM2G000489	8:14933362-14934823	1.33	n.s.	n.s.	n.s.	-3.63	-1.86	-0.48	-0.43	Gibberellin receptor GID1L2; Uncharacterized protein [Source:Uniprot/SPTREMBL;Acc:B4FJV4]
GRMZM2G107639	8:18231012-18236007	n.s.	n.s.	n.s.	n.s.	2.70	3.52	0	0	Transaminase/ transferase isoform 1; Transaminase/ transferase isoform 2; Transaminase/ transferase, transferring nitrogenous groups [Source:Uniprot/SPTREMBL;Acc:B6TMW7]
GRMZM2G047139	8:24719638-24722490	3.57	n.s.	n.s.	n.s.	-1.55	-0.19	0.15	-0.44	Esterase; Uncharacterized protein [Source:Uniprot/SPTREMBL;Acc:B6TZ91]
GRMZM2G154278	8:26066129-26069719	4.30	n.s.	n.s.	n.s.	-4.33	-3.84	0.21	-0.05	Pre-mRNA-splicing factor cwc15 [Source:Uniprot/SPTREMBL;Acc:B6T6R6]
GRMZM2G123143	8:40678211-40682507	2.19	n.s.	n.s.	n.s.	-3.21	-0.51	0.32	-0.78	Uncharacterized protein [Source:Uniprot/SPTREMBL;Acc:K7VC64]
GRMZM2G061052	8:43287734-43288541	2.05	n.s.	n.s.	n.s.	1.72	0.10	0.13	1.68	Putative HLH DNA-binding domain superfamily protein; Uncharacterized protein [Source:Uniprot/SPTREMBL;Acc:B4FNT9]
GRMZM2G031827	8:70136196-70144793	2.51	n.s.	n.s.	n.s.	3.36	2.30	-0.19	0.34	Splicing factor U2af subunit isoform 1; Splicing factor U2af subunit isoform 2; Splicing factor U2af subunit isoform 3; Splicing factor U2af subunit isoform 4; Splicing factor U2af subunit isoform 5 [Source:Uniprot/SPTREMBL;Acc:K7V1E2]
GRMZM2G153977	8:70995815-70998984	20	10.35	n.s.	n.s.	-4.09	-4.24	-0.13	-0.06	Uncharacterized protein [Source:Uniprot/SPTREMBL;Acc:K7UXH3]
GRMZM2G077546	8:71383585-71391003	1.85	n.s.	n.s.	n.s.	-3.50	-4.20	0.01	0.40	Uncharacterized protein [Source:Uniprot/SPTREMBL;Acc:K7V1G3]
GRMZM2G053322	8:90964172-90967651	4.75	n.s.	n.s.	n.s.	-1.80	-0.38	-0.09	-0.62	UDP-sulfoquinovose synthase; Uncharacterized protein [Source:Uniprot/SPTREMBL;Acc:C4JAX7]
GRMZM2G050625	8:97273628-97276763	1.74	n.s.	n.s.	n.s.	5.56	5.54	-0.06	-0.07	Uncharacterized protein [Source:Uniprot/SPTREMBL;Acc:K7UV43]
GRMZM2G096029	8:104279085-104281118	n.s.	n.s.	n.s.	3.30	-7.18	-5	-5	-2.91	Putative cytochrome P450 superfamily protein [Source:Uniprot/SPTREMBL;Acc:K7V3H4]

GRMZM2G082792	8:121883070-121887432	1.54	n.s.	n.s.	n.s.	-4.36	-4.01	0.11	0	Uncharacterized protein [Source:Uniprot/SPTREMBL;Acc:K7UX34]
GRMZM5G811797	8:122951332-122954098	4.18	n.s.	n.s.	n.s.	-1.83	-2.08	-0.11	0.01	Uncharacterized protein [Source:Uniprot/SPTREMBL;Acc:B4F9I6]
GRMZM5G855375	8:125007016-125008185	4.20	4.05	n.s.	n.s.	-4.09	-3.44	0.65	0.32	Uncharacterized protein [Source:Uniprot/SPTREMBL;Acc:K7UXJ6]
GRMZM2G117198	8:129620162-129623583	1.72	n.s.	n.s.	n.s.	0.96	0.06	0.16	0.56	S-adenosylmethionine synthase [Source:Uniprot/SPTREMBL;Acc:B4FAD1]
GRMZM2G064758	8:131213666-131217503	1.71	n.s.	n.s.	n.s.	-5.29	-4.93	0.57	0.38	
GRMZM2G147714	8:144467962-144472082	11.02	n.s.	n.s.	4.29	2.60	0.40	0.18	2.14	Uncharacterized protein [Source:Uniprot/SPTREMBL;Acc:K7VZH5]
GRMZM2G021436	8:145683890-145686249	4.58	n.s.	n.s.	n.s.	-1.94	-0.62	-0.06	-0.42	Putative cytochrome P450 superfamily protein [Source:Uniprot/SPTREMBL;Acc:K7V7F4]
GRMZM2G171279	8:147093256-147099052	3.20	n.s.	n.s.	n.s.	1.54	0.22	-0.34	0.14	Uncharacterized protein [Source:Uniprot/SPTREMBL;Acc:K7V3Z4]
GRMZM2G585314	8:162746940-162747070	2.68	1.58	n.s.	n.s.	3.27	3.27	-0.34	-0.33	
GRMZM2G476762	8:163031387-163032159	3.92	5.61	n.s.	n.s.	-1.55	-2.60	-0.18	0.34	Uncharacterized protein [Source:Uniprot/SPTREMBL;Acc:B4F9G3]
GRMZM2G519229	8:163382028-163384441	4.16	4.50	n.s.	n.s.	-8.66	-7.82	0.21	-0.22	
GRMZM2G005236	8:165542367-165548286	1.91	n.s.	n.s.	n.s.	-1.47	-0.52	-0.10	-0.32	Uncharacterized protein [Source:Uniprot/SPTREMBL;Acc:K7V6V5]
GRMZM2G149317	8:168270604-168276092	1.45	n.s.	n.s.	n.s.	-2.79	-0.90	-0.47	-1.95	Phytoene synthase 2 isoform 1; Phytoene synthase 2 isoform 2 [Source:Uniprot/SPTREMBL;Acc:K7V3J4]
GRMZM2G135586	8:173788082-173793095	5.36	n.s.	n.s.	n.s.	-2.79	-0.80	-0.28	-1.77	Uncharacterized protein [Source:Uniprot/SPTREMBL;Acc:C0PMP2]
GRMZM2G027392	9:1427342-1428678	6.57	n.s.	n.s.	n.s.	-1.74	-0.59	0.16	-0.10	B12D protein [Source:Uniprot/SPTREMBL;Acc:B6T1H8]
GRMZM2G047181	9:14149995-14152647	2.26	n.s.	n.s.	n.s.	-5.33	-5.34	-0.07	-0.07	Putative uncharacterized protein [Source:Uniprot/SPTREMBL;Acc:B6UGF2]
GRMZM2G046669	9:14200644-14202039	8.42	1.38	n.s.	n.s.	-5.73	-6.57	-1.00	-0.59	Uncharacterized protein [Source:Uniprot/SPTREMBL;Acc:K7VRH8]
GRMZM2G033208	9:22779372-22783918	2.59	n.s.	n.s.	n.s.	1.23	0.53	0.05	0.12	Transketolase, chloroplastic [Source:Uniprot/SWISSPROT;Acc:Q7SIC9]
GRMZM2G382673	9:44374789-44379966	1.92	n.s.	n.s.	n.s.	-2.35	-2.54	-0.15	-0.06	Thioredoxin-like protein 5; Uncharacterized protein [Source:Uniprot/SPTREMBL;Acc:B8A1M2]
GRMZM2G049811	9:48437558-48441823	9.05	n.s.	n.s.	6.06	1.78	0.05	0.25	2.13	Formate dehydrogenase; Uncharacterized protein [Source:Uniprot/SPTREMBL;Acc:C0P848]
GRMZM2G376416	9:58140574-58143428	1.49	n.s.	n.s.	n.s.	-3.07	-1.53	-0.48	-0.49	Uncharacterized protein [Source:Uniprot/SPTREMBL;Acc:K7V996]
GRMZM2G124872	9:60085372-60088710	4.41	n.s.	n.s.	n.s.	-2.03	-0.44	0.04	-0.54	Growth regulator; Uncharacterized protein [Source:Uniprot/SPTREMBL;Acc:B4FG68]
GRMZM5G812126	9:62776697-62777760	2.16	n.s.	n.s.	n.s.	-2.63	-1.30	-0.48	-0.50	Uncharacterized protein [Source:Uniprot/SPTREMBL;Acc:B6UCG7]

GRMZM2G407837	9:72844968-72848373	2.83	n.s.	n.s.	n.s.	-1.10	-0.70	-0.07	0.07	Cytochrome c biogenesis C [Source:Uniprot/SPTREMBL;Acc:Q0GR56]
GRMZM2G017959	9:82088507-82096022	1.38	n.s.	n.s.	n.s.	1.96	0.28	0.13	1.63	
GRMZM2G026143	9:88242514-88243287	2.85	n.s.	n.s.	n.s.	-4.22	-10	-5	-0.44	Uncharacterized protein [Source:Uniprot/SPTREMBL;Acc:K7VAJ0]
GRMZM2G099642	9:88874335-88876199	2.74	n.s.	n.s.	n.s.	-2.77	-0.12	0.01	-2.52	Putative alcohol dehydrogenase superfamily protein [Source:Uniprot/SPTREMBL;Acc:K7VVX9]
GRMZM2G152126	9:102961672-102963569	3.11	n.s.	n.s.	n.s.	-1.88	-0.97	0.04	0.06	Uncharacterized protein [Source:Uniprot/SPTREMBL;Acc:K7VFW8]
GRMZM2G143311	9:114763115-114764657	4.27	7.59	n.s.	n.s.	5.94	3.90	0.03	1.04	
GRMZM2G000209	9:120228024-120230405	1.64	n.s.	n.s.	n.s.	-2.84	-1.30	0.03	-0.10	Uncharacterized protein [Source:Uniprot/SPTREMBL;Acc:K7VHB0]
GRMZM2G132238	9:120365363-120366799	1.70	n.s.	n.s.	n.s.	3.58	0.94	-0.12	0.73	Putative metacaspase family protein [Source:Uniprot/SPTREMBL;Acc:K7VKS2]
GRMZM2G150242	9:121717576-121721740	5.29	n.s.	n.s.	n.s.	-2.16	-0.73	-0.13	-0.39	Putative DUF827 domain containing family protein [Source:Uniprot/SPTREMBL;Acc:K7VHH9]
GRMZM2G047546	9:125052702-125059364	1.99	n.s.	n.s.	n.s.	-4.31	-5.24	-0.19	0.27	Uncharacterized protein [Source:Uniprot/SPTREMBL;Acc:K7VDA8]
GRMZM2G157683	9:128089496-128090507	2.10	n.s.	n.s.	n.s.	-4.42	-10	-5	-0.45	Uncharacterized protein [Source:Uniprot/SPTREMBL;Acc:B4FV07]
GRMZM2G157760	9:128121879-128127627	2.14	n.s.	n.s.	n.s.	-5.51	-3.42	0.61	-0.59	Uncharacterized protein [Source:Uniprot/SPTREMBL;Acc:B6TIP8]
GRMZM5G872256	9:133899222-133900997	4.64	n.s.	n.s.	n.s.	-2.58	-5.12	-1.36	-0.10	Galactinol synthase 3 [Source:Uniprot/SPTREMBL;Acc:K7WDW6]
GRMZM2G385200	9:135550860-135552129	n.s.	n.s.	n.s.	2.41	0.00	-0.62	0.26	1.78	ZmNAS1 protein [Source:Uniprot/SPTREMBL;Acc:K7VEJ7]
GRMZM2G312481	9:135720513-135721787	20	n.s.	n.s.	n.s.	2.58	0	0.32	1.75	Uncharacterized protein [Source:Uniprot/SPTREMBL;Acc:K7WE51]
GRMZM2G034956	9:135796453-135797695	n.s.	n.s.	n.s.	4.29	0.04	-0.67	0.23	1.87	ZmNAS1 protein [Source:Uniprot/SPTREMBL;Acc:K7VEJ7]
GRMZM2G147787	9:143190120-143191677	3.36	n.s.	n.s.	n.s.	-2.48	-2.40	-0.14	0	Uncharacterized protein [Source:Uniprot/SPTREMBL;Acc:K7W1D8]
GRMZM2G046601	9:146062601-146071470	8.48	n.s.	n.s.	n.s.	3.67	2.11	-0.03	0.69	Glutamine synthetase3 isoform 1; Glutamine synthetase3 isoform 2 [Source:Uniprot/SPTREMBL;Acc:K7VKG2]
GRMZM2G154149	9:147664616-147669617	n.s.	1.92	n.s.	n.s.	-2.94	-3.54	0.03	0.38	Putative phototropic-resoponsive NPH3 family protein isoform 1; Putative phototropic-resoponsive NPH3 family protein isoform 2; Putative phototropic-resoponsive NPH3 family protein isoform 3; Uncharacterized protein [Source:Uniprot/SPTREMBL;Acc:C0PCF1]
GRMZM2G181551	9:148225278-148226869	2.70	1.65	n.s.	n.s.	-1.77	-2.62	-0.56	-0.12	Uncharacterized protein [Source:Uniprot/SPTREMBL;Acc:B4FDZ1]

GRMZM2G057491	9:152081189-152085513	2.41	n.s.	n.s.	n.s.	-5.77	-6.06	-0.64	-0.21	Glutamyl-tRNA synthetase, cytoplasmic [Source:Uniprot/SPTREMBL;Acc:K7VHH0]
GRMZM2G075417	9:153426402-153428332	1.32	n.s.	n.s.	n.s.	3.78	3.06	0.34	-0.84	Putative AMP-dependent synthetase and ligase superfamily protein [Source:Uniprot/SPTREMBL;Acc:K7VHQ0]
GRMZM2G135536	10:893975-895713	20	20	n.s.	n.s.	3.51	4.24	0.15	-0.21	Putative cytochrome P450 superfamily protein; Uncharacterized protein [Source:Uniprot/SPTREMBL;Acc:C0PH19]
GRMZM2G152016	10:2455669-2456543	3.26	2.57	n.s.	n.s.	-2.82	-2.74	0.32	0.27	Uncharacterized protein [Source:Uniprot/SPTREMBL;Acc:K7TE51]
GRMZM2G356817	10:3160479-3170525	1.96	n.s.	n.s.	n.s.	-6.81	-4.91	0	0	Uncharacterized protein [Source:Uniprot/SPTREMBL;Acc:K7TIA6]
GRMZM2G356839	10:3166897-3170525	n.s.	n.s.	n.s.	n.s.	-1.82	0	0	0	Uncharacterized protein [Source:Uniprot/SPTREMBL;Acc:B6SH77]
GRMZM5G879178	10:3442109-3446793	4.78	n.s.	n.s.	n.s.	-1.78	-0.91	-0.13	-0.12	Disease resistance analog PIC20 [Source:Uniprot/SPTREMBL;Acc:K7TEA5]
GRMZM2G443939	10:3698043-3703030	5.11	n.s.	n.s.	n.s.	-2.81	-2.75	-0.18	-0.22	Uncharacterized protein [Source:Uniprot/SPTREMBL;Acc:K7TIC9]
GRMZM2G022318	10:4611628-4615022	1.98	n.s.	n.s.	n.s.	-1.56	-0.32	0.18	-0.29	Putative heavy metal transport/detoxification superfamily protein [Source:Uniprot/SPTREMBL;Acc:K7TEH9]
GRMZM2G136910	10:8848145-8849700	4.07	n.s.	n.s.	n.s.	-5.04	-3.64	-0.16	-0.72	Abscisic stress ripening protein 2; Dip protein; Uncharacterized protein [Source:Uniprot/SPTREMBL;Acc:B4FKG5]
GRMZM2G136981	10:8848145-8849700	8.93	n.s.	n.s.	n.s.	-5.96	-3.75	0.24	-0.88	Uncharacterized protein [Source:Uniprot/SPTREMBL;Acc:C0P4Z0]
GRMZM2G083347	10:14444467-14446346	3.36	2.15	n.s.	n.s.	-1.90	-2.06	0.05	0.18	Putative NAC domain transcription factor superfamily protein [Source:Uniprot/SPTREMBL;Acc:K7TJD6]
GRMZM2G458581	10:26648579-26650980	2.82	n.s.	n.s.	n.s.	-2.13	-2.31	-0.22	-0.14	Uncharacterized protein [Source:Uniprot/SPTREMBL;Acc:C4J3E2]
GRMZM2G316665	10:26715636-26718335	2.50	n.s.	n.s.	n.s.	-2.09	-2.43	-0.29	-0.13	Uncharacterized protein [Source:Uniprot/SPTREMBL;Acc:K7TFW8]
GRMZM2G325643	10:34497195-34506565	1.67	n.s.	n.s.	n.s.	1.59	1.33	0.02	0.08	Uncharacterized protein [Source:Uniprot/SPTREMBL;Acc:K7TK85]
GRMZM2G112782	10:71050577-71054403	2.95	n.s.	n.s.	n.s.	-2.82	-2.10	0	-0.31	Uncharacterized protein [Source:Uniprot/SPTREMBL;Acc:B4FNY9]
GRMZM2G333183	10:80568603-80577708	10.28	n.s.	n.s.	n.s.	-4.90	-1.38	-0.12	-2.39	Uncharacterized protein [Source:Uniprot/SPTREMBL;Acc:K7UFP0]
GRMZM2G123029	10:87089586-87092722	5.94	n.s.	n.s.	n.s.	-2.78	-2.09	0.14	-0.21	Uncharacterized protein [Source:Uniprot/SPTREMBL;Acc:B8A2B0]
GRMZM5G807276	10:90929577-90933166	3.86	n.s.	n.s.	n.s.	-3.89	-1.55	-0.98	-2.77	Flavonol synthase-like protein [Source:Uniprot/SPTREMBL;Acc:B6UED1]
GRMZM2G177077	10:99364725-99371326	12.45	n.s.	n.s.	n.s.	2.07	0.34	-0.23	0.44	Glucose-6-phosphate 1-dehydrogenase [Source:Uniprot/SPTREMBL;Acc:K7U267]
GRMZM2G119116	10:99602789-99608241	4.62	n.s.	n.s.	n.s.	-3.73	-14.06	-7.24	0.02	Uncharacterized protein [Source:Uniprot/SPTREMBL;Acc:B4FRE2]
GRMZM2G133721	10:111856375-111861824	2.73	4.22	n.s.	n.s.	7.65	6.36	-0.13	0.50	

GRMZM2G052100	10:116057033-116058081	3.27	n.s.	n.s.	n.s.	-4.09	-3.78	-0.07	-0.23	Asr protein; Uncharacterized protein [Source:Uniprot/SPTREMBL;Acc:A8IK79]
GRMZM2G332918	10:117676392-117677525	n.s.	1.65	n.s.	n.s.	8.42	9.11	-0.08	-0.44	Uncharacterized protein [Source:Uniprot/SPTREMBL;Acc:K7UIK7]
GRMZM2G135470	10:123373487-123378767	2.75	n.s.	n.s.	n.s.	1.45	0.37	-0.31	0.01	Aminoaldehyde dehydrogenase 2; Uncharacterized protein [Source:Uniprot/SPTREMBL;Acc:B4G044]
GRMZM2G064159	10:123811072-123815007	3.01	n.s.	n.s.	n.s.	-1.82	-1.63	0.09	0.06	Agmatine deiminase; Uncharacterized protein [Source:Uniprot/SPTREMBL;Acc:C0PHP8]
GRMZM2G540129	10:133711324-133711497	3.72	n.s.	n.s.	n.s.	-1.78	-0.73	-0.12	-0.29	
GRMZM5G843302	10:138661482-138664149	8.88	n.s.	n.s.	n.s.	-3.37	-2.15	-0.26	-1.76	Putative glycerol 3-phosphate permease; Uncharacterized protein [Source:Uniprot/SPTREMBL;Acc:B8A0W6]
GRMZM2G391272	10:138831641-138856323	n.s.	1.43	n.s.	n.s.	-1.02	-3.80	-2.32	0.01	Cortical cell-delineating protein [Source:Uniprot/SPTREMBL;Acc:B6UGA2]
GRMZM2G391286	10:138831641-138856323	10.45	7.96	n.s.	n.s.	-2.84	-3.92	-1.25	-0.09	Cortical cell-delineating protein [Source:Uniprot/SPTREMBL;Acc:B6U3L7]
GRMZM2G019986	10:142070802-142075281	4.63	n.s.	n.s.	n.s.	-1.72	-1.73	-0.26	-0.26	UDP-N-acetylglucosamine pyrophosphorylase; Uncharacterized protein [Source:Uniprot/SPTREMBL;Acc:B4FRH7]
GRMZM2G119802	10:142091580-142095485	2.00	1.33	n.s.	n.s.	-4.81	-4.92	0.29	0.32	Uncharacterized protein [Source:Uniprot/SPTREMBL;Acc:C0PK13]
GRMZM2G033668	10:142363305-142363715	7.67	n.s.	n.s.	n.s.	-1.86	-1.99	-0.03	0.02	
GRMZM2G589269	10:144064131-144064725	5.63	n.s.	n.s.	n.s.	-8.47	-10	-5	0.06	
GRMZM2G443833	10:144442269-144442574	2.23	n.s.	n.s.	n.s.	2.52	1.68	-0.15	0.46	
GRMZM2G445575	10:144522745-144530387	4.30	n.s.	n.s.	n.s.	-2.15	-0.53	-0.12	-0.52	Putative bZIP transcription factor superfamily protein isoform 1; Putative bZIP transcription factor superfamily protein isoform 2; Uncharacterized protein [Source:Uniprot/SPTREMBL;Acc:B4FAZ4]
GRMZM2G136964	10:144678705-144679659	2.91	n.s.	n.s.	n.s.	-2.93	-0.99	0.19	-0.29	Uncharacterized protein [Source:Uniprot/SPTREMBL;Acc:B6SIW9]
GRMZM2G098290	10:146465597-146471079	4.83	n.s.	n.s.	n.s.	1.83	0.43	-0.14	0.34	Glutamine synthetase, chloroplastic [Source:Uniprot/SWISSPROT;Acc:P25462]
GRMZM2G428119	10:146525000-146526364	3.36	n.s.	n.s.	n.s.	2.48	0.11	-0.07	0.97	Uncharacterized protein [Source:Uniprot/SPTREMBL;Acc:K7TT54]
GRMZM2G464137	10:147402070-147405547	8.41	n.s.	n.s.	n.s.	1.72	0.25	0.10	1.37	Methylthioribose kinase [Source:Uniprot/SPTREMBL;Acc:B6TDT6]

[1] Median $-\log_{10}$(P-value) for the corresponding transcript in specific comparison (Comp) as determined by all statistical approaches. n.s. = not significant in specific comparison

[2] Median log2FC for the corresponding transcript in specific comparison (Comp) as determined by all statistical approaches (positive log2FCs reflect higher expression values in Condition2 whilst negative log2FC refer to higher expression in Condition 1 of specific comparisons cf Table 1)

[3] All annotations for Function and Accessions from www.maizegdb.org

Supplemental Table 4 Candidate gene primer information.

Gene	Position	Function	Primer[a]	Reference
GRMZM2G159632	1:24404290-24408869	Sulfate Transporter	fw 5´-CCAGGGGTGCTGATTGTTAG-3´<br>rv 5´-GAGCTTTCGCCAACTCCTC-3´	[1) / 2)]
GRMZM2G060952	1:31744783-31747784	2'-deoxymugineicacid synthase	fw 5´-CTTCACGCCCGAGGACTT-3´<br>rv 5´-ATGGTGGCGAAGGAGAGC-3´	[1) / 2)]
GRMZM2G032182	1:46119595-46122942	Mitochondrial iron transporter 1	fw 5´-ACGCCCTTCGATGTTGTC-3´<br>rv 5´-TGAGCCCAGAATACCCATCT-3´	[1) / 2)]
GRMZM2G350312	1:65657063-65660290	bHLH Transcription Factor	fw 5´-GACAGAACAATGGGAAAGCAT-3´<br>rv 5´-TCAACTCGTTTTGAAGCTCTGA-3´	This study
GRMZM2G171111	1:194210583-194219751	methylthioadenosine / S-adenosyl homocysteine nucleosidase	fw 5´-CGACTGGGGATTCTCTGGATA-3´<br>rv 5´-GTCACGGCAATCAGGTTCTG-3´	[1) / 2)]
GRMZM2G106980	1:203647045-203653762	Putative BURP-domain protein	fw 5´-ACGTACCCCTACGTTGTGTTCT-3´<br>rv 5´-CAGACGATGCTGAGCTTAGAGA-3´	This study
GRMZM2G059465	1:209119390-209120903	O-methyl transferase	fw 5´-ACAGGCACAGACGAGACTGA-3´<br>rv 5´-GCGTCCTTGAACCACTCTTC-3´	This study
GRMZM2G478568	1:259776858-259778542	Nicotianamine Synthase 3	fw 5´-GTCATGGGCAAGGAGGAG-3´<br>rv 5´-ACGAGGTCGGTGAAGAGC-3´	[1) / 2)]
GRMZM2G132678	3:20795800-20806607	hydroxycinnamoyl-CoA shikimate/quinate hydroxycinnamoyl transferase	fw 5´-ACCTCTCCCGAGCCACTACT-3´<br>rv 5´-TGCCCAGCCAGCTAATAATC-3´	This study
GRMZM2G057413	3:148031451-148062994	Putative bHLH Transcription Factor	fw 5´-CAACACCAGCGAGATCGTAA-3´<br>rv 5´-GTCTTGTTCCCAAAGGTGGA-3´	This study
GRMZM2G057506	3:148063729-148064985	Putative bHLH Transcription Factor	fw 5´-GCCGACGCCTTCTTCATC-3´<br>rv 5´-AGGCTGCTGCTTCTTTCTGC-3´	This study
GRMZM2G400602	3:174361241-174363882	Putative Oligopeptide Transporter	fw 5´-GAAGAAGTGAAAGGCGTGCT-3´<br>rv 5´-ATGATGGTCACGCTGATGAA-3´	This study
GRMZM5G896496	3:211872426-211879044	Putative chaperone ClpC1	fw 5´-TTCTGCCTGACAAAGCCATT-3´<br>rv 5´-AAATCTTGGCCACGTACAGC-3´	This study
GRMZM2G139533	4:12777382-12783007	eukaryotic initiation factor 2B-like methylthioribose-1-phosphate isomerase	fw 5´-GCACATACAACCTCGCCATC-3´<br>rv 5´-CTTGCTTTCCCAGACCACCT-3´	[1) / 2)]
GRMZM2G574782	4:18676198-18682894	putative bifunctional methylribose-1-phosphate dehydratase/enolase phosphatase E1	fw 5´-CGAAAGTTCCTGTGTGGGTATT-3´<br>rv 5´-GGAAACCATGATTGTCAGGAAG-3´	This study/ [1) / 2)]
GRMZM2G020801	4:28118892-28124783	Aconitase	fw 5´-CTGCTAAGGGTCCGATGCT-3´<br>rv 5´-TGTACCGCTCATGTCCAGTG-3´	[1) / 2)]
GRMZM2G325575	4:183588190-183591209	Ferritin	fw 5´-CGCCTTTAACCGAGTTTGAC-3´<br>rv 5´-TTTATGGCTTCCACCTGCTC-3´	[1) / 2)]
GRMZM2G125596	4:195381313-195385012	iron deficiency responsive element-binding factor 1	fw 5´-CCCGGATAAGGATTCAGGTT-3´<br>rv 5´-CTCGGGTTCTTTGGCAGATA-3´	[1) / 2)]
GRMZM2G096958	4:219581607-219585123	Nicotinamine aminotransferase	fw 5´- GCCATTGAGGTTGTCGTCTC-3´<br>rv 5´- CCATTGCGGTGGTGTTCT-3´	This study/ [1) / 2)]
GRMZM2G010280	4:240232549-240234575	Putative nitrate transporter	fw 5´-TTTACCTCCTCGACCTACTCCA-3´<br>rv 5´-TATGGAGTCCCTTGCTCTTCTC-3´	This study
GRMZM2G010251	4:240243499-240245403	Putative nitrate transporter	fw 5´-GTGTTCTGCATGTCGCTCAT-3´<br>rv 5´-CTGCTGAACATGGTGCTCAT-3´	This study
GRMZM2G089836	5:67508591-67511890	Glycosyl hydrolase	fw 5´-CACTCCATCGTCGAGAGCTT-3´<br>rv 5´-GGCGTTGTTGAAGAGGAAGA-3´	This study

GRMZM2G156599	5:190674766-190677896	Yellow stripe 1 - phytosiderophore-Fe(III) importer	fw 5´-CCTTCGCCTTCGTCTTCA-3´<br>rv 5´-CCTGTTCACCTTCTCCCAGA-3´	1) / 2)
GRMZM2G465226	7:3483731-3484794	Pathogenesis related Protein 1 (PR-1)	fw 5´-TACGACCACGACACCAACAG-3´<br>rv 5´-AGCTGCAGATGATGAAGACG-3´	This study
GRMZM2G099049	7:120764735-120766193	Auxin / Dormancy related Protein	fw 5´-TTTTCCCCCAGTTCTCGTC-3´<br>rv 5´-CGTGCAGCTGCGAAATTAT-3´	This study
GRMZM2G067265	7:124863398-124868735	putative aminotransferase catalyzing the synthesis of methionine from 2-keto-methylthiobutyric acid	fw 5´-CTACAGCGCGAAGAAGGCTA-3´<br>rv 5´-GGGGTCGAGGTAGAACACG-3´	1) / 2)
GRMZM2G058451	7:155341756-155345811	putative bHLH Transcription factor (ILR3)	fw 5´-GGAGCAGCAGATCAAGTTCC-3´<br>rv 5´-CCAATCACAGGCATCATCAG-3´	2) / 3)
GRMZM2G336694	8:10989824-10990619	NA	fw 5´-CCCGGTTAAGATGTCCACTG-3´<br>rv 5´-AACCCATCAGCAAAGGTGTC-3´	This study
GRMZM2G153977	8:70995815-70998984	Eukaryotic aspartyl protease	fw 5´-AGCCAGGAGTGCAAGACTGT-3´<br>rv 5´-ACGCTACGAATTCCAGTGCT-3´	This study
GRMZM2G126010	8:100394854-100398658	Actin 1	fw 5´-ACCTCACCGACCACCTAATG-3´<br>rv 5´-GCAGTCTCCAGCTCCTGTTC-3´	Reference Gene qRT
GRMZM2G096029	8:104279085-104281118	Obtusifoliol 14α demethylase	fw 5´-GGCGGTAAGTTCACCTACGA-3´<br>rv 5´-CCCAATCTGTCTGAGGGAAA-3´	This study
GRMZM2G175661	8:151728613-151742221	Putative E3 Ligase (BRUTUS)	fw 5´-CGGTTTATTTTGGCATGCTT-3´<br>rv 5´-TGTTGTACGACCCACAGGAG-3´	2) / 3)
GRMZM2G049811	9:48437559-48441823	Formate Dehydrogenase	fw 5´-CTGGATACGGTGGTGATGTG-3´<br>rv 5´-GGCCTTCCTTGACGATGTAG-3´	This study/ 1) / 2)
GRMZM2G385200<br>GRMZM2G312481<br>GRMZM2G034956	9:135548861-135798318	Nicotianamine Synthase 1 genes	fw 5´-ACACCAAGGGGCTTCAGA-3´<br>rv 5´-TCGCCATCTCCTCTCTCTTC-3´	This study/ 1) / 2)
GRMZM2G178190	9:147509844-147513844	natural resistance associated macrophage protein 3	fw 5´-CCTCTCATCACCCTCGTTTC-3´<br>rv 5´-AGCAAAGGCGAGAATACAGC-3´	1) / 2)
GRMZM2G135536	10:893975-895713	Putative cytochrome P450	fw 5´-TGTTCCTCTGCAATGCTACG-3´<br>rv 5´-TTCACCTGCTCCGATGAAC-3´	This study
GRMZM2G133721	10:111856375-111861824	Putative Enolase	fw 5´-GAAAAGGCTGGCTACACTGG-3´<br>rv 5´-ACAGGTCTTTCAGGCTGTCG-3´	This study
GRMZM2G107306	10:123228974-123231540	Vacuolar iron tarnsporter 1	fw 5´-GTGTTCTGCATGTCGCTCAT-3´<br>rv 5´-CTGCTGAACATGGTGCTCAT-3´	1) / 2)
GRMZM2G464137	10:147402071-147405536	methylthioribose kinase	fw 5´-AAGCAATGGGGAGGCATAC-3´<br>rv 5´-GATCGTCTTCGCACAGTTGA-3´	1) / 2)

[a] fw = forward Primer, rv = reverse Primer

[1] Benke and Stich 2011

[2] Kobayashi and Nishizawa 2012

[3] Long et al., 2010
