# Supplementary figures and images for: Ups and downs of a transcriptional landscape shape iron deficiency associated chlorosis of the maize inbreds B73 and Mo17
Source: BMC Plant Biol. 2013 Dec 13;13:213. doi: 10.1186/1471-2229-13-213 (PMC3881016; doi:10.1186/1471-2229-13-213)

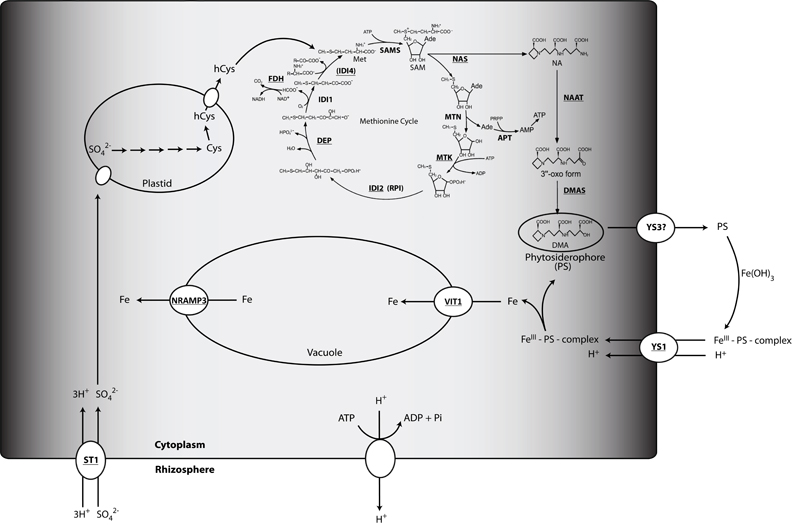

Supplement: Additional file 3 — MapMan Pathway file. JPG file for siderophore and Fe homeostasis related transcripts adopted from Benke et al.[5] without transcript bins. [file 1471-2229-13-213-S3.jpeg]
